# A Chemical Toolbox to Unveil Synthetic Nature-Inspired Antifouling (NIAF) Compounds

**DOI:** 10.3390/md22090416

**Published:** 2024-09-12

**Authors:** Ana Rita Neves, Sara Godinho, Catarina Gonçalves, Ana Sara Gomes, Joana R. Almeida, Madalena Pinto, Emília Sousa, Marta Correia-da-Silva

**Affiliations:** 1 Laboratory of Organic and Pharmaceutical Chemistry, Department of Chemical Sciences, Faculty of Pharmacy, University of Porto, Rua Jorge Viterbo Ferreira, 228, 4050-313 Porto, Portugal; 2 CIIMAR—Centro Interdisciplinar de Investigação Marinha e Ambiental, Terminal de Cruzeiros do Porto de Leixões, 4450-208 Matosinhos, Portugal

**Keywords:** biofouling, synthetic analogs, structure–activity relationship, antifouling

## Abstract

The current scenario of antifouling (AF) strategies to prevent the natural process of marine biofouling is based in the use of antifouling paints containing different active ingredients, believed to be harmful to the marine environment. Compounds called booster biocides are being used with copper as an alternative to the traditionally used tributyltin (TBT); however, some of them were recently found to accumulate in coastal waters at levels that are deleterious for marine organisms. More ecological alternatives were pursued, some of them based on the marine organism mechanisms’ production of specialized metabolites with AF activity. However, despite the investment in research on AF natural products and their synthetic analogues, many studies showed that natural AF alternatives do not perform as well as the traditional metal-based ones. In the search for AF agents with better performance and to understand which molecular motifs were responsible for the AF activity of natural compounds, synthetic analogues were produced and investigated for structure–AF activity relationship studies. This review is a comprehensive compilation of AF compounds synthesized in the last two decades with highlights on the data concerning their structure–activity relationship, providing a chemical toolbox for researchers to develop efficient nature-inspired AF agents.

## 1. Introduction: Marine Biofouling and Antifouling Biocidal Strategies

In the marine environment, submerged surfaces are naturally and quickly colonized by micro and macroorganisms, such as bacteria, algae, and invertebrate benthic species in a process designated “biofouling” [1,2], that induces several health and ecological risks as well as economic losses.

Marine biofouling concerns a complex and dynamic process which can be described in four main stages: (i) in the first minutes after surface submersion, the adsorption of ions and water forming an electric layer responsible for the further adhesion of organic molecules (proteins, polysaccharides, and proteoglycans) occurs, enabling the formation of a conditioning film; (ii) the conditioning film of organic material provides a surface for the settlement of bacteria, cyanobacteria, and diatoms through van der Waals forces and electrostatic interactions reinforced with the production of extracellular polymeric substances that vigorously stick them to the surface, providing the development of the biofilm layer in hours to days; (iii) within a few days, the attachment of algae and invertebrates larvae also occurs; and (iv) lastly, macroorganisms start to grow on the surface, which leads to a dense accumulation of robustly attached organisms [1,3]. However, this dynamic process is not linear and macroorganisms can settle before biofilms are formed, and depending upon the conditions, it takes weeks to months until a complete community of macroorganisms is established. Biofouling communities are quite unique and their biological constitution is hard to predict since they depends on several factors, such as the substratum, geographical location, light exposure, the season, and factors such as competition and predation [3].

While being a natural phenomenon, marine biofouling brings major consequences in economic, environmental, ecological, and safety aspects [4,5], and its control attracted increasing industrial and commercial attention, as it severely impacts maritime-related activities, namely shipping, aquaculture, oil and renewable energy offshores, and recreational activities. With the quadrupling of marine transport in persons and freight over the last thirty years, shipping now accounts for almost 90% of all global trade [6].

For the maritime industries, particularly shipping, marine biofouling leads to an increase in surface roughness, increasing the drag resistance of a ship moving through water, which increases fuel consumption and the production of greenhouse gases [5]. Regarding this impact on the increase in drag force of man-made immersed surfaces, marine biofouling species can be divided into three categories: (i) microfoulers (bacterial, fungal, and microalgal biofilms), which are responsible for 1–2% increase in drag force, (ii) soft macrofoulers (invertebrate larvae and algae), which accounts for a drag increase of up to 10%, and (iii) hard macrofoulers (barnacles, mussels, tubeworms, and bryozoans) leading to up to a 40% increase in drag force [2,3]. The impact of biofouling on the moving resistance of ships’ hulls is an active field of research [2,4,5,7,8,9,10]. The overall cost of hull fouling in the US Navy is projected to be over $1 million per ship per year, according to an economic analysis [5]. Moreover, fuel consumption would increase by as much as 40% if no antifouling (AF) measures were taken [11]. As a result, it is plausible that the maritime industries are keen to combat biofouling. Another biofouling-associated issue is the premature biocorrosion of the surfaces leading to the need of increasing the frequency of hull maintenance and consequently the costs.

From an environmental point of view, marine biofouling also causes the transportation of marine species from their native environment to a new environment where they can behave as an invasive species, unbalancing the local ecosystems [12,13]. This is a major threat to the conservation of biodiversity since invasive species will establish a reproductive population in the new environment, out-competing native species and multiplying into uncontrolled proportions [14]. Moreover, biofouling can contribute to increasing the density of environmental microplastics and promote the precipitation of plastic debris by deterioration of polymeric materials constituting the antifouling coatings [15].

**Figure 1:**
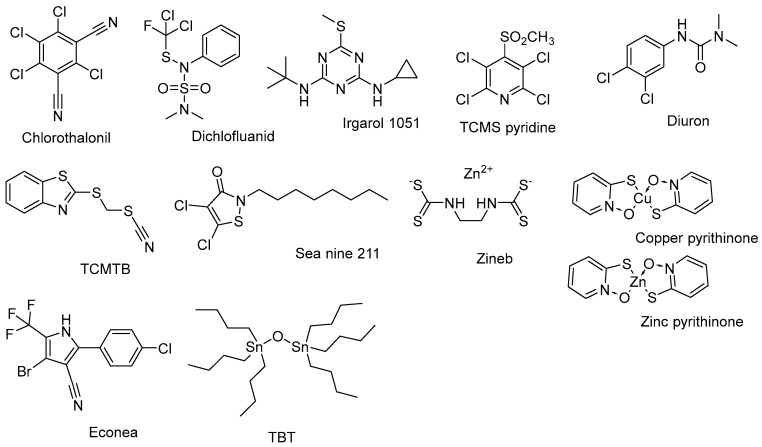
Examples of antifouling biocides. TCMS pyridine-2,3,5,6-Tetrachloro-4-(methyl sulphonyl); TCMTB—2-Thiocyanomethylthiobenzothiazole; and TBT-Tributyltin.

More than 2000 years ago, marine biofouling was already recognized as a problem, and therefore several attempts were made to overcome it. The first attempts to combat marine biofouling were conducted by early Phoenicians and Carthaginians and consisted of the use of pitch and possibly copper sheathing on ship’s bottoms [6]. It is estimated that the use of copper started around the 1600s, in the form of sheets. When compared to prior strategies, the use of copper sheets in AF coatings was effective; however, it was short-lived, and hull maintenance was required regularly (approximately every 18 months), which raised the allied costs. The replacement of copper sheets appeared in the mid 19th century. Essentially, they were paints consisting of copper pigments (copper, arsenic, and mercury oxides) incorporated in a variety of binders (linseed oil, shellac varnish, tar, and resins) [16]. In the early 1960s, organotin compounds, namely tributyltin (TBT) (Figure 1), were introduced as effective biocides in marine AF paints. Given that microalgae and amphora are tolerant to copper, while brown and green algae and certain diatoms are tolerant to organotin compounds, these two AF agents were used together. Almost 20 years after the discovery and application of TBT as an AF biocide, imposex in marine gastropod species was identified as a negative impact of TBT [17,18]. Indeed, several studies confirmed the correlation between the presence of high concentrations of persistent TBT in the marine environment and imposex, and also other detrimental effects in marine ecosystems, namely *Nucella lapillus* local extinctions and oyster malformations. This phenomenon is considered the best example of endocrine disruption in wildlife and was extensively studied by the scientific community over the years [19]. In 1989, the International Maritime Organization (IMO) recognized the deleterious effects of TBT in the marine environment, and in the next year, the IMO’s Marine Environment Protection Committee (MEPC) recommended that governments adopt measures to eliminate the use of TBT on vessels of less than 25 m in length. Later, in 1999, a global prohibition on the use of organotin compounds on ships by January 2003 and a complete prohibition by January 2008 was issued by IMO and MEPC [20]. More than 10 years after the TBT ban, it continues to be present in the marine environment, confirming its high persistence through accumulation in sediments, in several regions around the globe [21].

Following the TBT ban, copper began to be widely used in AF paints again, but with some restrictions concerning the release into the environment [6]. Although copper is naturally present in the marine environment and has an important role in the growth and metamorphosis of many marine organisms, when it exceeds the threshold of the organism’s tolerance, it becomes toxic [6].

Nowadays, 90% of AF paints in use contain at least 30% of copper, mostly in the form of cuprous oxide, raising concerns about copper pollution in the environment. However, booster biocides emerged as strengthening AF ingredients in copper AF paints. The most common booster biocides that appeared as “good” and less toxic alternatives to TBT were chlorothalonil, dichlofluanid, irgarol 1051^®^ (2-methylthio-4-tertiary-butylamino-6-cyclopropylamino-s-triazine), 2,3,5,6-tetrachloro-4-(methyl sulphonyl) (TCMS) pyridine, thiocyanatomethylthio-benzothiazole (TCMTB), diuron^®^ (1-(3,4-dichlorophenyl)-3,3-dimethylurea), sea nine 211^®^ (dichloro-octylisothiazolin), econea^®^ (tralopyril), zinc and copper pyrithione, and zineb (Figure 1). Most of them were used in agriculture as pesticides and herbicides with negative effects on the growth rate of photosynthetic organisms. However, it was shown that their negative impact was not limited to these organisms, having also negative effects on crustaceans, fishes, and invertebrates [22]. Therefore, their use is quite limited and banned in some countries [23].

Sea nine 211^®^ was considered for a while as an “eco-friendly” efficient booster biocide (awarded in 1996 with the “Green Chemistry Challenge Award” for its environmental safety as a marine AF agent). This statement was corroborated by studies that demonstrated that sea nine 211^®^ had excellent AF activity against a broad spectrum of fouling organisms and rapid degradation in seawater that allowed the decrease in concentration to levels below toxic concentrations [24]. However, it was proven that degradability is not reproducible around the world and depends upon the conditions, leading to increased concentrations of sea nine 211^®^ in the marine environment [25]. Additionally, endocrine-disrupting and reproductive-impairing effects were found for sea nine 211^®^ [25]. Along with the concern of the use of booster biocides in the marine environment, there are also concerns about the bioaccumulation potential of these agents with detrimental effects on human health via the food chain [23].

To accelerate the ban of these hazardous agents used to date, there is an urgent need to find harmless solutions to fight marine biofouling that could constitute, at the same time, effective and inexpensive alternatives [26,27,28]. A promising environmentally friendly strategy is the development of products based on the specialized metabolites produced by sessile marine organisms to prevent epibiosis [29]. Although these marine natural compounds are not free of being potentially toxic, they were selected during evolution to have high specificity, high efficiency, and to be biodegradable. Unfortunately, the extraction of these marine natural products (MNPs) is not environmentally sustainable, often yielding low quantities, which hampers their future development. On the other hand, due to the structural complexity of most of MNPs, their total synthesis often needs several steps, which incurs high costs and are time consuming [30]. The synthesis of nature-inspired molecules may overcome this bottleneck issue of MNP.

## 2. Synthesis of Nature-Inspired AF Compounds

Nature-inspired compounds were synthesized as AF candidates with low or no toxicity to the environment [31,32,33,34]. The design of synthetic chemical analogs of natural molecules provides valuable information on the structure–activity relationship (SAR). By adding or deleting chemical groups/moieties, it is possible to identify which molecular sections influence the biological activity and consequently optimize the chemical structure either to increase the AF properties or to simplify the natural structure.

**Figure 2:**
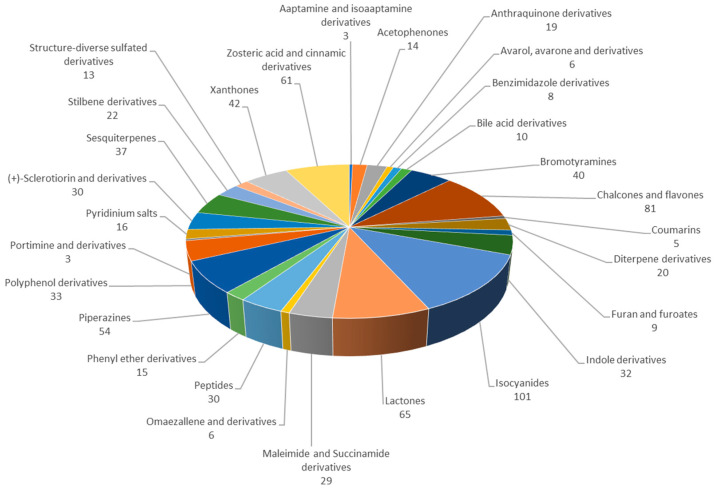
Synthetic compounds analyzed in this review distributed by chemical classes.

In this review, we analyzed SAR conclusions obtained from nearly 800 synthetic compounds from different chemical classes (Figure 2). These molecules will be systematically presented by chemical classes and in alphabetical order, and the most relevant SAR studies and AF mechanisms disclosed will be highlighted. In this view, any researcher interested in developing potent non-toxic AF compounds can find detailed information about all the studies performed until now and hints of the scaffolds and molecular modifications that can be applied to achieve their goal.

**Figure 3:**
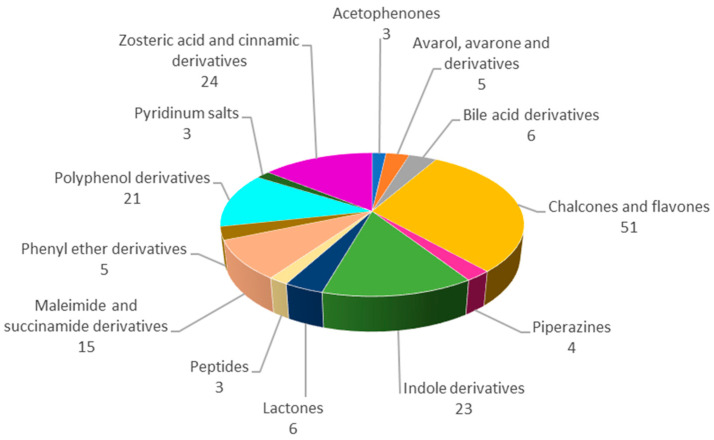
Number of synthetic compounds with antibacterial activity distributed by chemical classes.

**Figure 4:**
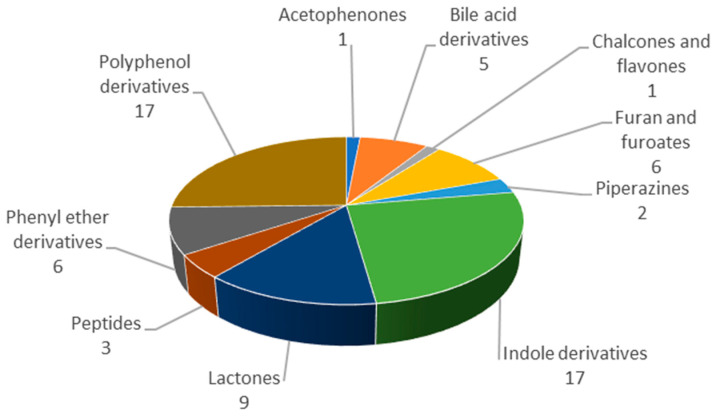
Number of synthetic compounds with antialgal activity distributed by chemical classes.

**Figure 5:**
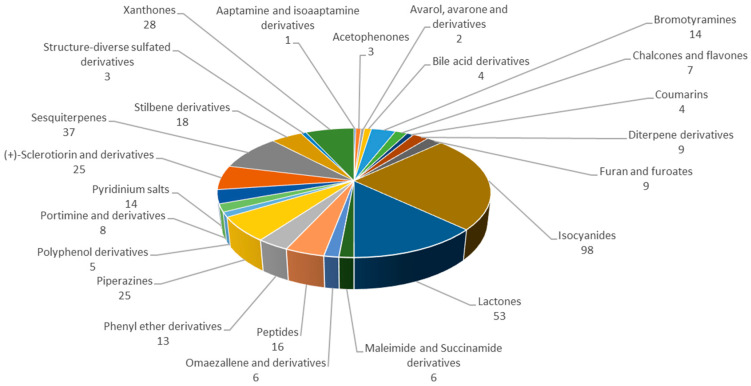
Number of synthetic compounds with anti-macrofouling activity distributed by chemical classes.

**Figure 6:**
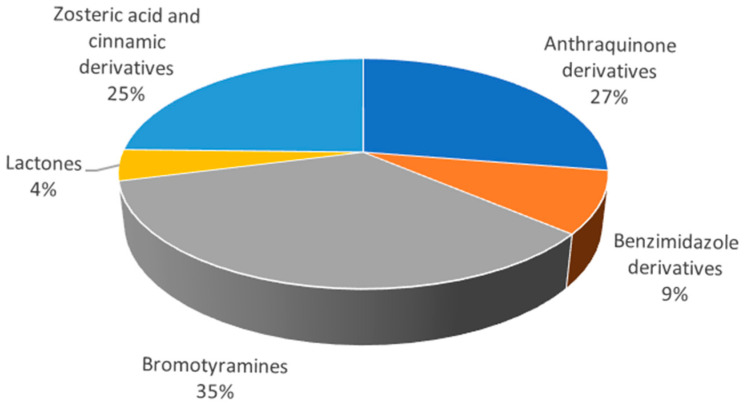
Chemical classes of synthetic compounds with antibiofilm activity.

The great diversity of biofouling species makes it important to find a compound capable of acting on several organisms of the fouling community. AF assays were performed over a wide range of organisms, namely bacteria (Figure 3), algae (Figure 4), and invertebrates (Figure 5). Barnacle, specifically *Amphibalanus* (=*Balanus*) *amphitrite*, was the most tested marine fouling organism, which can be explained by its wide distribution. Other works used mussel species as a target organism. Following this, biofilm-forming marine bacteria and fungi (Figure 6) were also largely used in AF studies, as biofilms are important for the attachment of macrofoulers.

To be selected as a new promising AF compound, the new agents must be proven to be effective and non-toxic. For that, the EC_50_ (half-maximal effective concentration) and the LC_50_ (lethal concentration, 50%) must be determined and the therapeutic ratio (LC_50_/EC_50_) calculated to be used as an index for an AF agent potential. Compounds are considered promising non-toxic AF compounds when they exhibit LC_50_/EC_50_ > 15 and EC_50_ < 5 µg·mL^−1^, although a much higher therapeutic ratio > 50 should be used when selecting candidate AF compounds [32,35]. In addition to efficacy and toxicity aspects, promising antifouling compounds should reveal compatibility with marine coatings [36].

### 2.1. Aaptamine and Isoaaptamine Derivatives

**Figure 7:**
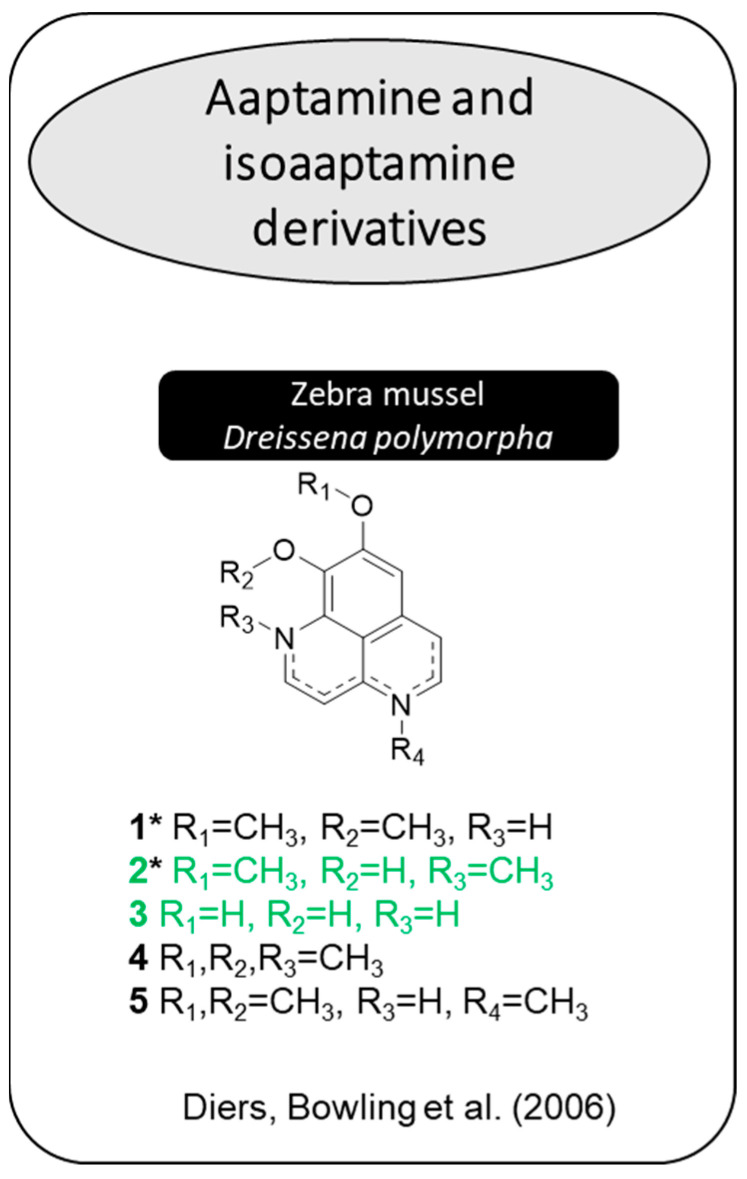
Aaptamine (**1**) and isoaaptamine (**2**) and synthetic derivatives **3**–**5** [37]. * Natural products; green numbers: most active compounds.

Aaptamine (**1**) and isoaaptamine (**2**) are alkaloids found in the sea hare, *Aplysia punctata* [37]. Both, as well as synthetic analogs **3**–**5** (Figure 7), were evaluated against the inhibition of the settlement of the zebra mussel (*Dreissena polymorpha*). Aaptamine analogs **2** and **3**, which have one or two hydroxy groups, were the most potent compounds, inhibiting the settlement of zebra mussels with an EC_50_ = 11.6 and 18.6 µM, respectively. The importance of hydroxy groups for AF activity was confirmed by compounds **4** and **5**, which were not active, compared to compound **2**. Toxicity against a non-target aquatic organism (duckweed) was related to the methoxyl substitution pattern, as compound **3** did not show toxicity when compared to compounds **1** and **2** This work was the first report of aaptamines as promising AF candidates.

### 2.2. Acetophenones

**Figure 8:**
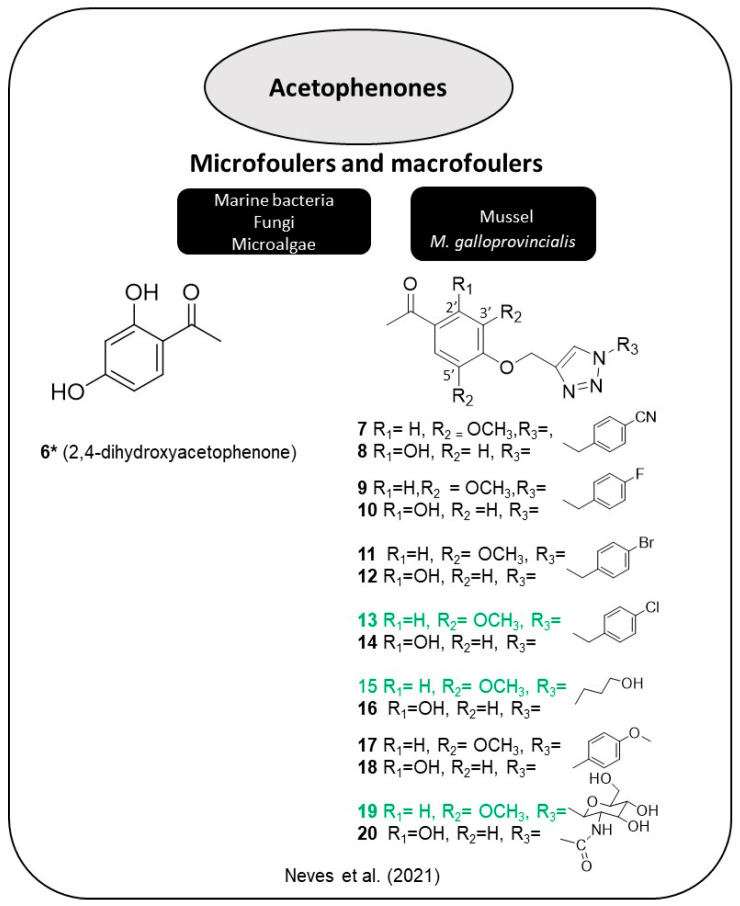
Structures of new 1,2,3-triazole tethered acetophenones [38]. * Natural products; green numbers: most active compounds.

A natural compound, 2,4-dihydroxyacetophenone (**6**, Figure 8), showed robust inhibition of *Ulva pertursa* and a relevant decrease in fouling biomass when incorporated in a controlled depletion paint [38]. On the other hand, triazole derivatives are remarkable compounds in medicinal chemistry, showing a wide range of bioactivities, such as antimicrobial. Considering the relevance of both 1,2,3-triazole and benzo/acetophenone moieties, a library of 14 new acetophenone-1,2,3-triazole hybrids (**7**–**20**, Figure 8) containing different substitution patterns, such as aromatic nitriles (**7** and **8**), aromatic halogens (**9**–**14**), aliphatic alcohols (**15** and **16**), and N-acetylglucosamines (**19** and **20**), were synthesized through the copper(I)-catalyzed alkyne-azide Huisgen cycloaddition, a “click” reaction [38].

The most promising compounds against the settlement of a heavy macrofouler, mussel *Mytilus galloprovincialis* larvae, were three compounds containing methoxy groups in the phenyl ketone core with different substituents linked to the heterocyclic ring. In particular, compounds **13** (EC_50_ = 11.20 µg·mL^−1^/28.87 µM) with an aromatic chlorine, **15** (EC_50_ = 13.46 µg·mL^−1^/40.14 µM) with an aliphatic alcohol, and compound **19** (EC_50_ = 9.94 µg·mL^−1^/20.68 µM), an acetophenone derivative with an acetamide glucose moiety. It is crucial to observe that none of these three compounds caused mortality to the target species *M. galloprovincialis* plantigrades at the highest concentration tested (200 µM), and revealed an EC_50_ < 25 µg·mL^−1^, a recommended value by the U.S. Navy program for antifoulants. Regarding SAR, the presence of two methoxy groups at C-3′ and C-5′ on the phenyl ketone core proved to be more favorable than the presence of hydroxyl groups at C-2′ for the mussel larvae anti-settlement activity [38].

**Scheme 1:**
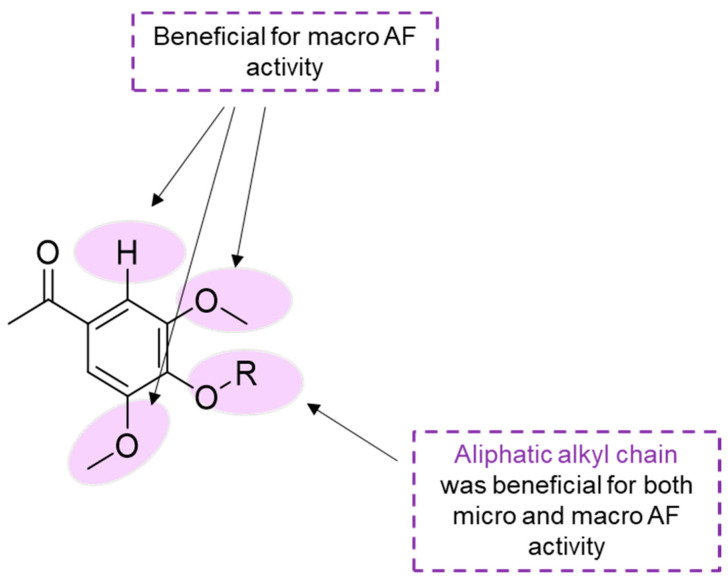
SAR for acetophenones.

These acetophenones were also evaluated for their ability to inhibit the growth of marine biofilm-forming bacteria of five strains (*Vibrio harveyi*, *Cobetia marina*, *Halomonas aquamarina*, *Pseudoalteromonas atlantica*, and *Roseobacter litoralis*), fungi (*Candida albicans*, *Aspergillus fumigatus*, and *Trichophyton rubrum*), and microalgae (*Navicula* sp.) [38]. Compounds **8**, **10**, and **16** were shown to robustly inhibit *R. litoralis* growth. Regarding SAR, the presence of hydroxyl groups at C-2′ on the phenyl ketone core appears to increase the antibacterial activity. None of the compounds tested revealed activity against the fungal strains, with MICs higher than the maximum tested concentration (128 µg·mL^−1^). The most promising compounds with antifouling activity (**13**, **15**, and **19**) were evaluated for their ability to inhibit the growth of the biofilm-forming marine diatom *Navicula* sp. Acetophenone **15** showed inhibitory activity against *Navicula* sp. with a EC_50_ of 26.73 µM, which suggests a complementary action of this compound against macro and microfouling species and also reinforces the potential of this compound as an AF agent [38]. Some insights of the SAR for acetophenones are evidenced on Scheme 1.

Congruent to find eco-friendly compounds, the ecotoxicity and bioaccumulative potential of compounds **13**, **15**, and **19** were also assessed. The compounds were found to be less toxic to crustacean *Artemia salina* at both concentrations tested (25 and 50 µM) than the commercial biocide econea^®^ (100% lethality), with the mortality rates of acetophenones **15** and **19** not significantly different from the negative control. Acetophenones **15** and **19** showed a LogKow value lower than 3 (in silico prediction), the threshold value from which compounds are considered bioaccumulative, which indicates their low bioaccumulative potential. In this line of thinking, compounds **15** and **19** could be considered hits for the development of effective and eco-friendly AF compounds [38].

### 2.3. Anthraquinone Derivatives

**Figure 9:**
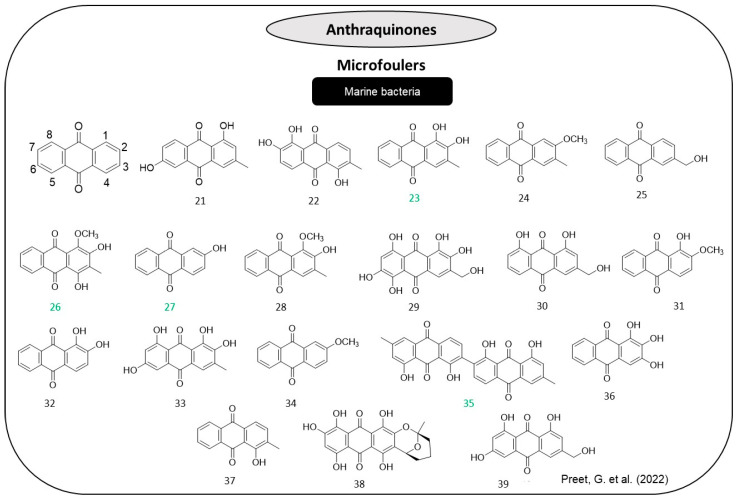
Chemical structures of anthraquinone derivatives [39]. Green numbers: most active compounds.

Anthraquinones (AQs) are chemical scaffolds characterized by a 9,10-dioxoanthracene core structure substituted by three fused benzene rings with two ketone functional groups on the central ring. Until now, about 700 molecules with AQ skeletons were characterized, of which about a third were isolated from plants, while most were isolated from bacteria, lichens, fungi, and sponges or other marine invertebrates [39]. Due to the diversity of biological properties, AQs are a relevant class of bioactive compounds and there is strong evidence of their potential interest for use as additives in AF coatings to prevent marine biofouling. Specifically, citreorosein and emodin were described to reveal robust AF activity against the settlement of *A. amphitrite* larvae [39]. On this wise, Preet and colleagues selected 19 structurally distinct AQ compounds (**21**–**39**, Figure 9), based on the same anthraquinone core structure, and studied them regarding their microbial growth and biofilm adhesion inhibition activity against three marine bacterial species which are key players in the marine biofilm process: *Vibrio carchariae*, *Pseudoalteromonas elyakovii*, and *Shewanella putrefaciens* [39].

All AQ analogs were effective at inhibiting the biofilm growth of *P. elyakovii* at a very low concentration (20% to 56% at 0.001 μg·mL^−1^). Furthermore, 60% of the compounds reveal a minimal inhibitory concentration (MIC) above 10 μg·mL^−1^, which shows that although the overall growth of the microbes is not affected between 10 μg·mL^−1^ and 0.001 μg·mL^−1^, the biofilm adhesion is affected by these compounds. A biofilm adhesion MIC of 0.01 μg·mL^−1^ was observed for **26**, **27**, and **35** against *V. carchariae*, while in general, the compounds tested exhibited a larger degree of variation in their activity against this bacterium. Curiously, the commercially applied compound **30** showed one of the highest microbial growth and biofilm adhesion MICs against *V. carchariae* of 10 μg·mL^−1^ [39].

**Scheme 2:**
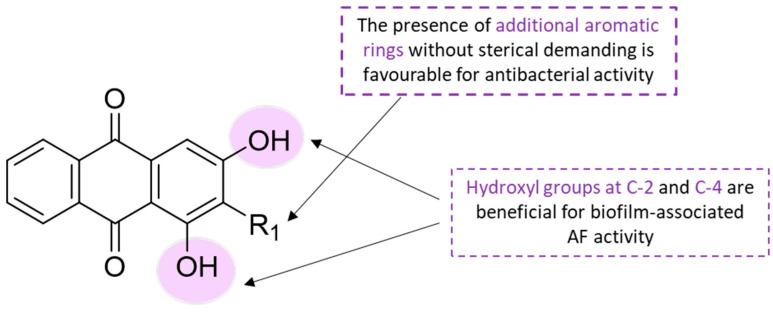
SAR for Anthraquinone derivatives.

Additionally, SAR of the best-performing compounds were evaluated to conclude the structural properties which are crucial in contributing to the biofilm-associated AF activity. The presence of phenolic hydroxyl (OH) groups at positions 2 and/or 4 of the AQ skeleton provides the lowest MIC of 0.01 μg·mL^−1^ (compound **26**). In a structural vision, compared with the other compounds investigated in this study, compounds **35** and **38** were the most distinct. In this way, the pronounced activity of compound **35** (0.01 μg·mL^−1^ against *V. carchariae*) can be attributed to the presence of additional aromatic rings within the structure, while the presence of an additional sterically demanding heterocyclic ring system in compound **38** was conducted to decreased activity (0.1 μg·mL^−1^) [39]. Some insights of SAR for anthraquinones are summarized in Scheme 2.

Because of the absence of molecular targets identification and the unknown mechanism of action of AF compounds, the research of potential mechanisms that explain how compounds are inhibiting biofim adhesion becomes a challenge. In this point of view, the authors proposed the hypothesis that the interruption of the quorum sensing signaling system associated with the production of biofilms in *V. carcharie* could be involved in the mechanism of action for the AF activity observed [39]. In this way, a molecular docking study was performed to evaluate the differences in binding between the compounds and a crucial protein involved in the transportation of autoinducers which are signaling compounds within the quorum sensing system, the LuxP protein. Compounds **21**, **27**, **30**, and **39** revealed the best docking to the receptor site with binding energies ranging between −8.4 kcal/mol and −7.7 kcal/mol [39]. It deserves to be highlighted that one of the most active AQs in the MIC study was compound **27**, so the authors speculated that the compound’s activity may be based on binding to LuxP protein. A pharmacophore model was created based on the four best docked AQs: **21**, **27**, **30**, and **39**. The generated pharmacophore revealed three main features: hydrogen bond acceptors (HBAs), hydrogen bond donors (HBDs), and aromatic rings. The common feature pharmacophore model with a score of 0.9242 showed certain features: two HBDs, four HBAs, and two aromatic rings [39]. In a subsequent work, Preet and colleagues aimed to identify the pharmacophore of anthraquinones with antifouling activity by targeting LuxP protein [40]. For that, the authors performed a virtual screening using a dataset of naturally occurring anthraquinones-related compounds against LuxP protein of *V. carchariae* and found that there are six possible pharmacophoric features important for AF activity, particularly hydrophobic interactions, HBAs, HBDs, aromatic interaction, negative ionisable area interaction, and positive ionisable area interaction, that may guide the selection, design, and synthesis of anthraquinone derivatives [40].

### 2.4. Avarol, Avarone and Derivatives

**Figure 10:**
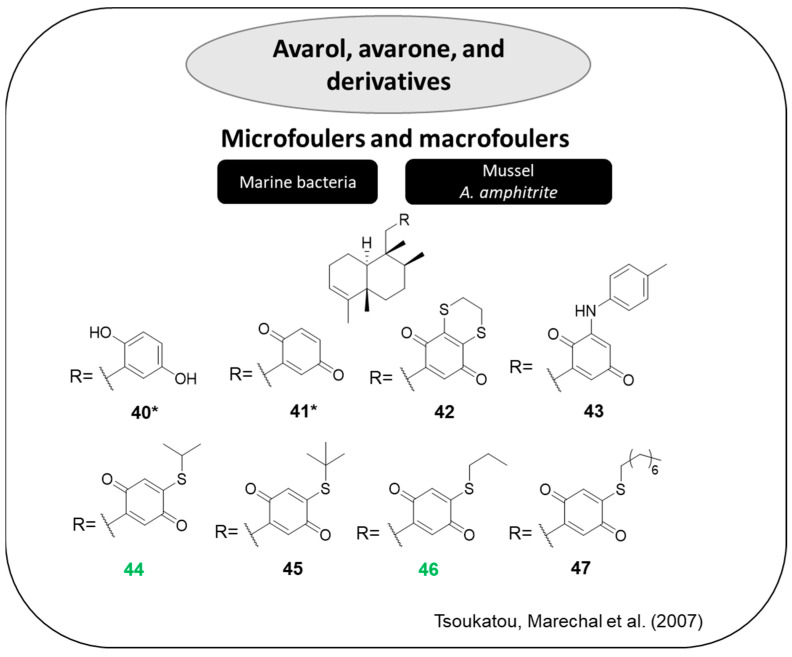
Avarol (**40**), avarone (**41**), and derivatives **42**–**47** [41]. * Natural products; green numbers: most active compounds.

The antifouling activity of avarol (**40**), isolated from the marine sponge *Dysidea avara*, and avarone (**41**), obtained by oxidation of **40** was studied against *A. amphitrite* (anti-settlement activity) and against four species of marine bacteria (growth inhibitory activity) along with several lipophilic derivatives (**42**–**47**, Figure 10) synthesized to reduce solubility in seawater and to improve their incorporation in a potential AF coating [41]. The most active compounds against the settlement of *A. amphitrite* were **41** (EC_50_ = 0.65 μg·mL^−1^) and compounds **42** and **46** (EC_50_ = 0.65 and 0.45 μg·mL^−1^, respectively). Interestingly, while mortality was observed for the two NPs avarol (**40**) and avarone (**41**), synthetic compounds **42**–**47** showed no toxicity at the maximal tested concentration (50 μg·mL^−1^). All tested compounds, except compound **43**, showed significant antibacterial activity. Synthetic compounds **44** and **46** showed the most potent antibacterial activity. Steric hindrance exhibited by bulky substituents on the quinone ring significantly decreased antimicrobial activity (**42**, **43**, and **45**). Overall, synthetic compounds **44** and **46** were the most promising compounds due to their both anti-settlement and antimicrobial activities at concentrations that were not acutely toxic to barnacle larvae (therapeutic ratio > 40). More sesquiterpene compounds can be found in Section 2.24.

### 2.5. Benzimidazole Derivatives

The benzimidazole nucleus is a remarkable pharmacophore in drug discovery and therapeutic applications [42]. On the other hand, nucleoside mimetics attracted considerable attention in medicinal chemistry due to their biological and chemotherapeutic properties. *C*-nucleosides are stable to hydrolytic and enzymatic cleavage and are clinically used as strong inhibitors of viral polymerases in the treatment of HCV (hepatitis C) and HIV.

**Figure 11:**
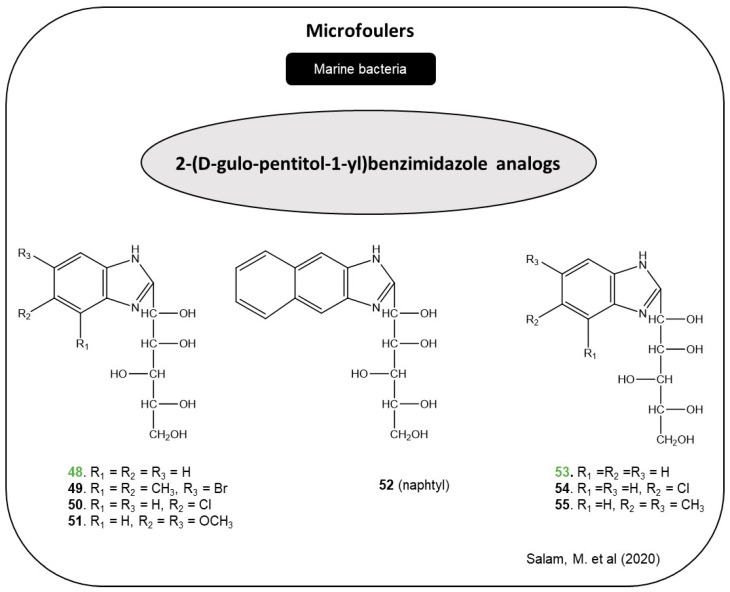
Structures of 2-(d-gulo-) and 2-(d-gluco-pentitol-1-yl)benzimidazole analogs [42]. Green numbers: most active compounds.

Eight benzimidazole *C*-nucleosides were synthesized (Figure 11): five 2-(d-gulofuranosyl)benzimidazoles (**48**, **49**, **50**, **51**, and **52**) and three 2-(d-glucofuranosyl)benzimidazoles (**53**, **54**, and **55**). These compounds and their precursor lactones (d-gulonic and d-gluconic acid-*γ*-lactone) and amines (*O*-phenylenediamine dihydrochloride, 3-bromo-4,5-dimethyl-*o*-phenylenediamine dihydrochloride, 4-chloro-*O*-phenylenediamine dihydrochloride, and 4,5-dimethyl-o-phenylenediamine dihydrochloride) were incorporated in a marine paint formulation. The prepared panels were immersed in the seawater and the compounds were investigated for their antifouling properties using an antibacterial biofilm test [42].

The acyclic-gulo and gluco-analogues revealed a decrease in the biofilm bacterial number compared to marine paint formulation without any additives and the commercial marine paint “Sipes”. The best treatments were with compounds **48**, **49**, **50**, **51**, **53**, and **54**, which showed a significant decrease in the biofilm bacterial number related to the marine paint formulation without any additives and both positive controls, suggesting that these compounds were useful for inhibiting marine bacterial growth. Moreover, the tested compounds showed better antibacterial biofilm property than their precursor amines, which suggests that these analogues are good candidates for development of new AF paints [42]. SAR analysis revealed that inhibiting the bacterial growth could be accomplished by following sequence of compounds: unsubstituted compounds (compounds **48** and **53**) > 5(6) chloro-analog (compound **50**) > 5,6-dimethyl analog (compound **51**) > 5(6)-chloro- analog (compound **54**) > 6-bromo-4,5-dimethyl- analog (compound **49**) [42].

### 2.6. Bile Acid Derivatives

Among the plethora of structurally diverse steroids isolated from marine invertebrates [43,44], there are only a few reports of bile acids and derivatives isolated mainly from octocorals and sponges [45]. These rare findings of bile acid derivatives in marine invertebrates show an interesting structural feature, the increased lipophilicity, for example, by acetylation of their polar compounds, which decreases their water solubility and allows a high concentration of the bioactive compound on the surface of the organism. In this way, this structural feature may constitute a requirement for its ecological function, which is especially interesting in the case of interactions that take place at the surface of the invertebrate, such as acting as a natural shield against epibiosis [45]. Specifically, peracetylated cholic acid, a natural biodegradable bile acid derivative isolated from the Patagonian sponge *Siphonochalina fortis*, was evaluated in laboratory and field AF trials. The results reveal that peracetylated cholic acid exhibited AF activity and low toxicity against the mussel *Mytilus edulis platensis*. Moreover, the experimental soluble matrix paints additivated with 0.6% (*w*/*w*) showed promissory performances in the in situ sea field trials [45].

**Figure 12:**
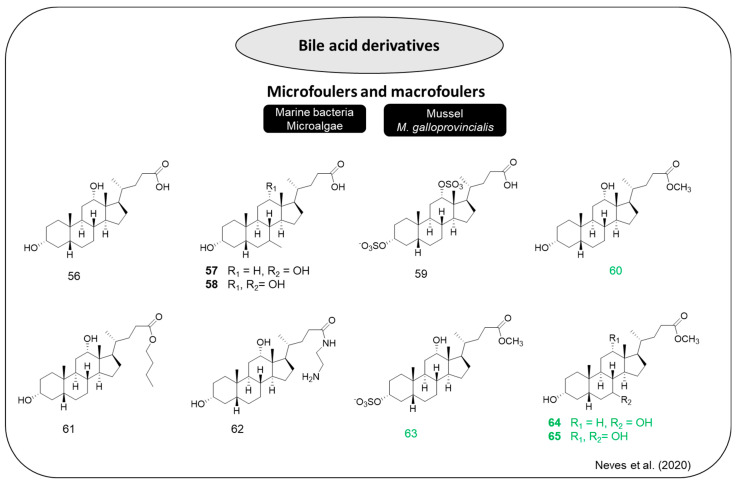
Bile acid derivatives [46]. Green numbers: most active compounds.

**Scheme 3:**
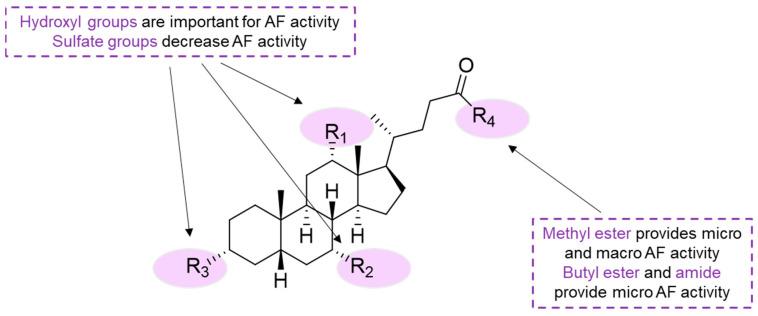
SAR for bile acid derivatives.

To explore the interest of bile acid scaffolds in the development of new antifouling compounds, a series of derivatives of three bile acids (deoxycholic acid, chenodeoxycholic acid, and cholic acid) with diverse polarities and with the ability to form both hydrophobic and electrostatic interactions was planned and synthesized (Figure 12) [46]. The AF effects of the synthesized derivatives, as well as of the three parent bile acids, were assessed through a set of AF bioassays, including antimacrofouling tests against the mussel *M. galloprovincialis* and antimicrofouling tests against five biofilm-forming marine bacteria and four representative biofouling microalgae species. The bile acid **56** and derivatives **60**–**64** showed robust bacterial growth inhibition, revealing inhibitory values around 40% at concentrations of 12.5 μM and *R. litoralis* was the most sensitive species, specially to compounds **60** and **62**. Concerning to microalgal, bile acids **56** and **57** and derivatives **60**, **63**, and **64** inhibited the growth of all the diatom species. It is important to note that compounds **56**, **60**, and **62**–**64** presented EC_50_ values between 3 μM and 10 μM for all the tested species, except for *Navicula* sp. The most potent bile acid against the settlement of *M. galloprovincialis* larvae was the methyl ester derivative of deoxycholic acid (**65**) (EC_50_ = 3.7 μM; LC_50_ > 200 μM; and LC_50_/EC_50_ > 50) followed by methyl ester derivatives of chenodeoxycholic acid (**64**) and cholic acid (**60**). The lengthening the side chain in ester derivative of deoxycholic acid (**61**) did not lead to an increase in the AF activity, but it appears to affect the broad spectrum to a species-specific profile considering that compounds **60** and **63**–**65** showed AF activity against micro and macrofouling species, and that compound **61** only inhibited the bacteria *R. litoralis* and diatom *Halamphora* sp. Moreover, sulfation at position 3 did not affect pronouncedly the AF activity, but increased the solubility of compound **60** in water. It is also relevant to analyze that compound **59**, without free hydroxyl groups, did not show any AF activity, which suggests that free hydroxyl groups in the bile acid scaffold may be important to AF activity. In contrast, structural modifications such as esterification (compounds **60**, **61** and **63**–**65**) and the presence of a primary amine (compound **62**) increased the inhibitory activity against the growth of diatoms and marine biofilm-forming bacteria (Figure 12) [46]. A resuming of using the SAR for bile acid derivatives is evidenced in Scheme 3. Furthermore, compounds **63** and **65** demonstrated better inhibition activity than econea^®^ against *Halamphora* sp., and *Cylindrotheca* sp., respectively. With regard to eco-toxicological aspects, compounds **60** and **63**–**65** were not toxic to *A. salina* and compound **63** showed the lowest bioaccumulation potential [46]. Bile acid derivative **60** was selected for direct incorporation in two polymeric coatings, polydimethylsiloxane (PDMS) and polyurethane (PU), and showed good compatibility with both systems. The anti-settlement activity on *M. galloprovincialis* larvae of this bile acid derivative was maintained even after incorporation. These results indicate that **60** is a good candidate for further in situ testing [46].

In the direction to elucidate de mechanism of action of bile acid derivatives, the in vitro inhibition of acetylcholinesterase (AChE), which is known to play an important role in the settlement of macrofouling organisms, was evaluated for the compounds that revealed an EC_50_ < 10 μM in the settlement of *M. galloprovincialis* (compounds **60** and **63**–**65**), and only in the presence of derivative **64**, the activity of this enzyme decreased slightly [46].

### 2.7. Bromotyramines

Bromotyrosine-derived MNPs are frequently isolated from sponges and ascidians [47]. Moloka’iamine (**66**), a compound first isolated in 1993, is based on an *O*-alkylated dibromotyramine core and displays cytotoxic and AF activities [48]. Particularly, compound **66** is well-known due its robust AF activity against barnacle crypids of *A. amphitrite*. Nearly 30 natural bromotyramines and their synthetic derivatives were reported in the literature as follows.

**Figure 13:**
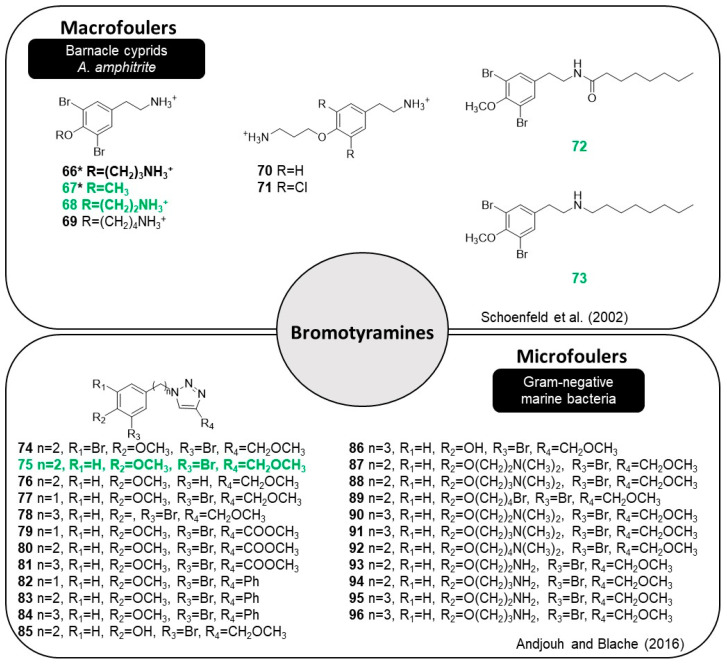
Moloka’iamine (**66**), bromotyramine (**67**), and synthetic derivatives **68**–**96** [49,50]. * Natural products; green numbers: most active compounds.

Analogs of hydrochlorides of natural bromotyramines **66** and 3,5-dibromo-4-methoxy-b-phenethylamine (**67**) were synthesized (**68**–**73**, Figure 13) to establish SAR for this relatively simple core structure [49].

The substitution of the bromines with hydrogens and chlorines in **70** (EC_50_ > 50 µg·mL^−1^) and **71** (EC_50_ = 33 µg·mL^−1^), respectively, led to a decrease in AF activity against barnacle *A. amphitrite*, indicating that bromines were strictly required for AF performance. Compound **68** (EC_50_ = 0.8 µg·mL^−1^) was the most active compound tested among the analogs of bromotyramine **66**–**73**. The authors also investigated the AF activity of compound **67** (EC_50_ = 0.07 µg·mL^−1^) and found that this natural compound is the most potent AF bromotyramine reported to date. The structure of bromotyramines **72** and **73** was designed to hybridize the aromatic portion of **67** with an aliphatic portion to obtain a more lipophilic derivative of **66** that would be more soluble in marine coatings (lipophilic) with a solvent base, and therefore more practical as an AF marine paint additive. Compounds **72** (EC_50_ = 0.2 µg·mL^−1^) and **73** (EC_50_ = 0.008 µg·mL^−1^) potently inhibited the settlement of barnacle cyprids, and the AF performance of compound **73** was far superior to the AF performance of the natural compound **67**. Regarding cyprid toxicity, bromotyramines **67**, **72**, and **73** exhibited different levels of toxicity which may be attributed to the observed AF activity [49].

**Scheme 4:**
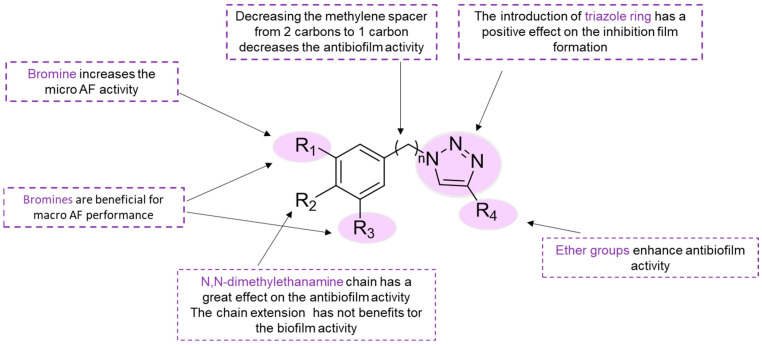
SAR for bromotyramine derivatives.

In another study, 23 analogs based on the *O*-alkylated dibromotyramine core were synthesized through the application of click chemistry (**74**–**96**, Figure 13) on an appropriately large scale [50]. Compounds **74**–**76** comprise the bromotyramine core bound to the methyl ether group through a 1,2,3-triazole. The antibiofilm activities in three Gram-negative marine bacteria, *Pseudoalteromonas ulvae*, *Pseudoalteromonas lipolytica*, and *Paracoccus* sp. were compared to compounds **66** and **67**, the natural bromotyramines, to understand the effect of the triazole ring. It was possible to infer that most of the bromotyramine derivatives were more active than **66** and **67** against biofilm formation by the three bacterial strains. Regarding to SAR, the antibiofilm activity of compound **74** suggests that the introduction of a triazole ring increases the inhibition of biofilm formation. The absence of the bromine atom at the 5-position on the aromatic ring in **75** slightly increased the activity against all strains. Monobrominated derivative **75** was selected as a starting point to study the impact of the length of the chain between the triazole and aromatic rings on the antibiofilm activity. Decreasing the methylene spacer from two carbons (**75**) to one carbon atom (**77**) resulted in a decrease in antibiofilm activity. The addition of one methylene group between the aromatic and triazole rings (**78**) enhanced activity against *Paracoccus* sp., but surprisingly led to a decrease in activity against *P. ulvae* and *P. lipolytica*. Following, an evaluation of the impact of the nature of substituents on the 4-position of the triazole was conducted. The replacement of the ether group of compounds **75**, **77**, and **78** with a methyl ester or phenyl in compounds **79**–**81** and **82**–**84**, respectively, led to a substantial decrease in the antibiofilm activity against all strains. From this point, bromotyramines **75** and **78** were the most promising compounds and more analogs were synthesized (**87**–**96**) to understand how modifications of the aromatic core would change the antibiofilm activity. Replacing a methyl group by a *N*,*N*-dimethylethanamine chain (**87**) increased the antibiofilm activity and lowered the values of the EC_50_ to less than 100 μM. Analogs with a longer chain were synthesized (**88**–**92**), including primary amine derivatives (**93**–**96**), but the extension of the chain did not bring any benefits for the antibiofilm activity. A primary amine was introduced instead of a tertiary amine in compounds **87**, **88**, **90**, and **91** to generate compounds **93**–**96**. Replacement of dimethylamine by a primary amine was prejudicial to the antibiofilm activity. The information obtained with the SAR for this class of compounds, such as the essential presence of bromine and the presence of a triazole ring, might help researchers to develop more potent antibiofilm compounds. Toxicity assays confirmed that the antibiofilm activity of all compounds, except for compound **89**, was not a result of a bactericidal effect [50]. Some insights of the SAR for bromotyramine derivatives are evidenced in Scheme 4.

**Figure 14:**
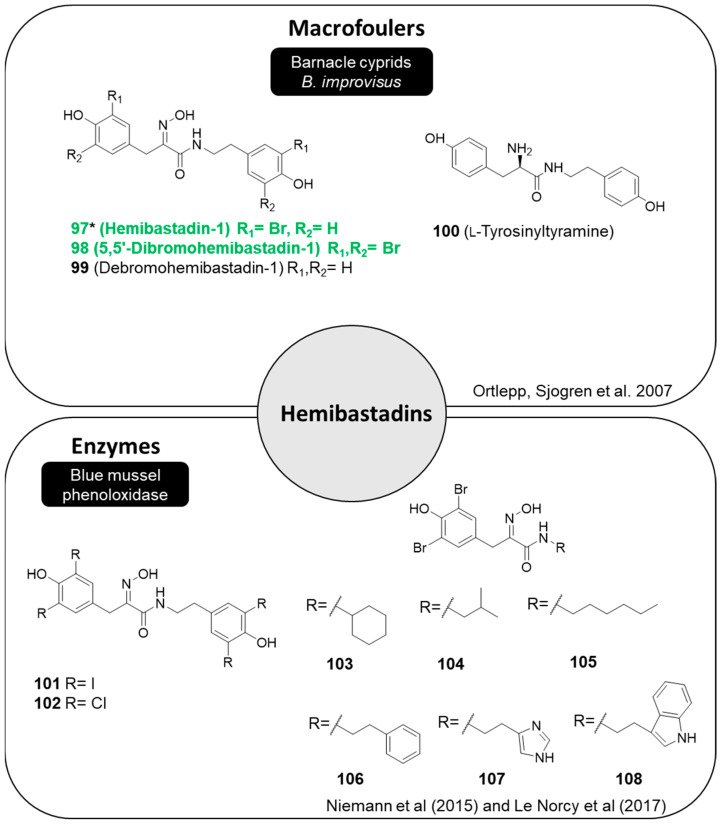
Hemibastadin (**53**) and synthetic derivatives (**98**–**108**) [51,52]. * Natural products; green numbers: most active compounds.

Hemibastadins are typical specialized metabolites of the Pacific elephant ear sponge *lanthella basta* and consist of a brominated tyrosine moiety featuring an oxime function instead of the amino group and a likewise brominated tyramine unit linked to tyrosine through an amide bond [51]. In 2007, Ortlepp and colleagues reported the AF activity of 15 brominated NPs isolated from several marine sponges and three synthetic analogs [52]. These compounds were tested against the inhibition of *Amphibalanus improvisus* cyprid settlement. Among the NPs present in the study, hemibastadin-1 (compound **97**) was selected as a lead compound for the preparation of synthetic analogs **98**–**100** (Figure 14). Through these analogs, the importance of the oxime substituent and the bromide atoms for the AF activity was studied.

The removal of the oxime function (compound **100**) and bromine (compound **99**) resulted in a decrease in the activity, which indicates that the presence of the oxime function and bromine modulates the activity of the respective derivatives. Interestingly, while the natural compound **97** caused significant larval mortality at low concentrations, no toxicity was observed for its synthetic analogs, especially compound **98**, which exhibited the same potency as the natural compound **97**. Hemibastadin-1 (**97**) and synthetic products **98**–**100** showed no general toxicity when tested against brine shrimp (*A. salina*) larvae. Synthetically derived 5,5′-dibromohemibastadin-1 (**98**) was later found to be a potent inhibitor of blue mussel *M. edulis* phenoloxidase (IC_50_ = 0.8 µM), an enzyme involved in the firm attachment of this invertebrate to substrates [53]. The oxime function of hemibastadins, which was shown to be important for the anti-settlement displayed by compound **98**, is responsible for the strong copper-chelating properties, thereby presumably causing enzyme inhibition. Furthermore, the presence of bromine substituents at the phenolic rings of compound **98** increased the enzyme inhibitory properties [53].

Later, to investigate the enzyme inhibitory activity of hemibastadin derivatives, nine new compounds (**101**–**108**, Figure 14) were synthesized with structural variations regarding compound **98**’s core structure, namely, different halogen substituents present at the aromatic rings (**101** and **102**) and different amine moieties linked to the (*E*)-2-(hydroxyimino)-3-(4-hydroxyphenyl)propionic acid (**103**–**108**) [51]. According to the results obtained, it was possible to establish that the presence of bulky halogen atoms ortho to the phenolic hydroxy groups increases the inhibitory activity [51]. Overall, derivatives structurally close to sponge-derived hemibastadins revealed superior enzyme inhibitory properties vs. derivatives featuring structural moieties that are absent in the respective MNPS. The antibacterial activity of compound **98** was explored, and it was discovered that this molecule has antibiofilm activity without mortality for marine and terrestrial bacteria [54].

**Scheme 5:**
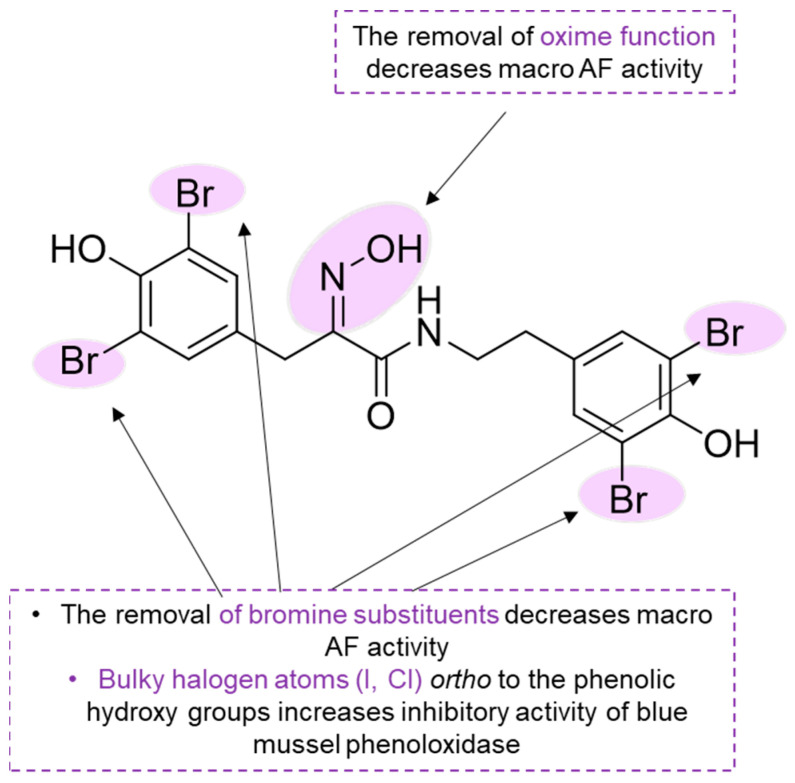
SAR for hemibastadin derivatives.

Important molecular features of hemibastadin derivatives are highlighted in Scheme 5.

Compound **98** (Figure 14) was incorporated in a biodegradable polymer poly(*ε*-caprolactone-co-*δ*-valerolactone), and demonstrated an ability to reduce the biofilm development of a mixture of bacteria and diatoms species both in situ and in vivo assays, realized during 28 and 21 days, respectively [55].

In what concerns the mechanism of action, synthetic hemibastadin derivatives were shown to modify the intracellular Ca^2+^ levels inhibiting the attachment of cyprid barnacles and the catalytic activity of blue mussel phenoloxidase, explaining their ability to disturb the settlement of this invertebrate [51,55,56].

### 2.8. Chalcones and Flavonoids

Chalcones are privileged scaffolds in medicinal chemistry, being a central core of many bioactive compounds and are biogenetic precursors of important molecules such as flavonoids. The carbonyl-conjugated system with two electrophilic centers make it susceptible to a wide range of reactions such as nuchleophilic additions and Dies-alder cycloadditions [57]. Chalcones represent one of the major subclasses of flavonoids and their importance in medicinal chemistry is undeniable [58,59]. They can be isolated from natural sources or easily obtained from simple synthetic methods. Several biological activities of natural and synthetic chalcones were reported. Moreover, they have slimicidal and anticorrosive properties, which make them ideal candidates for use in AF paints [60,61]. Since 2010, more than 70 synthetic chalcone derivatives were reported to have AF activities against micro and macrofoulers [60,62,63].

**Figure 15:**
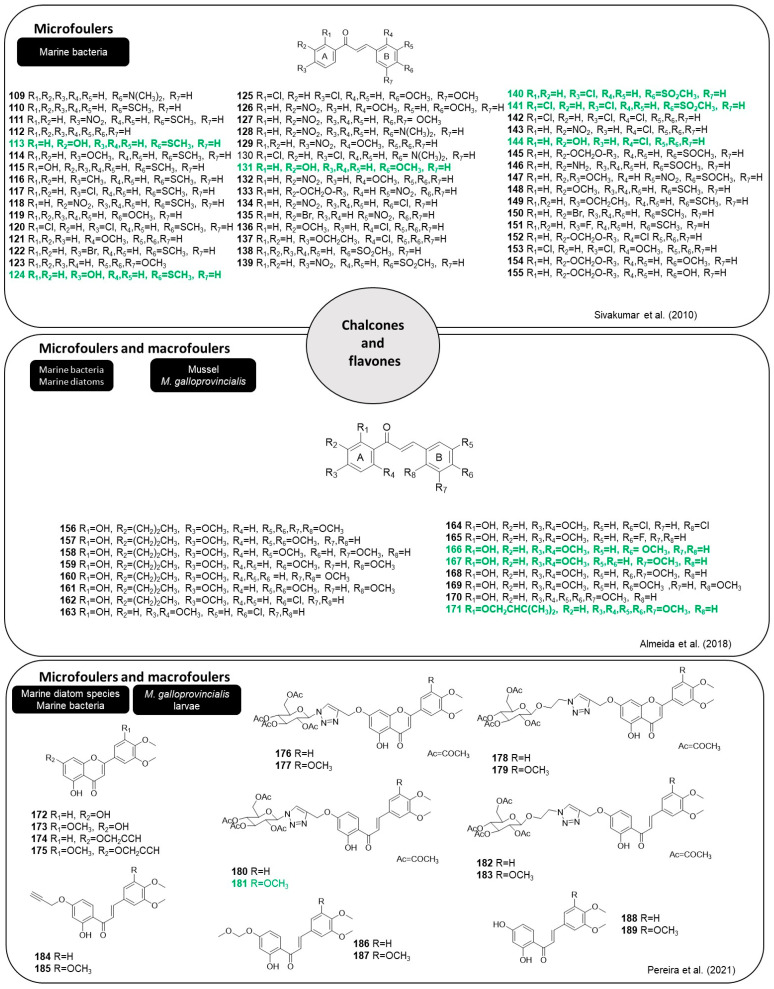
Chalcones (**109**–**171**, **180**–**189**) and flavones (**172**–**179**) [60,62,64]. Green numbers: most active compounds.

A series of chalcone derivatives was synthesized (**109**–**155**, Figure 15) and their AF activities were evaluated against three marine bacteria: *Bacillus flexus*, *Pseudomonas fluorescens*, and *Vibrio natriegens* isolated from biofilms formed on polymer and metal surfaces immersed in ocean water [60]. Regarding *B. flexus*, compounds **113**, **115**, **124**, **131**, and **144** were the most active compounds (MIC = 0.002–0.116 µM) and compounds **114**, **116**, **129**, and **134** (MIC = 0.014–0.466 µM) were the least active. Most of the compounds showed high activity against *P. fluorescens*, except compounds **109**, **148**, which were poorly active. Compounds **113**, **131**, **139**, **140**, and **144** were the most active compounds against *V. natriegens*. Overall, compounds **113**, **124**, **131**, **140**, **141**, and **144** were found to be the most promising due to the broad activity against the three marine bacteria species. Interestingly, most of them had a hydroxy pattern of substitution.

Quantitative structure–activity relationship (QSAR) studies were performed and showed the contribution of the spatial, structural, and electronic descriptors towards the biological activity. Steric factors were found to contribute negatively to the activity and consequently, small molecules were more active. Hydrophobicity was found to be positively correlated to the activity, which indicates that hydrophobic molecules have better activity. An increase in the number of free rotatable bonds in these compounds showed a negative contribution to the activity, while an increase in the relative negative charged surface area of the molecules led to an increase in the activity [60].

A series of chalcone derivatives displaying AF properties (**156**–**171**, Figure 15) was also investigated [62]. Firstly, compounds were evaluated against the adhesive larvae of the macrofouling mussel *M. galloprovincialis*. This was the first report of chalcones with anti-macrofouling activity: compounds **166**, **167**, and **171** (EC_50_ = 34.63, 7.24, and 16.48 µM, respectively) were active, with no toxicity against *M. galloprovincialis*, for the maximal concentration tested (200 µM). These compounds were then evaluated concerning their inhibitory activity against the growth of five biofilm-forming marine bacteria (*C. marina*, *V. harveyi*, *P. atlantica*, *H. aquamarina*, and *R. litoralis*) and four marine diatom strains (*Cylindrotheca* sp., *Halamphora* sp., *Nitzschia* sp., and *Navicula* sp.). Compounds **167** and **171** showed activity against *H. aquamarina* (EC_50_ = 18.67 and 18.78 µM, respectively) and *R. litoralis* (EC_50_ = 4.09 and 12.34 µM, respectively) and compound **171** also showed activity against the four diatoms strains (EC_50_ = 6.75–20.31 µM). Ecotoxicity for the most promising chalcones (**166**, **167**, and **171**) was assessed using *A. salina* standard ecotoxicity assay, and no toxicity was observed (50 µM). QSAR studies were also performed in this work and showed the contribution of geometric and relative charged surface area descriptors towards the AF activity against the settlement of mussel larvae of *M. galloprovincialis* [62].

Following the report of several sulfated compounds with AF activity (Section 2.26) [65], including glycosylated flavones, new potential AF polymethoxylated chalcone and flavone derivatives with glycosyl groups incorporating a 1,2,3-triazole moiety (**189**, Figure 15) were synthesized and their AF potential was studied, namely anti-settlement activity towards *M. galloprovincialis* larvae and antibacterial activity of five biofilm-forming marine bacteria species (*C. marina*, *V. harveyi*, *P. atlantica*, *H. aquamarina*, and *R. litoralis*) [64]. Chalcones **183**, **188**, and **189** and flavone **173** effectively inhibit the settlement of *M. galloprovincialis* larvae (EC_50_ < 25 µg·mL^−1^). Among these compounds, chalcone **183** was the most potent (EC_50_ = 3.28 µM; 2.43 µg·mL^−1^) and showed a high therapeutic ratio (LC_50_/EC_50_ > 60) [64]. The hydrogen bonding acceptor ability of the compounds, the average complementary information content of order 2, and the presence of triple bonds are the three descriptors that were found to have an impact on the AF activity displayed by this series of compounds in the QSAR model obtained. The first descriptor increased the AF activity (chalcone **183**), while the other two descriptors negatively affected the AF activity (**175**, **180**, and **181**) [64]. Regarding the inhibition of biofilm-forming marine bacteria, only chalcones **180** and **181** were able to inhibit the growth of one of the species tested, *R. litoralis* (EC_30_ = 135 and 83.5 µM). Compounds **173**, **183**, **190**, and **189** were also tested against the inhibition of the growth of *Navicula* sp., a marine diatom species, but only compound **183** showed significant activity (EC_50_ = 41.76 µM) [64]. Ecotoxicity results for the most promising compounds (**173**, **183**, **188**, and **189**) showed that these compounds were not toxic against the non-target species *A. salina* at 25 and 50 µM [64]. Overall, compound **183** was the most promising, showing both anti-macrofouling and anti-microfouling effects and no toxicity against a non-target organism.

More recently, the AF activity of six synthetic furylchalcones was verified in field tests and the results are described in the furan and furanones section (Section 2.11) [63].

### 2.9. Coumarins

Coumarins are a family of specialized metabolites found in various species of plants, fungi, and microorganisms. Coumarin was isolated for the first time by Vogel in 1820 from the plant *Dipteryx odorata*, where it comes from the origin of class designation. Coumarins are organic heterocycles with the nucleus represented by benzo-α-pyrone (2*H*-1-benzopiran-2-one). The versatility of coumarin scaffold show applications in medicinal chemistry, the agrochemical field, and also in the cosmetic and fragrances industry. This privileged scaffold is crucial for the design of compound libraries in the search for novel biological active compounds [66].

**Figure 16:**
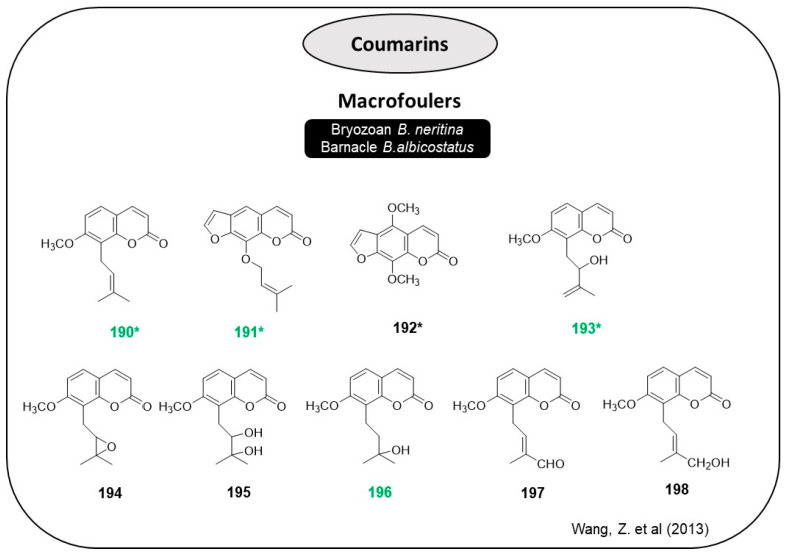
Natural and synthetic coumarins (**190**–**198**) [67]. * Natural products, green numbers: most active compounds.

Osthole (**190**) and the other three natural coumarins, imperatorin (**191**), isopimpinelin (**192**), and auraptenol (**193**) (Figure 16) were isolated from the *Cnidium monnieri* fruits. Five other coumarins with a similar skeleton were synthesized by green chemistry procedures from osthole and AF activity against *Balanus albicostatus* and *Bugula neritina* was evaluated for all the compounds to comprise the relationship between the functional groups and AF activity [67].

Compounds **190**, **191**, **193**, and **196** showed high inhibitory activities against *B. albicostatus* with EC_50_ values < 5 μg·mL^−1^. All compounds (Figure 16) except for **191**, **192**, and **194** showed inhibitory activities against bryozoan *B. neritina* settlement with EC_50_ values < 25 μg·mL^−1^, and the EC_50_ value of compound **197** was lower than 5 μg·mL^−1^ [67]. Compounds **190**, **197**, and **198** have the same coumarin structures, except that C-5′ groups were -CH_3_ (**190**), -CHO (**197**), and -CH_2_OH (**198**), respectively. For these compounds, EC_50_ values against *B. albicostatus* were 19.01 μmol·mL^−1^ (**190**), 26.24 μmol·mL^−1^ (**197**), and 42.04 μmol·mL^−1^ (**198**), and EC _50_ values against *B. neritina* were 15.00 μmol·mL^−1^ (**197**) 30.98 μmol mL^−1^ (**190**) and 47.62 μmol mL^−1^ (**198**) [67]. Compounds **190**, **194**, **195**, and **196** have the same structures, except that the groups between C-2′ and C-3′ have a double bond (**190**), epoxy (**194**), dihydroxy (**195**), and hydroxy (**196**), respectively. The EC_50_ values against *B. albicostatus* were 17.17 μmol·mL^−1^ (**196**), 19.01 μmol·mL^−1^ (**190**), 28.69 μmol·mL^−1^ (**194**), and 127.19 μmol·mL^−1^ (**195**). The EC_50_ values against *B. neritina* were 30.98 μmol·mL^−1^ (**190**), 33.31 μmol·mL^−1^ (**196**), 65.58 μmol·mL^−1^ (**195**), and 143.42 μmol·mL^−1^ (**194**) [67]. Osthole (**190**) and furanocoumarin (**191**) have the same isoamylene chain. The EC_50_ values against *B. albicostatus* are similar for both compounds (compound **190**, 19.01 μmol·mL^−1^ and compound **191**, 12.56 μmol·mL^−1^). The furan ring appears to influence AF activity against *B. neritina* (compound **190**, 19.01 μmol·mL^−1^ and compound **191**, >185.19 μmol·mL^−1^). In this point of view, the results reveal that groups on C-5′ and C-2′/C-3′ of the isoamylene chain could affect the AF activities [67]. Concerning the toxicological profile, the therapeutic ratio of compounds **190**, **194**, **196**, and **197** was higher than 5.0, indicating that these compounds have low toxicity against *B. albicostatus* larvae. All compounds showed no relevant mortality effect on *B. neritina* at a concentration of 50 μg·mL^−1^ [67]. Regarding the mechanism of action, it deserves to be highlighted that the coumarin ring shows antibacterial activity due to the ability to inhibit bacterial nucleic acid synthesis and quorum sensing system, which decreases the formation of biofilms on surfaces [68].

### 2.10. Diterpene Derivatives

**Figure 17:**
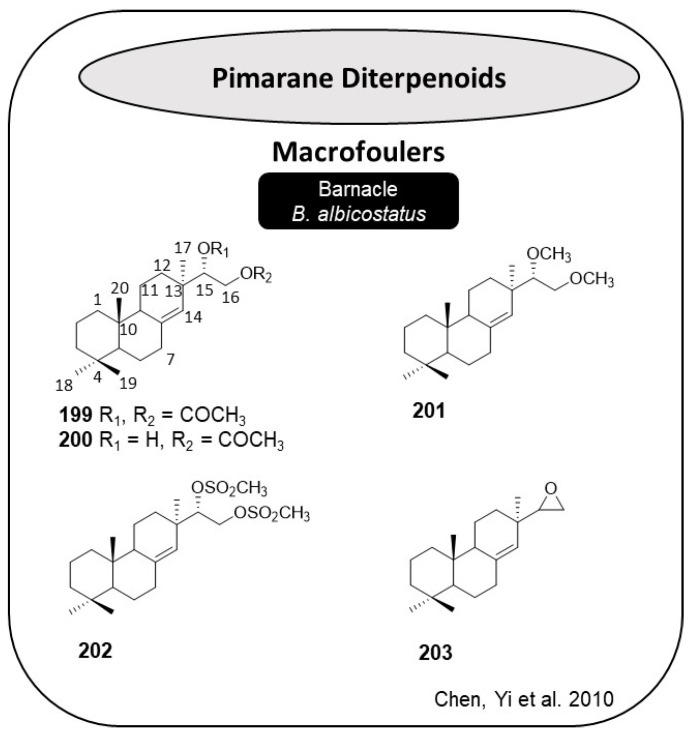
Synthetic pimarane diterpenoids **199**–**203** [73].

**Scheme 6:**
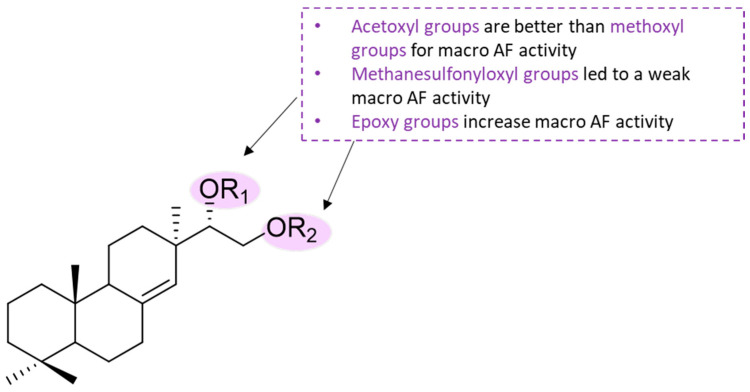
SAR for pimarane diterpenoids.

Diterpenes are a class of natural products originating from C20 precursor geranylgeranyl diphosphate [69]. Pimarane diterpenes are a kind of tricyclic diterpene and could be isolated mainly from plants and fungi [70]. Due to the stereochemistry diversity, pimarane diterpenes are distinguished into pimarane, isopimarane, and *ent*-pimarane. This group of compounds became a direction in the research of novel active compounds because of its wide applications in medicine and agriculture [71]. During the investigations on environmentally friendly AF compounds, it was observed that pimarane diterpenoids can exhibit non-toxic AF activities against the larval settlement of the barnacle *B. albicostatus* [72]. To investigate the SAR of these pimarane diterpenoids, they synthesized five new pimarane diterpenoids (**199**–**203**, Figure 17) and tested their ability to inhibit the settlement of the larvae of barnacle *B. albicostatus* [73]. It was observed that substitution patterns at C-15 and C-16 positions of pimarane diterpenoid significantly affected the AF activity. For example, substituents, such as the acetoxyl, methoxyl, or methanesulfonyloxyl group at C-15 and C-16 positions of the pimarane diterpenoid led to less potent compounds. Additionally, the AF activity of acyl compounds **199** and **200** (EC_50_ = 0.30 and 0.14 µg·cm^−2^, respectively) was higher than for the methylated compound **201** (EC_50_ = 0.57 µg·cm^−2^), which suggests that an acetoxyl group at a suitable position of the pimarane diterpenoid scaffold might result in increased AF activity compared to the methoxyl group. The methanesulfonyloxy group showed no significant impact on the AF activity of pimarane diterpenoids, as compound **202** showed weak AF activity (EC_50_ = 7.47 µg·cm^−2^) [73]. Additionally, an epoxy group replacing hydroxy groups at the C-15 and C-16 positions in pimarane diterpenoid greatly increased AF activity as seen by compound **203** (EC_50_ = 0.05 µg·cm^−2^), which suggests that the epoxy group might be an important functional group for potent AF activity in pimarane diterpenoids [73]. No toxicity was observed for all the compounds (LC_50_ > 10 µg·cm^−2^), indicating that the substituents in the side chain of pimarane diterpenoids did not influence the toxicity [73]. Some insights of the SAR for pimarane diterpenes are evidenced in Scheme 6.

**Figure 18:**
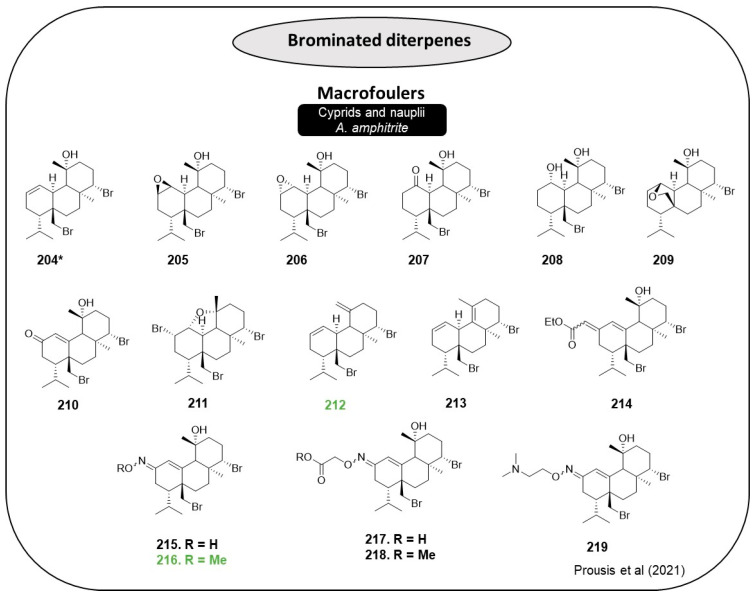
Bromosphaerol (**204**) and its synthetic derivatives **205**–**219** [76]. * Natural compounds; green numbers: most active compounds.

Bromosphaerol (**204**, Figure 18) is a brominated diterpene isolated from the red alga *Sphaerococcus coronopifolius* [74,75]. This molecule showed robust anti-settlement activity against larvae of *A. amphitrite* with very low toxicity. From this perspective, 15 structural analogs involving transformations at ∆^1^ double bond and positions C-11, C-16, and C-17 (**205**–**219**, Figure 18) were designed and synthesized to improve bromosphaerol AF activity [76]. Using cyprids and nauplii of *A. amphitrite* as a model organism, the anti-settlement activity (EC_50_) and the degree of toxicity (LC_50_) of the bromosphaerol derivatives were evaluated. The design thinking to achieve SAR embraced introducing polar groups at C-1 and/or C-2 (**205**–**209** and **211**), removing the C-11 hydroxyl group (**212** and **213**) and substituting C-2 with functional groups, in specific, an ester and an oxime, while the ∆^1^ double was repositioned to C-1-C-10, providing the generation of an extended conjugated system (**210** and **214**–**219**) [76].

Analogs **212**, **216**, **218**, and **219** revealed promising AF efficacy (EC_50_ < 0.5 mg·L^−1^). Compounds that bear an oxygen moiety at C-1 and C-2 (**205**, **207**, **209**, and **210**) exhibited similar EC_50_ values ranging from 10.44 to 8.75 mg·L^−1^. The introduction of bromine at C-2 abolished the activity (compound **211**). α,β-unsaturated ester analog **214** did not reveal a promising AF compound (EC_50_ > 50 mg·L^−1^). The elimination of the hydroxyl group at C-11 to form the exocyclic double bound showed robust AF activity with na EC_50_ < 0.5 mg·L^−1^ (**212**). The exocyclic elimination congener was less potent (EC_50_ = 3.87 mg·L^−1^). It was possible to conclude that the nature of oxime functionality impacts AF activity. Supporting that, the unsubstituted oxime compound **215** and the carboxy oxime were revealed to be inactive, contrasting with the methoxy oxime analog **216** and the methyl ester congener of **217**, analog **218**, which presents EC_50_ < 0.5 mg·L^−1^. In the same point of view, the dimethylaminoethyl oxime derivative **219** showed EC_50_ < 0.5 mg·L^−1^ [76].

**Scheme 7:**
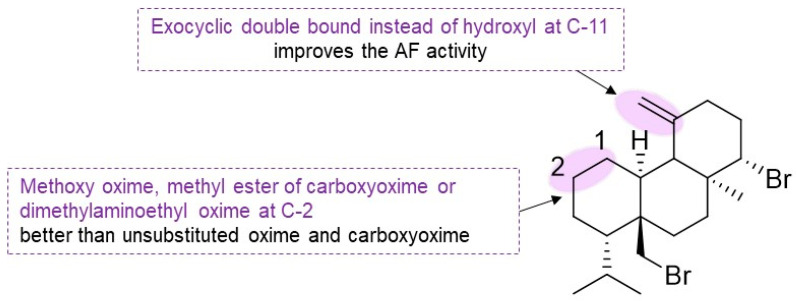
SAR for brominated diterpenes.

Concerning the toxicity assessment, structural analogs **205**, **207**, and **209**–**219** showed diverse levels of AF activity. While the LC_50_ values for derivatives **212** and **216** were >50 mg·L^−1^, compounds **218** and **219** revealed high toxicity towards the barnacle naupliar stage with LC_50_ values of 2.7 and 12.5 mg·L^−1^, respectively. Epoxy derivative **205** and the tetrahydrofuranyl analog **209** possess LC_50_ values of 25.2 and 10.2 mg·L^−1^, respectively. All the other compounds were not toxic against cypris larvae with LC_50_ values > 50 mg·L^−1^. Compounds **212** and **216** were demonstrated as well-performing antifoulants, acting through a non-toxic mechanism with therapeutic ratio values > 100 on *A. amphitrite* cypris [76]. Some insights of the SAR for brominated diterpenes are evidenced in Scheme 7.

### 2.11. Furan and Furoates

**Figure 19:**
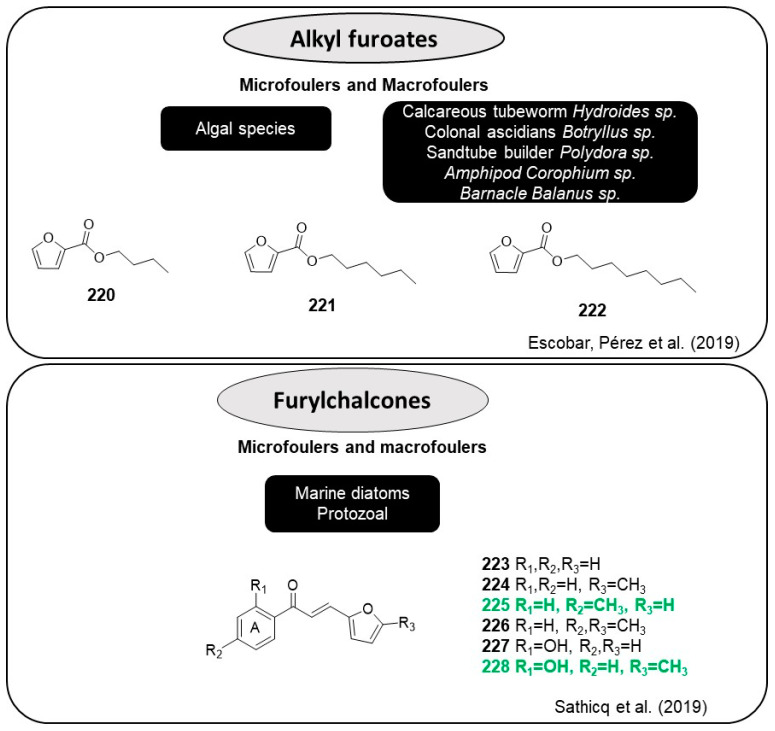
Alkyl furoates (**220**–**222**) and furylchalcones (**223**–**228**) [63,78]. Green numbers: most active compounds.

Studies on SAR demonstrated that the presence of furan group displays a crucial role in antifouling activity [77]. In this way, alkyl furoates are considered potential antifouling agents due to two features: the presence of furan ring and the possibility of its total synthesis from a lignocellulosic biomass, which is congruent with green chemistry concepts. These compounds are biodegradable, which makes them promising for application in the marine environment. In this direction, three *N*-alkyl 2-furoates (**220**–**222**, Figure 19) were synthesized by green chemistry methods [78]. To evaluate the possibility of using alkyl furoates as AF agents, the selected model organism was *Artemia* sp. nauplii, which is a valuable tool for the study of larval toxicity. Remarkably, the compounds impacted larval activity at low concentrations and the toxicity of the studied compounds increased as the carbon chain length increased. Moreover, the alkyl-furoates were included in rosin-based AF paints. After 45-day exposure in the sea, all three paints formulated with furoates showed a strong AF activity compared to the control (paint without alkyl-furoates). Furoate paints completely inhibited the settlement of conspicuous species of Mar del Plata harbor such as the calcareous tubeworm *Hydroides* sp., colonial ascidians such as *Botryllus* sp., and the sandtube builder *Polydora* sp. It is important to note that compounds **220** and **221** demonstrated inhibition of the settlement of the amphipod *Corophium* sp., algal species such as *Enteromorpha* sp. and *Griffithsia* sp., and they reduced *Ectocarpus* sp. Abundance, presenting significantly lower cover percentages compared to compound **222**. Remarkably, compound **220** prevented the settlement of *Amphibalanus* sp. [78]. According to these results, alkyl furoates were pointed out as promising sustainable AF compounds that could replace the use of metallic AF pigments in marine paints.

More recently, field tests were performed for six synthetic furylchalcones (**223**–**228**, Figure 19), which were incorporated in rosin-based AF paints, and the fouling cover percentage on painted panels submerged in the sea for 45 days was estimated [63]. All furylchalcones **223**–**228** showed AF activity and a SAR study was performed. The replacement of the B ring by a furan ring resulted in an increase in AF activity against macrofouling organisms (**223**–**228**). The presence of a methyl group in the furyl ring (**224**, **226**, and **228**) decreased antimacrofouling, anti-diatom, and antiprotozoal activities compared to compounds with no methyl groups (**223**, **225**, and **227**). Compounds **227** and **228**, with a hydroxy group at position 2′ of ring A were more effective than compounds **223** and **224** in the inhibition of macrofoulers [63]. The authors highlighted that furylchalcones can be synthesized from building blocks present in vegetal biomass by simple and high yield procedures, rendering them good candidates for the development of AF compounds. Considering all chalcone derivatives, anti-macrofouling activity was associated with the presence of a hydroxy group in the A-ring and a furyl in the B ring (Figure 15, Section 2.8) of the chalcone scaffold. Hydrophobicity of the molecules was associated with a favorable AF activity [63].

### 2.12. Indole Derivatives

The indole heterocycle is found in natural products. Indoles show prominent biological activities including anticancer, antioxidant, anti-inflammatory, antifungal, anticholinesterase, and antibacterial properties [79,80]. Isatin (**229**), an antifungal and ecologically defensive marine natural product isolated from marine bacterium isolated from healthy embryos of the caridean shrimp Palaemon macrodectylus (*altermonas* sp.), was proposed as an alternative molecular AF scaffold and as a source for structural insight to guide the molecular design of AF compounds [81]. Nearly 30 natural and synthetic indole derivatives were reported in the literature as follows.

**Figure 20:**
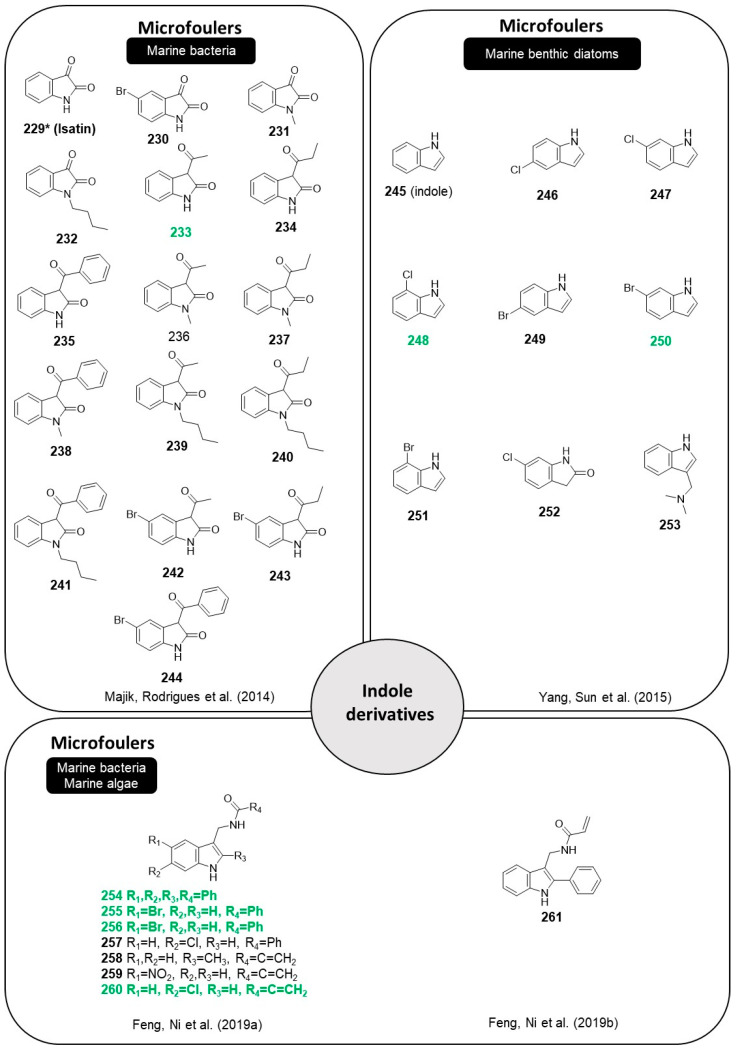
Indole derivatives **229**–**261** [81,82,83]. * Natural products; green numbers: most active compounds.

Derivatives of isatin (**230**–**244**, Figure 20) were obtained to understand the structural requirements for AF activity and tested against the growth of bacteria isolated from natural biofilms [81].

**Scheme 8:**
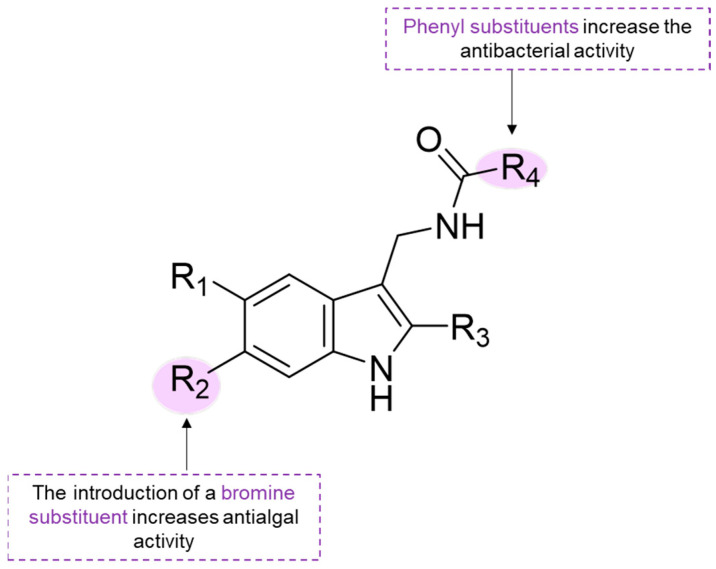
SAR for indole derivatives.

Briefly, the use of bromine substituent at the C-5 carbon atom in the derivatives of isatin led to a decrease in antibacterial activity as compared with isatin (**229**). A free NH is necessary for good inhibitory activity of isatin (**229**) against fouling bacteria, as *N*-methyl and *N*-butyl isatin derivatives showed decreased activity. The presence of the 3-acetonylidene group and a free NH moiety increased antibacterial activity as observed for compound **233**, which was the most potent compound. The effects of synthetic indole derivatives (**245**–**253**, Figure 20) were evaluated against two marine benthic diatoms (*Nitzschia closterium f. minutissima* and *Navicula climacospheniae*) [82]. Indole derivatives containing a simple indole ring and halogenated substituents significantly inhibited the growth of the two types of diatoms. In general, the presence of a halogen increased anti-algal activity, but it was dependent on the position of the halogen in the indole ring. Indole derivatives with chlorine at position 7 (**248**) showed increased activity compared with derivatives with chlorine at positions 5 and 6 (**246** and **247**). On the other hand, bromine at position 6 (**250**) was more favorable for antialgal activity compared with positions 5 and 7 (**251** and **252**). Diatom species determined the sensitivity of various indole derivatives. Derivatives with chlorine or bromide (**246**–**252**) were more potent against *N. closterium f. minutissima* than against *N. climacospheniae*. Recently, indole derivatives were tested in a marine environment after incorporating them in marine AF coatings (**254**–**260**, Figure 20), after performing antibacterial and anti-algal experiments [83]. The antibacterial activity of indole derivative **256** was significantly higher than those of indole derivative **254**, which suggests that phenyl substituents increase the antibacterial activity. Indole derivative substituted with a bromine group (**255**) showed slightly higher antibacterial activity compared to indole derivative **254**. Substituents of the indole ring resulted in great differences in algal inhibition. An acrylamide methylene group (**260**) was more favorable than a benzamide methylene group (**257**) in the inhibition of *N. closterium* and *Platymonas subcordiformis*. A bromine atom seems to increase anti-algal activity, as observed for the activity compound **255** against *N. closterium* when compared to compound **254**, and the activity of compound **255** against three algae species when compared to compound **257** [83]. Some insights of SAR for indole derivatives are evidenced in Scheme 8. Compound **254** was selected for the net Ca^2+^ flux test due to the higher inhibition rate of *P. subcordiformis* and the results might indicate that indole derivatives can act as inhibitors of transmembrane transport and trigger algal cellular Ca^2+^ efflux. AF coatings containing indole derivatives **254**–**260** were prepared and tested in a marine environment (Qingdao Harbor) for five months. Polyvinyl chloride (PVC) panels brushed with coating containing Cu_2_O and the indole derivatives (compounds **254**–**256** and **260**) were adhered by fewer fouling organisms, which is consistent with the results obtained in the laboratory experiments [83].

A novel indole derivative, compound **261** (Figure 20), with an acrylamide group and structurally similar to compounds **254**–**260**, was obtained by synthesis, and its AF properties were studied concerning their ability to inhibit the growth of bacteria (*Escherichia coli* and *Staphylococcus aureus*) and marine algae (*Phaeodactylum tricornutum*, *N. closterium*, and *Skeletonema costatum*) [84]. Results show that compound **261** has a bacteriostasis rate of 94.91% and 94.91% against *E. coli* and *S. aureus*, respectively. The inhibition rates of compound **261** against marine algae were between 92% and 95%, showing also evident anti-algal properties. Further studies regarding the performance of compound **261** immobilized in an acrylate resin were performed. Inhibition rates of acrylate resin containing compound **261** against *P. tricornutum*, *N. closterium* were improved by one-fold, compared with acrylate resins alone, in static conditions. In dynamic conditions, acrylate resins with compound **261** were immersed in the sea for three months and distinct differences between different contents of indole derivative **261** were observed, as the control acrylate resin was completely covered by algae. In contrast, resins containing compound **261** showed less fouling and the observed fouling was directly proportional to the indole content in the resin [84]. Overall, compound **261** displayed antimicrobial and antialgal properties, even after being incorporated in acrylate resin, highlighting its potential to be used in marine paints and have a real application [84]. These results are the proof of concept that these indole derivatives have AF properties and can be further studied to be used as AF ingredients in marine paints. Scheme 8 summarizes the moyeties important for AF activity based on the described studies.

### 2.13. Isocyanide-Containing Compounds

The first isocyanide was synthesized in 1859 and the first natural isocyanide was identified in 1950. The isocyanide concerns an interesting functional group in organic chemistry, as its carbon can act as (i) a nucleophile attacking activated electrophiles, (ii) as an electrophile being intercepted by different nucleophiles, (iii) as a carbene involved in formal [4 + 1] cycloaddition, and (iv) as a radical acceptor to form imidoyl radical reaction intermediates. Additionally, lone pair interactions enable the preparation of various coordination complexes [85]. Isocyanides-containing molecules reveal a powerful diversity of biological activities.

Although compounds containing an isocyano group are a rare sort of MNPs, some were isolated from certain marine sponges [86]. Most natural isocyanides are toxic toward fish and crustaceans, which indicates a role in chemical defense and perhaps a role as repellants of epibiotic organisms [86]. Natural isocyanides have complex structures and the synthesis of simpler analogs with feasible chemical reactions seem to be a suitable solution to obtain these compounds in higher quantities. Isocyanides were the most explored compounds as AF agents, with 110 synthetic compounds reported from 2002 to 2017.

**Figure 21:**
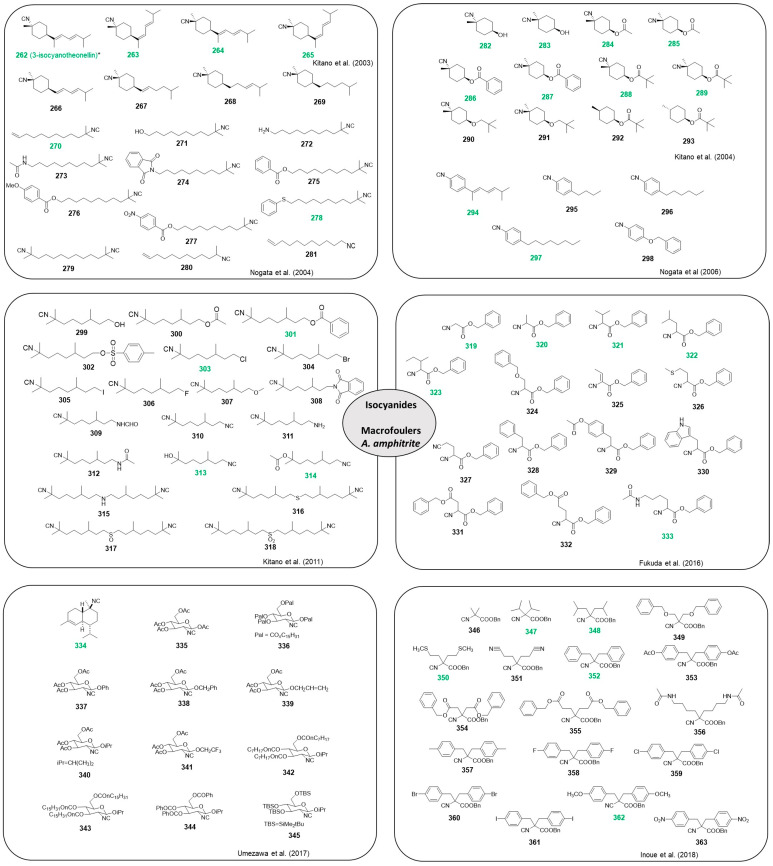
3-Isocyanotheonellin (**262**) and synthetic isocyanide-containing compounds **263**–**363**. * [87,88,89,90,91,92,93,94]. Natural products; green numbers: most active compounds.

3-Isocyanotheonellin (compound **262**, Figure 21), a marine sesquiterpene from a nudibranch species, with an isocyano group at the C-3 position, exhibited potent AF activity against the larvae of the barnacle *A. amphitrite* (EC_50_ = 0.13 μg·mL^−1^). The SAR for compound **262** analog was first explored through the synthesis of seven analogs (compounds **263**–**269**, Figure 21) and the AF activity evaluation against the settlement of *A. amphitrite* larvae [87,88]. Modifications were based on the stereochemistry of the isocyano group and the double bonds. The stereochemistry of the double bond did not significantly influence the AF activity, as compound **263** (EC_50_ = 0.29 µg·mL^−1^) only displayed a slight activity difference compared to compound **262** (EC_50_ = 0.19 µg·mL^−1^) [87]. Concerning the stereochemistry of the isocyano group, the AF performance of compounds **268** and **269** (EC_50_ = 0.18 and 0.41 µg·mL^−1^) was not significantly different from the performance of compounds **262** and **263** [88]. The LC_50_ of cyprids was calculated for all the compounds (**262**–**269**, Figure 21), and all of them were not toxic at high concentrations to the cyprid larvae (LC_50_ > 100 µg·mL^−1^) [88].

**Scheme 9:**
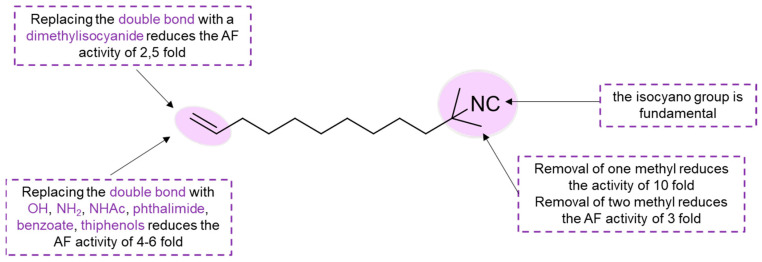
SAR for isocyanide **270**.

Another detailed SAR for compound **262** was attempted with the synthesis of four analogs (**266**–**269**, Figure 21) [88]. Analogs **266**–**269** were less potent than analogs **263**–**265**, showing only moderate AF activity (EC_50_ = 1.80–7.20 µg·mL^−1^). The methyl group at C-14 seems to contribute to the potent AF activity displayed by compounds **263**–**265**, probably by influencing the conformation of the molecules [88]. This work was the inspiration for the synthesis of twelve simple linear isocyanides (**270**–**281**, Figure 21) [89]. All the compounds displayed AF activity, suggesting that the isocyano group is important for the AF activity. Compound **270**, together with phenylthiol **278**, showed the most potent AF activity (EC_50_ = 0.046 and 0.056 µg·mL^−1^, respectively) without toxicity to the cyprid larvae (LD_50_ > 30 µg·mL^−1^). Compounds **270**, **280**, and **281**, which differ only in the number of methyl groups in the isocyano moiety, showed differences in AF activity, in which compound **270** with two methyl groups was the most active and compound **281** with no methyl groups was the less active (EC_50_ = 0.14 µg·mL^−1^). The differences in the AF activity in these compounds could indicate that the lipophilicity near the isocyano group influences AF activity [89]. The most hydrophobic compounds (**275**–**277**) showed only moderate AF activity, which might be associated with their poor solubility. Compounds **271**–**273**, possessing a hydrogen bonding donor group, showed some toxicity against the cyprid larvae (LD_50_ = 21.28–22.25 µg·mL^−1^), which puts forward the possible association of the hydrogen bonding donor with toxicity [89]. Scheme 9 summarizes the SAR based on compound **270** [85].

Studies concerning the mechanism of action of compound **270** in two target organisms, the bryozoan *B. neritina* and the barnacle *A. amphitrite*, and one non-target organism, the species zebrafish *Danio rerio*, were performed [95]. Compound **270** bound to mitochondrial proteins both in *B. neritina* and *A. amphitrite*, suggesting that it may influence mitochondrial functions, which can lead to changes in behaviors, such as the selection of an attachment site and/or inhibition of attachment and metamorphosis [95]. Specifically, compound **270** interacts with three proteins of *B. neritina*, in which two are identical to voltage dependent anion channels (VDAC), located on the outer part of the mitochondrial membrane involved in cellular mechanisms, such as cell metabolism and cell survival. In *A. amphitrite*, compound **270** targeted a cytochrome P450 complex enzyme, similar to a NADH-ubiquinone oxidoreductase-like protein, located in the inner part of the mitochondrial membrane, involved in the oxidative phosphorylation, where it catalyzes the electron transfer from NADH to coenzyme Q. Compound **270** in the zebrafrish embryo caused a typical signature due to copper deficiency (e.g., pericardial edema, poor blood circulation, pigmentation defects, and defect on hematopoiesis) [95].

Previous results suggest that an isocyano group and a hydrophobic site at a suitable position are important in the expression of potent AF activity [87,88]. To cover more, novel isocyano cyclohexane derivatives (**282**–**293**, Figure 21) with an ester or an ether functional group were synthesized [90]. The results obtained with compounds **282**–**285** indicate that the ester group might be important for the expression of potent AF activity against the cyprid larvae of *A. amphitrite* (EC_50_ = 0.0096–0.98 μg·mL^−1^). Following this, other ester derivatives were obtained, and a very potent AF activity was observed for compounds **286**–**289** (EC_50_ = 0.048–0.54 μg·mL^−1^). To explore the role of the ester group in detail, the synthesis of ether compounds lacking the carbonyl function was undertaken (compounds **290** and **291**), and their AF activity showed that the carbonyl moiety of the ester group was more important than the oxygen–carbon moiety (**290**: EC_50_ = 0.176 μg·mL^−1^ and **291**: EC_50_ = 17.0 μg·mL^−1^). The synthesis of compounds lacking the isocyano group was performed to verify the role of this functional group in this series of compounds. Compounds **292** and **293** did not show a significant settlement inhibition activity (EC_50_ > 30 μg·mL^−1^ for both compounds), which suggests that the isocyano group is essential for the expression of potent AF activity. On the other hand, the results of this study do not make clear the difference in AF activity that exists between the stereoisomers. For all the synthesized compounds, no significant mortality at high concentrations was observed [90].

Although isocyano cyclohexanes showed promising AF activity without showing toxicity, the yields on the synthesis of these compounds were poor. Therefore, simpler isocyano benzenes were prepared (**294**–**298**, Figure 21) [91]. Compound **294**, a phenyl version of 3-isocyanotheonellin (**262**), was found to be potent against the settlement of *A. amphitrite*, without significant toxicity (EC_50_ = 0.078 μg·mL^−1^; LD_50_ > 100 µg·mL^−1^). The increase in chain length led to an increase in AF activity, as observed for compounds **295**–**297**, but only compound **297** did not show significant toxicity (LD_50_ > 3.0 µg·mL^−1^). Compound **298** was the most potent compound; however, it was very toxic against the larvae of *A. amphitrite* (EC_50_ = 0.054 µg·mL^−1^; LC_50_ = 3.0 µg·mL^−1^). Previous SAR studies indicated that an isocyanide with a tertiary alkyl isocyano group was more active than the congeners containing primary or specialized alkyl isocyano groups [91]. Twenty novel simple isocyanides were synthesized (**299**–**318**, Figure 21) containing a tertiary alkyl isocyano group derived from citronellol without using a Grignard reaction [96]. All the isocyano compounds exhibited potent AF activity against cypris larvae of the barnacle *A. amphitrite*, without significant toxicity. Benzoate **301** and chloride **303** were the most potent AF compounds in this study and did not show significant toxicity (EC_50_ = 0.08 μg·mL^−1^ and LC_50_ > 100 µg·mL^−1^ for both compounds). Both compounds with a tertiary and with a primary alkyl (compounds **313** and **314**) isocyanide showed potent AF activity (EC_50_ < 1.0 µg·mL^−1^). Compounds **299**–**302** (EC_50_ = 0.08–0.38 µg·mL^−1^) and **307** (EC_50_ = 0.32 µg·mL^−1^) with hydroxy, ether, ester, or the sulfonate ester functional group, and halides **303**–**306** (EC_50_ = 0.08–0.057 µg·mL^−1^) showed good AF activity. Additionally, compounds **308**, **309**, **311**, and **312**, which possess an imide, amide, or amine group, also exhibited good AF activity. Diisocyano sulfide **316** and diisocyano sulfone **317** showed only moderate AF activity (EC_50_ = 1.49 and 1.32 µg·mL^−1^, respectively), suggesting that an increase in the number of isocyano groups does not translate to an increase in the AF activity [96]. The results clearly show that in comparison with a linear alkyl chain, the isoprene framework derived from citronellol was a better spacer to achieve AF activity without toxicity.

Isocyanides derived from amino acids as suitable candidates to be environmentally friendly AF agents were also explored [92]. Some factors contribute to the suitability of isocyanides derived from amino acids, specifically, (i) amino acids can be converted to the corresponding isocyanides (amino acid-isocyanides) in only a few reactional steps; (ii) amino acid-isocyanides are expected to show potent AF activity without significant toxicity due to the presence of the isocyano group, and (iii) are expected to biodegrade to the original non-toxic amino acids. All the 15 synthesized isocyano compounds (**319**–**333**, Figure 21) showed effective AF activity against cyprid larvae of *A. amphitrite*. Isocyanides **319**–**323** (EC_50_ = 0.97–2.67 µg·mL^−1^) and **333** (EC_50_ = 2.63 µg·mL^−1^) exhibited relatively potent AF activity, whereas isocyanides **324** (EC_50_ = 7.99 µg·mL^−1^), **326** (EC_50_ = 3.97 µg·mL^−1^), **327** (EC_50_ = 10.34 µg·mL^−1^), **331** (EC_50_ = 5.49 µg·mL^−1^), and **332** (EC_50_ = 3.96 µg·mL^−1^) showed moderate AF activity, which indicates that the aromatic ring of the α-side chain might be effective in improving AF activity [92]. Secondary alkyl isocyanides derived from α-amino acids had potent AF activity, as well as the tertiary alkyl isocyanides [92]. All the synthesized amino acid-isocyanides showed low mortality rates at high concentrations, after 120 h of exposure [92].

Umezawa et al. performed the total synthesis of 10-isocyano-4-cadinene (**334**, Figure 21), a MNP obtained from nudibranchs of the family *Phyllidiidae* [93]. The synthetic intermediates without the isocyanate group were also investigated and showed weak AF activity against cypris larvae of the barnacle *A. amphitrite* [93]. After these results, glucosamine-based isocyanides (**335**–**345**, Figure 21) were synthesized and the effect of substituents at C-1, C-3, C-4, and C-6 positions on the AF activity was evaluated [93]. Compounds with an ether group at C-1 (compounds **337**–**341**; EC_50_ = 0.23–0.71 µg·mL^−1^) were more active than compounds with an ester group (compounds **335** and **336**; EC_50_ = 5.54 and 6.36 µg·mL^−1^). Changes at C-3, 4, and 6-positions (compounds **342**–**345**; EC_50_ = 0.25–0.81 µg·mL^−1^) did not significantly affect the AF activity [93].

Although almost all the amino acid isocyanides described before (**319**–**333**) effectively inhibited the settlement of barnacle larvae without exhibiting significant toxicity, they were obtained as mixtures of enantiomers. To circumvent this problem, the structure of amino acid isocyanides was modified by introducing an identical side chain at the α-position to afford achiral α,α-disubstituted amino acid isocyanides (**346**–**363**, Figure 21) [94]. Overall, the most potent AF compounds in this series were hydrophobic amino acid-derived isocyanides **347** (EC_50_ = 0.07 µg·mL^−1^), **348** (EC_50_ = 0.30 µg·mL^−1^), **350** (EC_50_ = 0.07 µg·mL^−1^), and **352** (EC_50_ = 0.14 µg·mL^−1^), which suggested that hydrophobicity might be associated with AF activity. The AF activity of compounds **347**–**352** was higher than the AF activity displayed by the corresponding monosubstituted amino acid isocyanides, suggesting that the introduction of the second side chain increases AF activity [94]. Additionally, the mortalities observed for all disubstituted amino acid isocyanides were lower than those of monosubstituted ones, indicating that the introduction of the second side chain also effectively reduces toxicity, as observed in a previous work of the same authors [89]. Previously, the authors showed that aromatic amino acid (Phe and Tyr)-derived isocyanides exhibited high AF activity (EC_50_ = 0.14 and 0.17 µg·mL^−1^, respectively) and compound **352** (EC_50_ = 0.14 µg·mL^−1^) also showed potent AF activity [92]. Therefore, the presence of aromatic rings in the α-side chain was suggested to effectively improve AF activity, inspiring the authors to prepare isocyanides **357**–**363** (Figure 21) [94]. However, these aromatic compounds only showed moderate to low AF activity except for compound **362** (EC_50_ = 0.32 µg·mL^−1^), which was attributed to sterically hindrance effects [94]. The safety of the synthesized AF-active compounds was evaluated based on their therapeutic ratios, and notably, isocyanides **347**, **348**, **350**, and **352**, which showed high AF activity, also exhibited high therapeutic ratios [94].

From the results obtained from all the isocyanide derivatives, it was possible to conclude that the presence of the isocyano group was crucial to the potent AF activity of these compounds. Additionally, lipophilicity near the cyano group was found to increase AF activity, although overall hydrophobicity was associated only with moderate AF activity due to poor solubility. Another conclusion for isocyano compounds reported was that the increase in the number of isocyano groups did not translate into an increase in AF activity. All isocyanide derivatives were tested against the settlement of the cyprid larvae of *A. amphitrite*, a macrofouling organism, although it would be interesting to test this class of compounds against microfouling species, such as marine bacteria or diatoms.

### 2.14. Lactones

Butenolides, or butyrolactones, are isolated from fungi, especially the *Aspergillus* sp., and possess the skeleton of α,β-unsaturated *γ*-butyrolactone scaffold [97]. These molecules were demonstrated to reveal distinct biological activities, including anti-inflammatory, cytotoxic, antiviral, antioxidant, antimicrobial, antidiabetic, protein kinase-inhibitory, and α-glucosidase inhibitory activities [98]. Proteomic profiling studies revealed that butenolide can disrupt the neurotransmission in the nervous system by disorganizing the cytoskeletal structure in the brains of medaka *Oryzias melastigm*, a fish model species, specifically valuable due to the relevance of ecosystem risk assessment of marine pollution [99]. Nevertheless, butanolide also induces the detoxification system in their livers, ensuring lower non-target toxicity and higher biosafety [100]. From 2010 to 2014, 61 natural butenolides and derivatives were reported in the literature.

**Figure 22:**
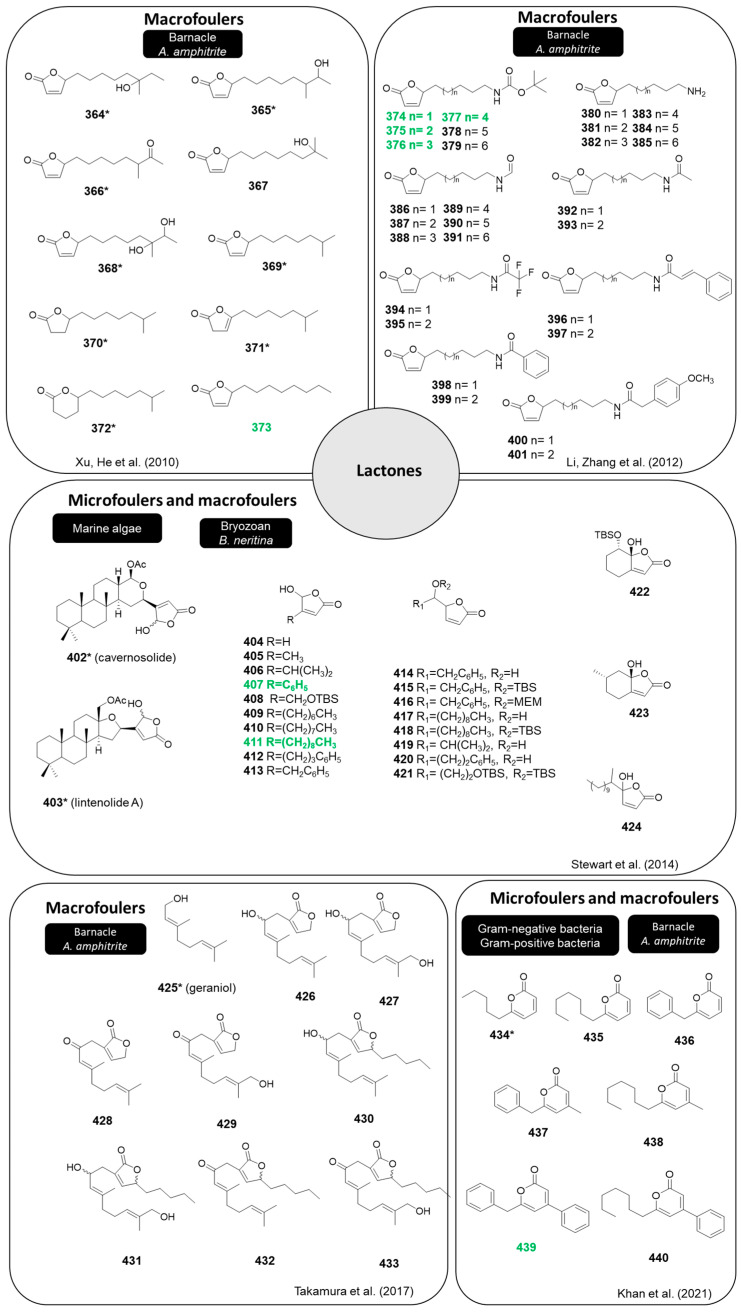
Lactones **364**–**437** [77,108,109,110,111]. * Natural products; green numbers: most active compounds.

The AF activity against the cyprids of the barnacle *A. amphitrite* of nine NPs (**364**–**372**, Figure 22) isolated from different marine *Streptomyces* sp. was investigated [77]. Compounds **367** and **368** were found to be less potent than compounds **364**, **365**, and **366**, which suggested that the side chain may affect AF activity. Compound **369** is similar to compound **367** except that its side chain does not contain a hydroxy group and is around 20 times more potent, supporting the hypothesis that lipophilicity increases AF activity. The presence of a 2-furanone ring is also essential for AF activity since compounds **370**, **371**, and **372**, which do not possess this structural feature, did not present AF activity. Based on this information, the authors synthesized a simplified molecule, compound **373** (Figure 22), containing a 2-furanone ring and a linear carbon chain. This optimized compound presented excellent AF activity (EC_50_ = 0.518 µg·mL^−1^) and did not exhibit any toxicity against barnacle larvae [77]. The AF activity of butenolide **373** was evaluated also against the polychaete *Hydroids elegans* and the bryozoan *B. neritina*. Again, a potent AF activity was observed, as well as low toxicity against these fouling organisms [77]. To investigate the mechanism of the action of compound **373**, an analysis of the proteome and phosphoproteome alterations during cyprid development/aging of the barnacle upon treatment with butenolide **373** was performed [101]. This analysis revealed that the expression of two groups of proteins, stress-associated and energy metabolism-related proteins, which are differentially expressed during cyprid development, was sustained by butenolide **373** [101]. Following this, the effect of butenolide on behavioral and morphological changes in the barnacle *A. amphitrite* and the bryozoan *B. neritina* was studied and butanolide was showed to decrease their phototactic behavior and attachment and inhibited secretory granules in the cement gland of *A. amphitrite* cyprids [102]. Investigations concerning the molecular mechanism of action of butenolide **373** in three fouling species, *A. amphitrite*, *B. neritina*, and *Vibrio* sp. UST020129-010 were conducted [103]. Butenolide **373**, in a pull-down assay with lysates, was found to bound acetyl-CoA acetyltransferase 1 in the barnacle *A. amphitrite*, while in the bryozoan *B. neritina*, it binds to acyl-CoA dehydrogenase, actin, and glutathione S-transferases. Finally, in the marine bacterium *Vibrio* sp. UST020129-010, butenolide **373** was found to bind succinyl-CoA synthetase β subunit and to inhibit bacterial growth [103]. Further, the authors predicted by molecular docking with models of acetyl-CoA acetyltransferase 1 of *A. amphitrite* and acyl-CoA dehydrogenase of *B. neritina*, that butanolide **373** binds to the acyl-CoA pocket or to the flavin adenine dinucleotide coenzyme pocket, respectively, influencing the binding of the substrate or the coenzyme [103]. Overall, these results suggest that butenolide **373** inhibits the fouling of these organisms by influencing their primary metabolism. A previous work also reported the differential expression of proteome and phosphoproteome in *B. neritina* larvae exposed to butanolide **373** [104]. The acute toxicity of butenolide **373** was also assessed in several non-target organisms, including microalgae (*S. costatum*), crustaceans (*Melita longidactyla*, *Tigriopus japonicus*, and *Daphnia magna*), and fish (*Lutjanus erythropterus* and *D. rerio*) [105]. Results suggest that butenolide **373** induced cell apoptosis and pericardial edema in zebrafish *D. rerio* embryos [105]. It was possible to calculate the predicted no-effect concentration (PNEC), which was among one of the highest in representative new biocides (PNEC = 0.168 µg·mL^−1^), although this value should be lower than the predicted environmental concentration (PEC), which was not possible to calculate in this study [105]. Later, chronic effects of compound **373** on oxidative stress, neurotransmission, endocrine homeostasis, and reproductive success in adult *O. melastigma* (marine medaka) were evaluated and compared to the effects of sea nine 211^®^ (Figure 1) [106]. Butenolide **373** induced at a lower extent the oxidative stress in the liver of both male and female medaka, compared to sea nine 211^®^. Moderate effects were observed on sex hormone levels in males exposed to butenolide **373**, which were gradually recovered during depuration, in contrast to sea nine 211^®^. These results show that butenolide **373** exerted transient, reversible biological effects on marine medaka [106]. The degradation kinetics of butenolide **373** under various environmental conditions were studied, and it was found that the half-life of the compound was 0.5 days in natural seawater [107]. It was concluded that the main contributor to degradation in natural seawater was caused by marine bacteria [107].

SAR studies revealed that both furan and furanone were important pharmacophores for anti-settlement activity against barnacle larvae (**364**–**366**, Figure 22) [77]. Following this, modification in the structure of AF compounds based on SAR information was performed to tune the physicochemical properties by modifying the side chain of alkyl butenolide **373** [108]. A series of butenolides (**374**–**401**, Figure 22) was obtained and the anti-settlement activity was assessed against the larvae of the barnacle *A. amphitrite* [108]. First, the impact of the nature of the side chain on anti-settlement activity was studied and 2-furanone derivatives with a five or six-carbon alkyl amine substituent at the 5-position were obtained. The results indicate that the Boc group was the most favorable terminal group as a substituent at the *N*-terminal since Boc carbamate **374** (EC_50_ = 5.43 µM) and **375** (EC_50_ = 4.00 µM) exhibited the best anti-settlement efficacy. Variation in the length of the alkyl side chain at the 5-position improved lipophilicity, which appeared to increase the AF activity and led to the development of more potent analogs (**376** and **377**; EC_50_ = 2.13 and 2.22 µM, respectively). The fact that analogs **380** and **379** (EC_50_ = 444 and 255 µM, respectively), with a high degree of hydrophilic substitution in the side chain, exhibited poor anti-settlement activity further corroborates the concept that lipophilicity of the side chain might be a key aspect for AF activity. An increase in the number of carbons in the side chain of an amine analog from five to ten carbon atoms (**380**–**385**) significantly increased the anti-settlement activity (EC_50_ = 16.7–444 µM). For amide analogs, **386**–**391** (EC_50_ = 5.48–40.3 µM), an increase in the number of carbons in the alkyl side chain also increases the anti-settlement activity. However, no enhancement in anti-settlement activity was observed when the number of carbons in the alkyl chain side was increased beyond seven carbons [108]. Using a Pearson correlation analysis, a positive association between lipophilicity and bioactivity was also demonstrated. Butenolides **376** and **377** were found to be the most potent antifoulants with desirable physicochemical properties [108].

**Scheme 10:**
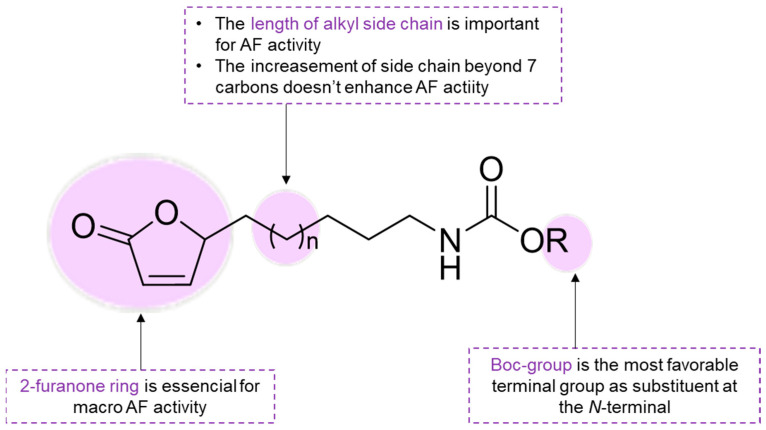
SAR for butenolide derivatives.

Twenty-one *γ*-hydroxybutenolides (**404**–**421**, Figure 22) inspired by two natural AF sesterterpene *γ*-hydroxybutenolides, previously identified from a New Zealand marine sponge, cavernosolide (**402**) and lintenolide A (**403**) (Figure 22), were synthesized and tested for AF activity against the marine bryozoan larvae of *B. neritina* and the marine algae *Isochrysis galbana* [109]. *γ*-Hydroxybutenolides **404**, **405**, **414**, **416**, **419**, **420**, and **423** were inactive against both marine organisms, while *γ*-hydroxybutenolides **406**, **407**, **408**, **413**, and **422** were only inactive against *I. galbana*. Concerning the AF activity against the marine bryozoan, efficacy decreased (higher EC_50_) with lower lipophilicity and the attachment of an aromatic group appeared to increase activity [109]. Scheme 10 summarizes SAR for butenolide derivatives.

Compounds **407**, **411**, **417**, and **418** were further selected for in situ field trials after incorporation into a rosin-modified acrylic base at a loading of ~10% (*w*/*w*). After four months, coatings containing **407** and **410** did not present any substantial fouling, as did the commercial AF reference containing Cu_2_O [109]. This work showed, once again, that natural AF compounds can be used as inspiration for the synthesis of structurally simpler analogs.

Synthetic butenolide (**373**) (Figure 22) was incorporated into a marine paint and 5% (*w*/*w*) of the compound was required to see differences between the treated surface and the control after three months submerged in seawater [77]. Following this, an optimization of the formulation containing butenolide **373** was performed [25] regarding concentrations (5, 10, and 15% (*w*/*w*)), different pigment choices, and binder compositions. After six months of submersion in seawater, coatings with 1:2.5 (acrylic copolymer and rosin) paints containing 10% butenolide had the best AF performance [25]. In the same year, a novel AF coating incorporating butanolide **373** into biodegradable poly(*ε*-caprolactone)-based polyurethane was reported [112]. Butenolide **373** can be released from the biodegradable polymer during at least three months and the rate of release depends on the concentration of the compound and the temperature. The addition of rosin into the formulation improved the late release of butenolide **373** [112]. Moreover, in another study, the modified Boc-butenolide **374** was formulated into an antifouling paint using polymer matrix poly(*ε*-caprolactone)-based polyurethane (PCL-PU), which is environmentally friendly and biodegradable [113]. Interestingly, PCL-PU/Boc-butenolide revealed a large decrease in release rate related to PCL-PU/butenolide, which can be attributed relatively to the compatibility of Boc-butenolide in PCL-PU. In the marine field test, these Boc-butenolide coatings showed good performance with low coverage of biofouler after two months [113]. Furthermore, anti-settlement bioassays against *A. amphitrite* and *H. elagans* larval suggested that Boc-butenolide has lower toxicity at high concentrations and similar AF activity than butanolide against macrofoulers, which indicates that Boc-butenolide could be a substitute in AF paints considering both antifouling effect and environmental impact [113].

Combining different bioactive ligands/pharmacophores into a single molecule is a strategy currently employed in medical research where such multi-target-directed ligands are investigated as improved drug leads. The structural motifs of butenolide **371** were fused with geraniol **425** (Figure 22) (two NPs with AF activity against the settlement of *A. amphitrite* [77]) to generate a library of AF hybrid molecules with potentially higher potency (**426**–**433**, Figure 22) [110]. This work represented an attempt to extrapolate the multi-target-directed ligands strategy into a marine setting. The major structural differences in these compounds are at the *A. amphitrite* moiety and the oxidation degrees C-5 and C-12. All hybrid molecules **427**–**433** showed AF activity and most of them with no toxicity, which suggested that the hybridization of the geraniol (**425**) and butanolide (**426**) structures led to the enhancement of the AF activity [110].

**Scheme 11:**
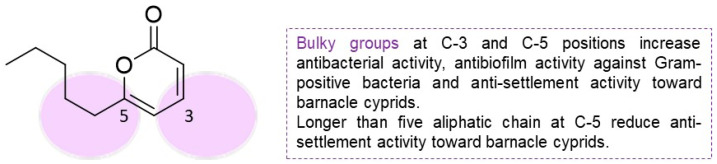
SAR for natural poliketide **434** and derivatives.

A natural polyketide, 6-pentyl-2*H*-pyrone-2-one (**434**, Figure 22), isolated from a marine-derived fungus, *Trichoderma atroviride*, and its synthetic analogs (**435**–**440**, Figure 22) were tested for potential antimicrobial activity, antibiofilm formation, and anti-settlement activity against barnacles [111]. Compounds **434**, **436**, **439**, and **440** were active against the settlement of *A. amphitrite* (EC_50_ = 8.82, 3.83, 4.32, and 4.48 µg·mL^−1^, respectively) [111]. Regarding antibacterial activity, all the tested compounds, except compound **438**, were active against Gram-positive *Loktanella hongkongensis*, while all except compound **440** were active against Gram-negative *Photobacterium angustum*. Compound **439** was the most potent compound against *Staphylococcus cohnii* and *L. hongkongensis* (MIC_50_ = 12.5 and 25.6 µg·mL^−1^, respectively) [111]. These results reveal that bulky groups, especially plane aromatic benzyl groups on both sides of the α-pyrone moiety, appeared to increase the antibacterial nature of these derivatives. The antibiofilm effects of compounds **434** and **435**–**440** (25.6 and 6.4 µg·mL^−1^) were studied using the same bacterial species. Compounds **435**, **437**, and **438** did not show any antibiofilm effects at both concentrations tested either against *L. hongkongensis* and *S. cohnii*. At 25.6 µg·mL^−1^, natural compound **434** and compound **437** exhibited only weak antibiofilm activity, while compounds **439** and **440** showed a significant antibiofilm activity towards Gram-positive *L. hongkongensis* [111]. The benzyl group at the C-3-position of the α-pyrone moiety seemed to increase the antibiofilm activity of compounds **439** and **440** compared to **437** and **435**, respectively, against *L. hongkongensis*. Moreover, the presence of a methyl group at the C-3-position of the pyrone ring was detrimental to the antibiofilm activity against the same bacteria [111]. Compound **439** was the only compound to show antibiofilm effects against *S. cohnii* at both concentrations. The presence of a benzyl group at the C-3 and C-5-positions of the α-pyrone moiety appeared to improve the antibiofilm activity (compounds **434** and **439**) against Gram-positive bacteria. Compound **439** had both macro and micro-AF-superior effects when compared with natural compound **434**, and it is a good candidate for further field testing and marine industrial application [111]. More recently, the same group confirmed the importance of a bulky or a suitable aliphatic chain (approximately 5 carbons) in C-5 and a bulky group in C-3, for a strong anti-settlement activity against *A. amphitrite* barnacles [114]. Scheme 11 evidences SAR for natural poliketide **434** and derivatives.

### 2.15. Maleimide and Succinamide Derivatives

**Figure 23:**
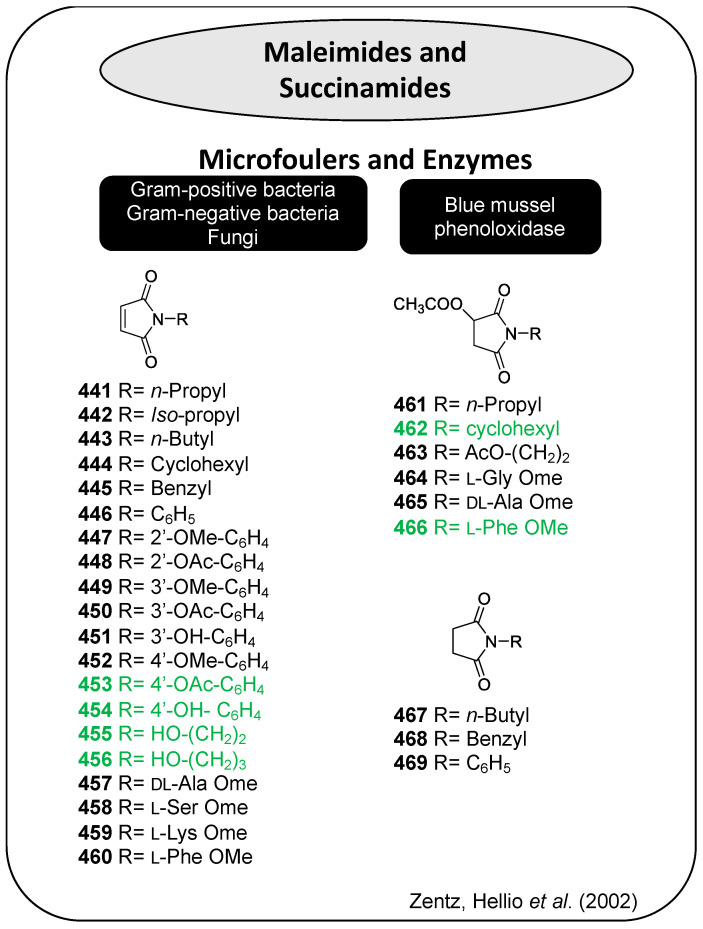
*N*-substituted maleimides (**441**–**460**) and succinimides (**461**–**469**) [115]. Green numbers: most active compounds.

Twenty-nine *N*-substituted maleimides and succinimides (**441**–**469**, Figure 23) were synthesized and their antibacterial activity against marine bacteria and fungi was tested, as well as the inhibitory activity against phenoloxidase of the byssus gland from the blue mussel *M. edulis* [115].

Compounds **442**, **444**, **445**, **447**–**451**, **457**–**459**, **461**, **463**, and **467** showed no activity against any of the marine bacterial and fungal strains tested. Compounds **453** and **462** showed specific activity against Gram-positive bacteria and marine fungi, compounds **454** and **466** showed specific activity against Gram-negative bacteria, and compounds **455** and **456** were able to inhibit both Gram-positive and Gram-negative bacteria and fungi [115]. Phenoloxidase is involved in the generation of the plaques to which the mussel threads are attached; therefore, it is a target enzyme to inhibit the settlement of this macrofouler. Compound **448**, which was not active against marine bacteria and fungi, was found to be a good inhibitor of the phenoloxidase activity (%I = 76.7; 50 µg·mL^−1^), using catechol L-DOPA as the substrate [115]. Derivative **452**, selective to Gram-negative bacteria, and derivative **462**, selective against Gram-positive bacteria and fungi, were also potent inhibitors of phenoloxidase (%I = 77.3 and 81.4, respectively; 50 µg·mL^−1^). Compound **454**, a substituted phenol, was found to be a substrate of phenoloxidase, while derivatives **441**, **445**, **462**, and **468**, were found to be noncompetitive inhibitors of phenoloxidase [115].

### 2.16. Omaezallene and Derivatives

**Figure 24:**
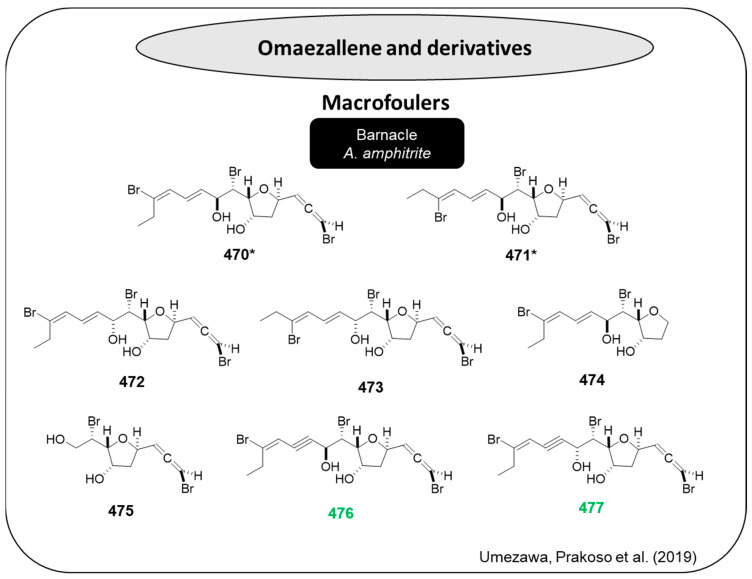
Omaezallene (**470**) and derivatives **472**–**477** [117]. * Natural products; green numbers: most active compounds.

The synthesis of natural compounds **470** and **471**, from red alga Laurencia sp., along with two synthetic derivatives (**472** and **473**), was previously reported, as well as their AF activity and toxicity [116]. The synthesis of four new derivatives of omaezallene (**470**) (**474**–**477**, Figure 24), their AF activity, and toxicity to the cypris larvae of the barnacle *A. amphitrite*, and ecotoxicity to the marine crustacean *T. japonicus* was reported [117]. Compounds **470** and **471** exhibited similar AF activity (EC_50_ = 0.46 and 0.30 µg·mL^−1^, respectively) while compounds **472** and **473** were less active (EC_50_ = 1.1 and 1.2 µg·mL^−1^, respectively) than the NP **470** [116]. The importance of the side chains (bromoallene and bromodiene functionalities) in the AF was proven by compounds **474** and **475**, which exhibited less activity (EC_50_ = 6.8 and 3.1 µg·mL^−1^, respectively) than the NP **470** [117]. Bromoenynes **476** and **477** exhibited almost the same activity (EC_50_ = 0.13 and 0.26 µg·mL^−1^, respectively) as bromodienes **470** and **471**. Additionally, derivatives **476** and **477** exhibited low toxicity towards the cypris larvae (LC_50_ = 5.5 µg·mL^−1^ for both compounds) [117].

### 2.17. Peptides

**Figure 25:**
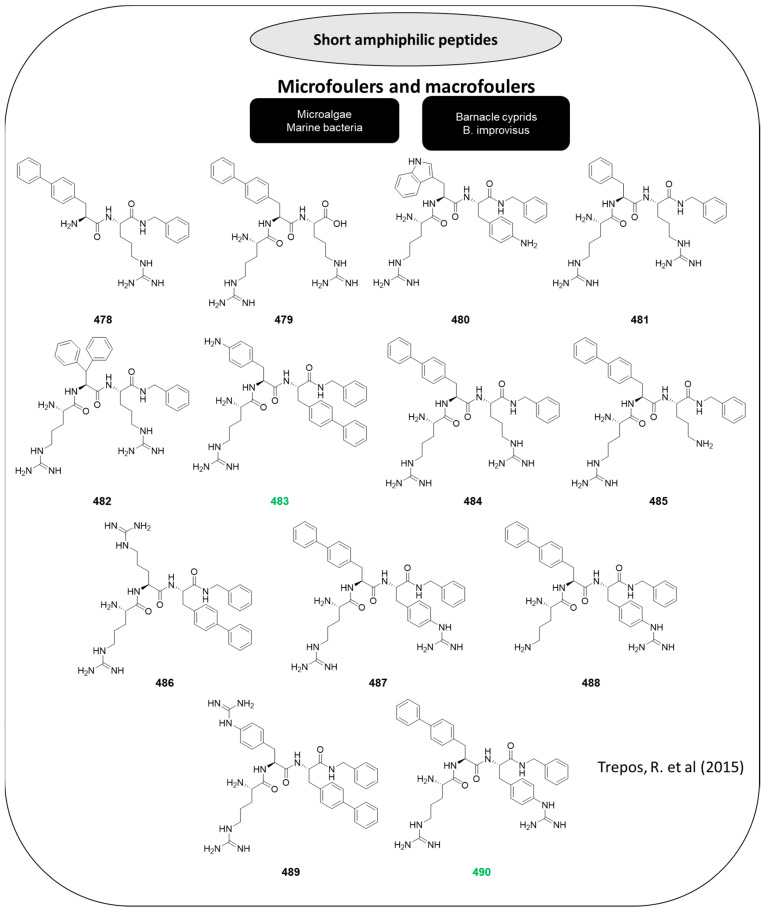
Peptides **478**–**490** [118]. Green numbers: most active compounds.

Until 2015, innate immune peptides represented a class of bioactive compounds that was under evaluated in the marine environment. A strong effect on biofilm-forming bacteria such as *Staphylococcus epidermidis* and *S. aureus* was the inspiration for evaluating the AF effect of a series of short amphiphilic micropeptides (**478**–**490**, Figure 25) [118].

Most of the investigated peptides showed a high anti-settlement effect on *A. improvisus* cyprids, with peptides **483** and **490** being the most potent compounds (IC_50_ = 1.0 and 0.5 µg·mL^−1^, respectively) [118]. A clear link between the antibacterial activity, the settlement inhibition, and the hydrophobicity of the most active peptides was observed [118]. Peptides **481**, **483**, and **487**–**490** containing one or two artificial phenylalanine derivatives, mimicking arginine and lysine, were also the most active ones, suggesting that these two structurally similar synthetic amino acids have a significant role in the AF activity. A balance between charge and hydrophobicity is needed for AF activity and a high hydrophobicity is not enough, as depicted by dipeptide **478** (IC_50_ > 5 µg·mL^−1^), lacking a cationic residue [118]. The AF activity observed for peptides **480**, **487**–**490** was reversible and cyprids displayed normal behavior after being placed in fresh seawater [118]. Additionally, none of the peptides appeared to display any toxic effects on the cyprids within the tested concentration range, suggesting a reversible nontoxic AF mechanism. Peptides **480**, **483**, and **490** were the most active compounds towards the growth and adhesion of both marine bacteria (*H. aquamarine*, *Polaribacter irgensii*, *P. elyakovii*, *R. litoralis*, *S. putrefaciens*, *Vibrio aestuarianus*, *V. carchariae*, *V. harveyi*, *V. natriegens* and *Vibrio proteolyticus*) and microalgae (*Cylindrotheca closterium*, *Exanthemachrysis gayraliae*, *Halamphora coffeaeformis*, *Pleurochrysis roscoffensis*, *Porphyridium purpureum*, *Hymenomonas coronate*, *Rhodosorus marinus*, and *Pleurochrysis carterae*) [118]. Overall, peptides **483** and **490** were the most promising displaying AF against both micro and macrofouling species.

**Figure 26:**
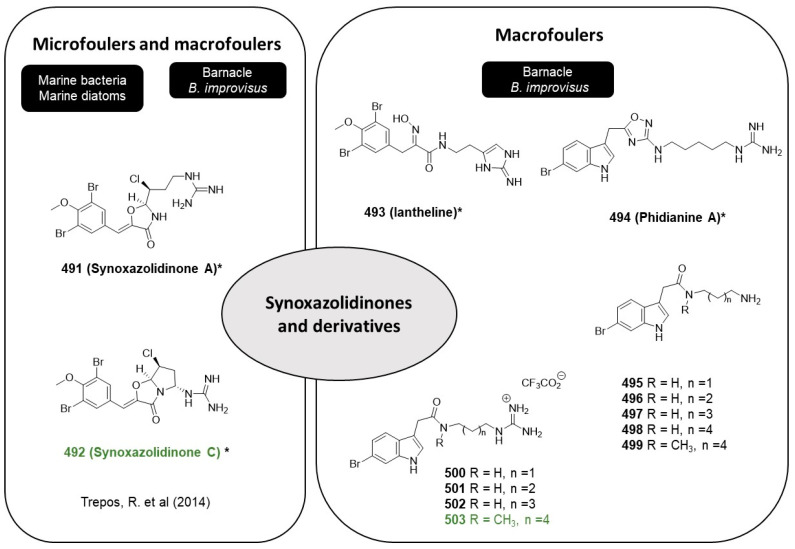
Synoxazolidinones **491**–**492**, iantheline (**493**), phidianine A (**494**), and derivatives **495**–**503** [119]. * Natural products; green numbers: most active compounds.

**Scheme 12:**
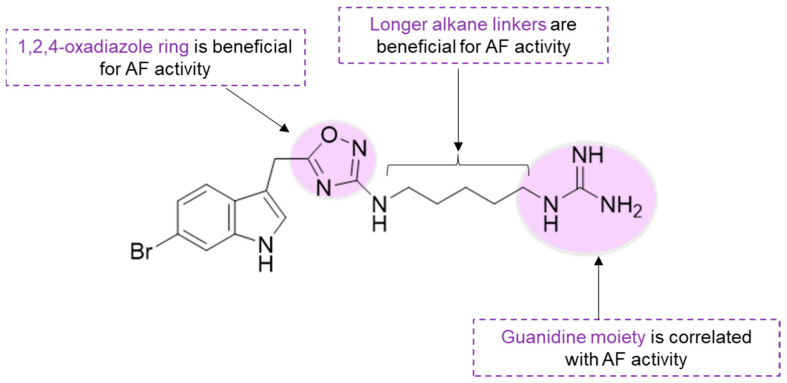
SAR for synoxazolidinone peptides.

Synoxazolidinones are a type of heterocyclic organic compound that contain an oxazolidinone ring, which is a five-membered ring with a nitrogen atom in the 3-position and a carbonyl group in the 4-position. Synoxazolidinones A (**491**, Figure 26) and C (**492**, Figure 26), isolated from the sub-artic ascidian *Synoicum pulmonaria*, are novel bromotyrosine scaffolds with powerful compounds described in the literature as potent anti-microfoulers and anti-macrofoulers [119]. These natural compounds were able to inhibit the settlement of the barnacle *A. improvisus* (IC_50_ = 15 and 2 µM, respectively) and the adhesion and the growth of marine bacteria and diatoms (MIC = 0.02–20 µM) [119]. Remarkably, while antibarnacle activity of synoxazolidinones is similar to other promising marine antifouling agents, such as barettin (**534**) and iantheline (**493**, Figure 26), the antibacterial and antialgal effects are superior [120]. Based on these data, analogues of synoxazolidinone A (**491**) were synthesized (**216**–**219**), employing the 2,5-diketopiperazine central core (Section 2.19). Dipeptic derivatives of brominated synoxazolidinones represent a particular group of MNPs with broad AF activities [120]. Ianthelline (**493**) is a potent natural antifouling compound, which inhibits the settlement and metamorphosis of barnacle crypids (IC_50_ = 6 μM) and shows antifouling effects in marine bacteria (MIC values of 0.1–10 μg·mL^−1^) [119]. Phidianidine A (**494**) was isolated from the aeolid opisthobranch mollusk *Phidiana militaris* and it is a structural analogue of ianthelline (**493**), barettin (**534**, Figure 26), and the synoxazolidinones **491** and **492**. The phidianidine A (**494**) structure comprises an 1,2,4-oxodiazole ring linked to the brominated indole system and a guanidine moiety. Remarkably, many AF bromotyrosine derivatives have a cationic guanidine or guanidine-like group [120]. A library of 10 synthetic analogs of phidianidine A (**494**) was studied for the antifouling activity against *A. improvises* cyprid and the inhibition results were compared with the positive control sea nine 211^®^ and potent MNPs (ianthelline, barettin, and synoxazolidinone A) [119]. Compound **494** presented an IC_50_ value of 4.0 μg·mL^−1^, which is comparable to the reference molecules and it caused a 3% mortality of cyprids at a concentration of 5 μg·mL^−1^, so this compound exerts its antifouling effect through a non-toxic mechanism at the employed concentration. Interestingly, synthetic analogs **502** and **503** demonstrated promising antifouling activity, with lower IC_50_ values related to compound **494** at 2.2 and 0.7 μg·mL^−1^, respectively [119]. In this way, compound **503** was revealed to be the most effective inhibitor, with better antifouling performance than several robust antifouling MNPs. Additionally, compound **503** displayed low toxicity against the target organism [119]. Compounds **494**, **502**, and **503**, with longer alkane linkers, were more potent, the presence of a 1,2,4-oxadiazole ring was not essential (as compounds **502** and **503**, without 1,2,4-oxadiazole ring, were more potent than compound **494**), and the basicity of the cationic group seems to increase the AF activity. Important molecular features of synoxazolidinones and derivatives are highlighted in Scheme 12. Compound **503** (Figure 26) was selected to be incorporated in a biodegradable poly(*ε*-caprolactone-co-*δ*-valerolactone) polymer for AF applications [119]. The anti-macrofouling activity of compound **503** was not possible to observe since none of the panels, including the control, had macroorganisms. Nonetheless, the biofilm formation was analyzed by confocal laser scanning microscopy and it was possible to observe a reduction in the biofilm mass in the panel containing compound **503** [119]. Therefore, compound **503** was found to be the most potent anti-macrofouling compound of this series of synthetic derivatives of compound **494** and also showed anti-microfouling activity in the field tests [119].

**Figure 27:**
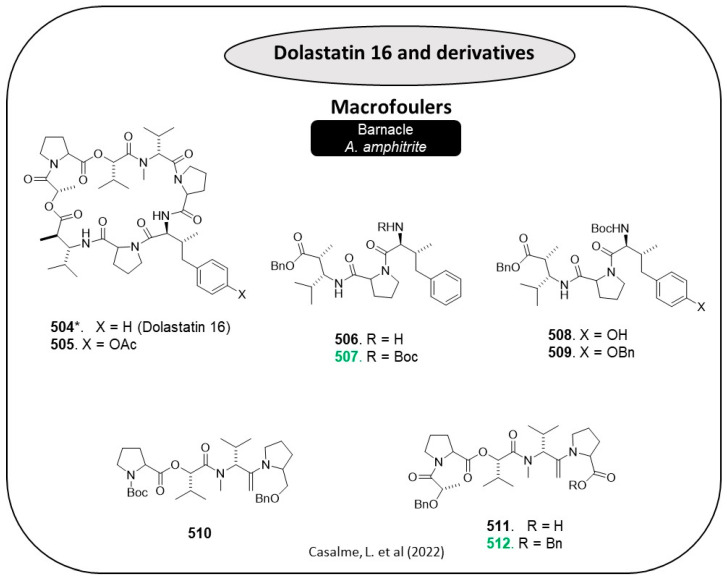
Dolastatin 16 (**504**) and its derivatives **505**–**512** [121]. * Natural products; green numbers: most active compounds.

Dolastatin 16 (**504**, Figure 27) is a natural depsipeptide product obtained from the sea hare *Dolabella auricularia* and contains two rare amino acids, dolafenvaline and dolamethylleuin, and was first reported by Pettit and his colleagues in 1997 [121]. In 2010, Tan and co-workers demonstrated robust activity of dolastatin 16 (**504**) toward the larval settlement and metamorphosis of barnacle *A. amphitrite* with EC_50_ and LC_50_ values of 0.003 and 20 µg·mL^−1^, respectively, being a promising lead compound for the development of novel AF materials [122]. In 2017, it was reported that the AF activity of dolastatin 16 (**504**) and two intermediates, northern carboxylic acid fragment **511** and southern amine fragment **506**, showed the highly potent activity of **504** (EC_50_ < 0.03 µg·mL^−1^) and moderate to low activities of **511** and **506** (EC_50_ > 10 and 1.17 µg·mL^−1^, respectively) against barnacle *A. amphitrite* [121]. In 2022, derivatives of dolaphenvaline and dolamethylleuine, as well as some derivatives of compound **504** were synthesized and investigated to analyze their potential activity toward the development of a green AF material [121]. The compounds evaluated (**504**–**512**) are shown in Figure 27 [121].

All samples showed AF profiles with low toxicity against cypris larvae of the barnacle *A. amphitrite* [121]. At the level of SAR, the introduction of a functional group on the aromatic ring of compound **504** reduced the AF activity to moderate (**505**, EC_50_ = 1.74 µg·mL^−1^). Compounds **507**, **508**, and **512** were revealed to be more active with EC_50_ values below 1 µg·mL^−1^. Protection of the southern fragment with a Boc group enhanced the EC_50_ value (**507**, EC_50_ = 0.79 µg·mL^−1^), which can be attributed to its higher hydrophobicity than **506** (EC_50_ = 1.17 µg·mL^−1^) by the protection of the amino group. The functional groups at the *p*-position of the aromatic ring affected the AF activity of the southern fragment due to steric bulkiness: a hydroxy group (**508**, EC_50_ = 0.60 µg·mL^−1^) had a slightly decreased EC_50_ value compared to **507**, but a benzyloxy group (**509**, EC_50_ = 4.62 µg·mL^−1^) abruptly reduced the AF activities to 4.62 µg·mL^−1^. A benzyl ester of the northern fragment (**512**, EC_50_ = 0.90 µg·mL^−1^) revealed much higher potency than **511** (EC_50_ > 10 µg·mL^−1^), suggesting the higher hydrophobic fragment was more active than the corresponding more polar one. A benzyl ether (**510**, EC_50_ = 3.27 µg·mL^−1^) decreased the AF activity, which suggests the lactate moiety or the carbonyl group for the northern fragment are crucial factors [121].

### 2.18. Phenyl Ether Derivatives

**Figure 28:**
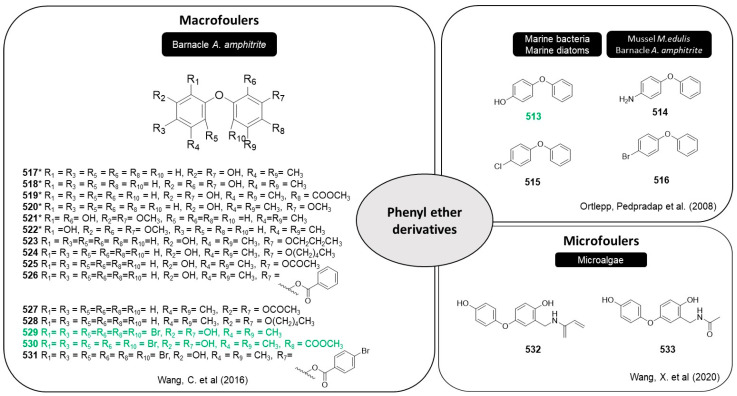
Biphenyl ether derivatives [123,124,125]. * Natural products; green numbers: most active compounds.

Natural-occurring biphenyl ethers were found to possess AF activity along with four synthetic biphenyl ethers (**513**–**516**, Figure 28) against a panel of several fouling organisms, such as mussel, barnacle, diatom, and bacteria isolated from marine biofilms [123]. All four synthetic compounds inhibited the attachment of the mussel *M. edulis*, with derivative **514** being the most potent compound (EC_50_ = 110 nM), and the growth and attachment of the benthic diatom *H. coffeaeformis*, with compound **513** being the most potent (EC_50_ = 7.74 µM). Regarding antibacterial activity, only diphenyl ether **514** did not inhibit the growth of *Bacillus* sp. or *Zooshikella* sp. Compound **513** demonstrated the strongest antibacterial activity (MIC = 0.76–1.90 µM). The presence of hydroxy in compound **513** may be influencing the marked AF activity. Nevertheless, compounds **513**–**515** were found to be toxic to cyprids of *A. amphitrite* [123].

Phenyl ether derivatives **517**–**522** (Figure 28) were isolated from the fungus *Aspergillus* sp. XS-20090066. The phenyl ether derivatives **523**–**531** (Figure 28) were synthesized by structure modification from diorcinol (**517**) and 4-methoxycarbonyl-diorcinol (**519**). All these compounds were tested for their AF activities against the cyprids of barnacle *A. amphitrite* [124]. Comparing the natural phenyl ethers **517**–**522**, 4-methoxyacyl-diorcinol (**519**) showed the most robust AF activity (EC_50_ = 7.43 μM), while others showed moderate activity (EC_50_ = 18.2–57.3 μM). It is important to mention that compound **519** exhibited four times stronger activity than diorcinol (**517**) (EC_50_ = 32.6 μM). In a structural point of view, the ester group substitution at C-4 increases the activity. A hydroxy substitution at C-2, as in compound **518** (EC_50_ = 57.3 μM), decreased the activity. Methoxy substituted at C-3 in compound **520** (EC_50_ = 31.0 μM) did not show an impact on activity [124]. The alkylated (compounds **523**, **524**, and **528**) and acylated (compounds **525**–**527**, **531**) synthetic phenyl ether derivatives revealed stronger AF activity (EC_50_ = 2.23–27.9 μM) than the original compound **517**. Concerning to the alkylated derivatives, compound **523** with a propionyloxy group substitution at C-3 had the most promising activity (EC_50_ = 9.82 μM). Relative to the acylated products, a benzoyloxy substitution at C-3 (compound **526**; EC_50_ = 12.6 μM) increased the activity, but an acetoxy substitution (compound **525**) (EC_50_ = 3.05 μM) and a *p*-bromobenzoyl substitution (compound **531**; EC_50_ = 2.42 μM) caused a significant increase in the activity. In this line, smaller acetoxy substitutions demonstrated to be better than larger benzoyloxy substitution at C-3 to improve the AF activity profile. Moreover, the introduction of a bromine atom could improve the activity (compound **531**). Corroborating that, compounds **529** and **530** (EC_50_ values of 0.71 μM and 1.17 μM, respectively) also showed robust anti-larval *A. amphitrite* settlement [124].

**Scheme 13:**
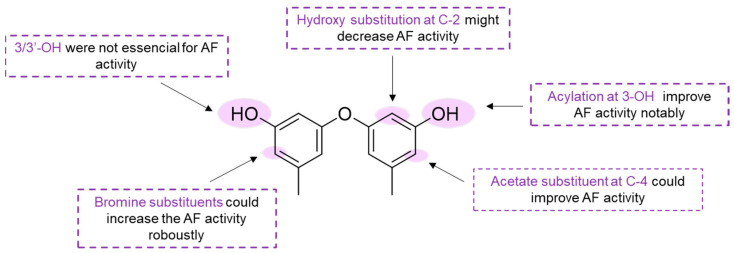
SAR for diterpene derivatives.

On one hand, the hydroxyl groups at C-3/C-3′ were not shown to be essential for the AF activity, and hydroxy substitution at C-2 might decrease AF activity. On the other hand, acylation at 3-OH and acetate substitution at C-4 could improve AF activity. Remarkably, bromine substituents could increase the AF activity [124]. Scheme 13 summarizes the SAR for biphenyl ether derivatives.

From a toxicological view, derivative **523** showed toxicity with a therapeutic ratio of 11.4. In contrast, the phenyl ether derivatives **525**, **527**, and **529**–**531**, demonstrated low toxicity with LC_50_/EC_50_ ≥ 21.1. Interestingly, the polybrominated diphenyl ether derivative **529** showed the most favorable therapeutic ratio, with a value higher than 31.0. In this way, compound **529** was considered a promising candidate for environmentally friendly AF agents [124].

In another study, phenyl ether derivatives of capsaicin were (**532** and **533**, Figure 28) shown to be able to inhibit algal growth of *P. tricotornum*, *S. costatum*, and *Chaetoceros curvisetus* [125]. It was demonstrated that the capsaicin synthetic derivatives were excellent algaecides and AF agents. Specifically, compound **533** revealed better AF performance compared to compound **532.** Regarding the mechanism of action, the derivatives interfere with the permeability and structure of the algal cell membrane. Moreover, the marine field tests showed that the addition of compounds **532** and **533** as environmentally friendly auxiliaries to coatings could be a promising strategy to achieve long-term marine fouling resistance [125].

### 2.19. Piperazines

**Figure 29:**
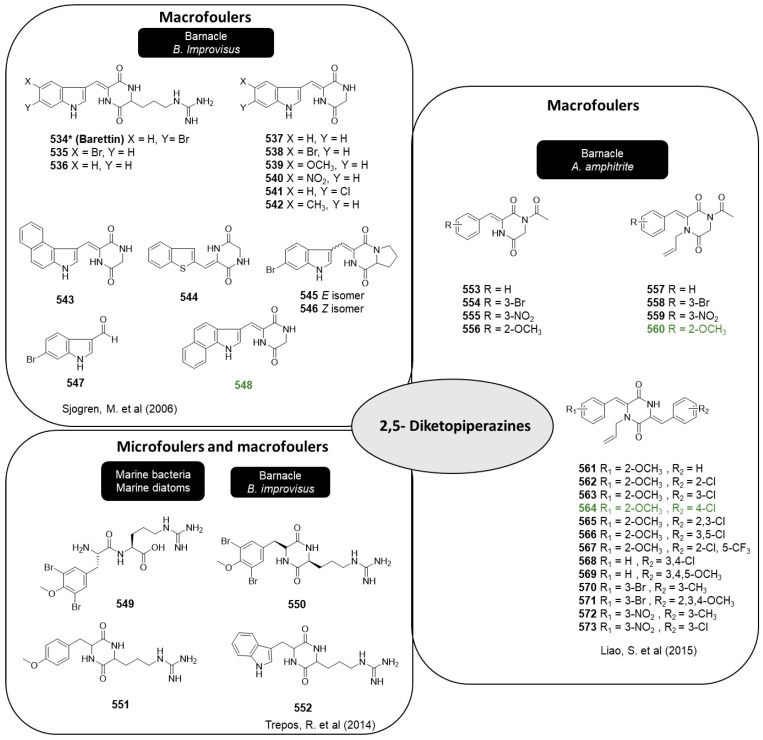
2,5-Diketopiperazines [120,127,128]. * Natural products; green numbers: most active compounds.

2,5-Diketopiperazines derivatives are a class of naturally occurring privileged structures from fungi, bacteria, the plant kingdom, and mammals [126]. Barettin (**534**, Figure 29), a diketopiperazine isolated from the marine sponge *Geodia barretti*, is a potent AF agent, and its effect on barnacles can be attributed to the 2,5-diketopiperazine core and an exocyclic double bond to the brominated indole [120]. To increase the knowledge into the SAR of barettin (**534**) a series of 14 analogs (**535**–**548**, Figure 29) was obtained, and their activity was evaluated against the larval settlement of *A. improvises* [127].

Slight modifications in the structure of these analogs had a high impact on their ability to inhibit the settlement of the larvae. The authors focused on the modifications of the position of bromine in the indole residue of tryptophan. When the bromine was changed from the 6-position to the 5-position (**535**) stimulation of the settlement of *A. improvisus* was observed (EC_50_ = 4.1 µM), while the removal of the bromine at the 6-position resulted in a complete loss of activity (**536**) [127]. Regarding dipodazine derivatives, once again, the presence of bromine in the 6-position (**545** and **547**) resulted in significant inhibition of the larval settlement of *A. improvisus* (EC_50_ = 2.4 and 6.7 µM, respectively). However, in dipodazine (**537**) the insertion of methyl in the 5-position (**542**) did not lead to the stimulatory effect of 5-bromobarettin (**535**). Compounds with methoxyl and nitro groups in the dipodazine scaffold (**539** and **540**) did not show any settlement inhibition. Substitution of the bromine for chlorine resulted in the loss of activity (**541**), suggesting that the atom size in that position is critical for the activity. Compound **548** was the most potent compound against the settlement of barnacle larvae (EC_50_ = 0.034 µM) [127]. This result could indicate that the highly non-polar phenyl in the 6,7-position of benzo[*g*]dipodazine present in compound **548** is electronically similar to the bromine in the 6-position present in barettin (**534**). The position of the phenyl ring in compounds **543** and **548** influenced the AF activity displayed by these compounds, being that compound **548** is more active than compound **543**. When the phenyl was substituted for a methyl group (**542**), no activity was observed. The stereochemistry of the double bond of compounds **545** and **546** seems to play an important role in the inhibition of the settlement of barnacle larvae since the isomer *E* (**545**, EC_50_ = 2.4 µM) was active, while the isomer *Z* (**546**) was inactive [127].

A small library of synthetic analogues of synoxazolidinone A (compound **491**, Section 2.17) employing the 2,5-diketopiperazine central core found in barettin (**534**), as a replacement for the synthetically more challenging 4-oxazolidione core, was prepared based on the high activities of synoxazolidinone A (compound **491**) and synoxazolidinone C (compound **492**) [120]. Comparing the natural compounds (**491** and **492**) with the synthetic analogues (**549**–**552**), it is possible to infer that the effect on microfouling can be maintained using a 2,5-diketopiperazine scaffold [120]. However, the effects on microalgae are lower for the derivatives and the elimination of bromine led to the almost inactive compounds (**551** and **552)** against microalgal growth and adhesion [120]. Interestingly, none of the synthetic compounds were able to interfere with *A. improvisus* larvae, suggesting that a certain degree of structural integrity is required for macro AF activity. In this way, the use of a saturated 2,5-diketopiperazine scaffold is not a crucial requirement for active macro AF activity, and the presence of a double bond is essential for high inhibitory activity toward barnacles and microfouling species [120].

A small library of soluble 2,5-diketopiperazine derivatives was synthesized and evaluated against *A. amphitrite* larval attachment [128]. From the first series of compounds (**553**–**560**, Figure 29), only compound **560** (EC_50_ = 20.5 µg·mL^−1^) showed AF effects, which indicate that 2-OCH_3_ might be contributing to the AF effects. For this reason, compound **560** was selected as a template molecule for the synthesis of other 2,5-diketopiperazine derivatives (**561**–**573**). The new series showed better AF activity, with compound **564** exhibiting the most potent activity against *A. amphitrite* (IC_50_ = 1.6 µg·mL^−1^) with low or no toxicity (LC_50_ = 25 µg·mL^−1^) and could be a new template molecule for further development as environmentally friendly AF agent [128].

**Figure 30:**
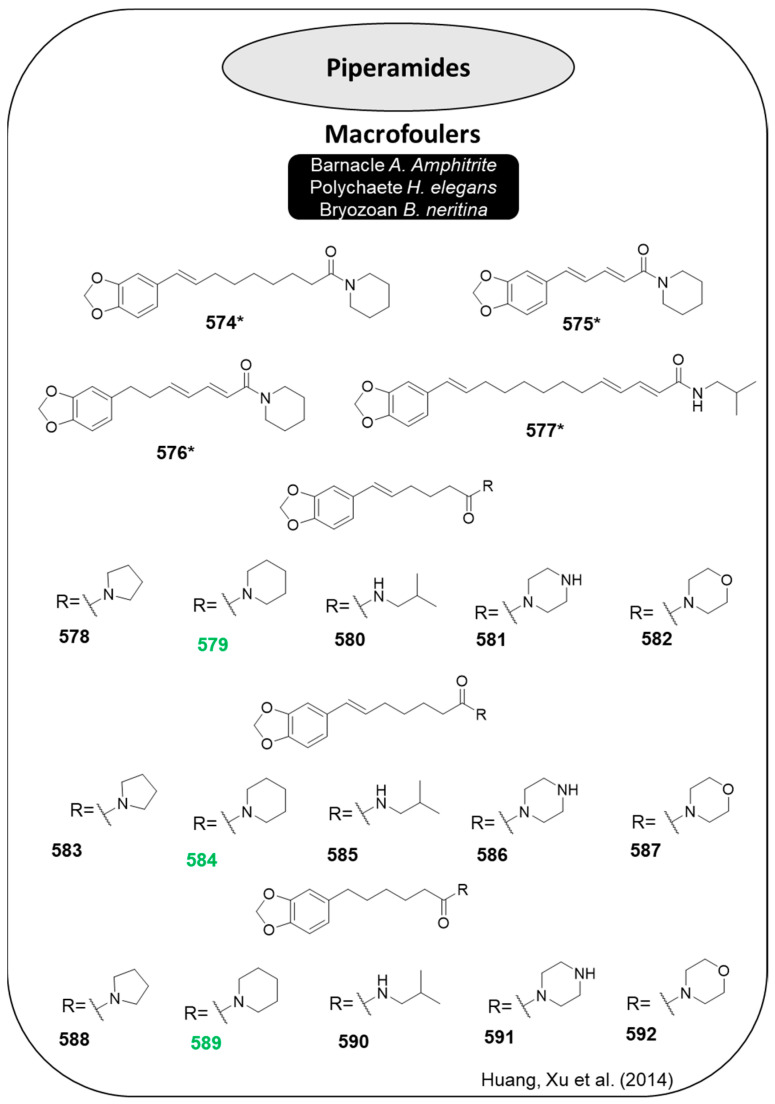
Piperamides **571**–**589** [130]. * Natural products; green numbers: most active compounds.

**Scheme 14:**
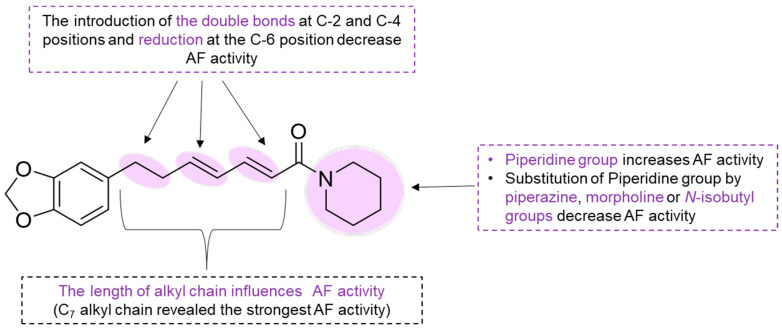
SAR for piperamides.

Piperamide is a substituted piperazine with a tertiary amine group and was used as an anthelmintic agent due to its effectiveness against *Trypanossoma* [129]. The AF activity of an acetone extract of the terrestrial plant *Piper betle* was evaluated and four structurally similar AF piperamides were isolated (**574**–**577**, Figure 30) [130]. Following this, 15 piperamide analogs (**578**–**592**, Figure 30) were synthesized and their AF activity was compared to determine SAR among this class of compounds [130]. The effects of natural piperamides **574**–**577** in the larval settlement and toxicity were assessed using cyprids of the barnacle *A. amphitrite*, larvae of the polychaete *H. elegans*, and larvae of the bryozoan *B. neritina*. The AF activity of piperamide analogs containing modified side chains was evaluated against the settlement of the barnacle *A. amphitrite*. Piperidine amides **574**, **579**, **584**, and **589** exhibited the highest anti-settlement activity (EC_50_ = 2.3, 1.3, 0.5, and 1.5 µg·mL^−1^, respectively), indicating that the piperidine group was the optimal group at the *N*-terminal [130]. Increased AF activity might be related to the lipophilicity of the amine moieties, as compounds with the piperazine, morpholine, or the *N*-isobutyl groups had significantly weaker AF activity compared to compounds containing the piperidine or the pyrrolidine groups. Among piperamide analogs with the piperidine group, compound **584**, containing a C_7_ alkyl chain, revealed the strongest AF activity, showing that the length of the alkyl chain influences AF activity. The introduction of the double bonds at the C-2 and C-4-positions and reduction in the double bonds at the C-6-positions decreased AF activity, as seen in compound **576** (EC_50_ = 4.2 µg·mL^−1^) [130]. Scheme 14 summarizes the SAR for piperamides. Toxicity against zebrafish embryos was investigated for the two most active compounds, piperamides **574** and **584**, and no effect was observed [130].

### 2.20. Polyphenol Derivatives

**Figure 31:**
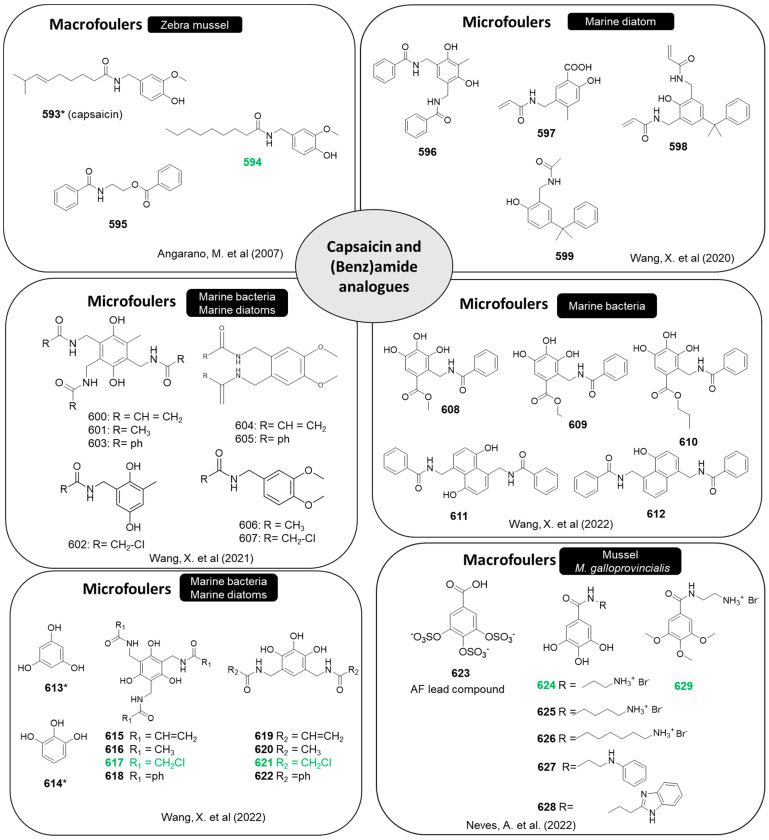
Capsaicin (**590**) and amide derivatives [36,125,131,132,133,134]. * Natural products; green numbers: most active compounds.

Capsaicin (**593**, Figure 31), 8-methyl-*N*-vanillyl-6-nonenamide, is isolated from *Capsicum* sp., and it exhibits promising AF activity [131]. Capsaicin became an important lead compound in the research of novel AF compounds. Capsaicin (**593**) and two other commercially available compounds with one or more capsaicin-like structural features (**593**–**595**, Figure 31) were tested against the settlement of zebra mussel (*D. polymorpha*). Lethal effects were investigated for the most promising compounds against the non-target organism *D. magna* [131]. Significant AF activity was observed for compounds **593**, **594**, and **595** (EC_50_ = 13.7, 17.7, and 25.7 µM, respectively). From the three active compounds, compound **594** was the most promising, as it was able to inhibit zebra mussel byssal attachment at concentrations that had no significant lethal effects on this species or the non-target species *D. magna* [131].

In another study, six capsaicin derivatives were synthesized to obtain environmentally friendly marine AF agents, two of which are diphenyl ethers (**532** and **533**—Figure 28, Section 2.18 and **596**–**599**—Figure 31). Structural analogs of capsaicin were decorated with distinct functional groups, particularly, phenyl, phenolic hydroxyl, amide, and alkeny groups [125]. Capsaicin derivatives were shown to be able to inhibit algal growth of *P. tricotornum*, *S.costatum* and *C. curvisetus* [125]. The inhibition order of the six compounds on algae was compounds **599** > **598** > **596** > **597**. Compound **599** revealed the best inhibition effect, with an inhibition rate of approximately 95% at 3 mg·L^−1^ [125].

Regarding toxicity evaluation, most compounds showed low toxicity to algae and EC_50_ values (72 h) were lower than 250 mg·L^−1^. The toxicity of compound **599** to *S. costatum* was intermediate (0.3–3 mg·L^−1^, and all other compounds demonstrated low toxicities (more than 3 mg·L^−1^). Remarkably, compounds **596** and **597** have no significant toxicity to *P. triconurtum* and *S. costatum* (150 mg·L^−1^). In this way, the toxicity of compounds **598** and **599** was greater than compounds **596** and **597** [125].

In another study eight capsaicin derivatives (**600**–**607**, Figure 31), decorated with distinct amide groups on the benzene ring, were evaluated for the antibacterial, anti-algal, and AF activities [132]. To evaluate the AF performance of capsaicin derivatives, its antibacterial activity against *E.coli* and *S. aureus*, as well as the anti-algal effect and toxicity against *N. closterium* and *Chlorella vulgaris*, were determined [132]. The capsaicin derivatives showed robust antibacterial effects against *E.coli* and *S. aureus*, with a small number of colonies on the test board compared to the control board. From a structural point of view, amide groups can impact the electron transport system of the bacterial respiratory chain, leading to their death. Moreover, compounds with phenol groups, **600**, **601**, **602**, and **603** showed better antibacterial activity than compounds **604**, **605**, **606**, and **607**, which revealed that phenol groups are a key to antibacterial activity. Compounds **604**, **606**, and **607** showed better antibacterial activity compared to compound **605**, which suggests that antibacterial activity can be directly related with the length of the side chain [132]. Overall, the benzene ring and amide group are crucial for antibacterial activity and the phenolic hydroxy group is the preferred group. The similar inhibitory effects of capsaicin derivatives are probably related to damaging effects on the cell membranes of *E. coli* and *S. aureus*. The anti-algal activity of capsaicin derivatives increased with the enhancement of time and concentration. The compounds were shown to be environmentally friendly anti-algal agents, revealing much less toxicity compared with the currently used antifoulants, such as TBT and sea nine 211^®^ (Figure 1) [132]. Compounds **603** and **602** exhibited excellent anti-algal activity (I > 77.30% against *N. closterium* and I > 82.50% against *C. vulgaris*), compared with compounds **600** and **601**. Compounds **607** and **606** revealed more promising anti-algal activity compared with compounds **604** and **605**. Moreover, compounds **600**, **601**, **602**, and **603** showed higher anti-algal activity than **604**, **605**, **606**, and **607** [132]. From a structural point of view, these results allow us to conclude that the presence of benzene rings and amide groups are crucial for anti-algal activity and phenolic groups and chlorine atoms are important to improve the anti-algal activity. In addition, the increase in the number of benzene groups improves the anti-algal activity. In situ AF assay was carried out for six months to evaluate the AF effects of the eight capsaicin derivatives (**600**–**607)** as AF adjuvants by incorporating them in coatings containing zinc acrylic resin, Cu_2_O, rosin resin, and other auxiliaries, and is then applicated on PVC panels. The control plate had many microorganisms and algae attached, while the test plates only presented a small number of microorganisms adhered [132]. Remarkably, capsaicin derivatives reveal excellent AF performance in the marine environment, eco-compatibility, and slow release rates as AF agents compared with the natural molecule capsaicin, which is easily soluble in water and shows a less promising AF effect and durability in seawater [132]. In a following work, benzamide derivatives containing capsaicin (BDCC; **608**–**612**; Figure 31) were synthesized, being the aromatic moieties the methyl gallate (**608**), ethyl gallate (**609**), propyl gallate (**610**), 1,5-dihydroxynaphthalene (**611**), and α-naphthol (**612**) [133]. BDCC showed antimicrobial activity against *S. aureus* and *E. coli*. Remarkably, compounds **608**, **609**, and **610** revealed better antimicrobial effects than compounds **611** and **612**. Interestingly, all the compounds revealed better inhibitory effects on *E. coli* than those on *S. aureus*. This can be attributed to the abundance of peptidoglycan layers in cell walls, which is higher in *S. aureus*, which makes the permeation of benzamide derivatives difficult. From a structural point of view, the presence of benzene rings, phenolic hydroxyl groups, ester groups and amide groups was demonstrated to be related to good antimicrobial activity. Moreover, the substitution of benzamide groups on aromatic compounds increased the antimicrobial activities of aromatic compounds. In addition, phenolic hydroxyl groups were shown to be associated with the antimicrobial activities of benzamide derivatives. In this line of thinking, the charge differences between H and O atoms in the phenolic hydroxyl groups of compounds **609**, **610**, **608**, **611**, and **612** decreased in turn, which is closely related to antimicrobial activities of compounds, explained by the reaction with protein through hydrogen bonds and the formation of precipitates [133]. BDCC compounds (**608**–**612**) were incorporated in the same previously used coating formulation [132,133]. The AF assay was carried out for 180 days and it was verified that only a small amount of microorganisms and a small number of barnacle larvae were attached to test plates containing capsaicin derivatives, compared to control plate [133]. Thus, the results suggest that the AF coating formulation shows a good repellent effect on microorganisms and the good hardness and adhesion of AF coating [132,133]. Specifically, the more stable compounds **608**–**610** showed the most promising AF activities after 6 months [133]. The AF effect of **611** was better than **612**, which can be attributed to its greater polarity and, consequently, its higher solubility, which allowed a more uniform and effective AF coating [133]. Due to the combination of BDCC with Cu_2_O, and zinc acrylic resin, benzamide derivatives performed optimal AF properties. Moreover, these compounds presented uniform distribution, good thermal stability, and high surface tension [133].

Concerning the mechanism of action, it was shown that the antibacterial activity of capsaicin can be attributed to its ability to change the cytomembrane fluidity, which led to the disruption of cell membranes [125]. Moreover, it was also demonstrated that capsaicin derivatives inhibit algal growth by increasing the membrane permeability, without causing cell death, proving their potential as environmental friendly AF agents [125]. Remarkably, the AF mechanism of capsaicin on algal species was related to cytosolic Ca^2+^. Particularly, the increase in intracellular Ca^2+^ caused the unicellular green alga *Chlamydomonas reinhardtii* to lose flagella (deflagellation), loss of exercise ability, and other cell function disorders, and the same inhibition effect was confirmed among five species in *Chlorophyta* sp. Moreover, it is speculated that the AF mechanism of capsaicin on *Ulva* sp. may be due to the rapid increase in intracellular Ca^2+^ as the result of the activation of the transient receptor potential vanilloid 1 (TRPV1) channel by capsaicin, making the spores hard to settle, or other functional disorders [135].

To improve AF performance of phloroglucinol (**613**, Figure 31) and pyrogallol (**614**, Figure 31), eight polyphenol derivatives (**615**–**622**, Figure 31) were synthesized with the purpose to improve the AF activity, being that the antibacterial activity of the derivatives was much higher than the original polyphenols [134]. The polyphenol derivatives containing phenolic hydroxyl and amide groups demonstrated similar antibacterial activities due to their similar structures. Compounds **617** and **621**, containing chlorine atoms, showed the most effective antibacterial activity, indicating that the addition of chlorine atoms could improve the antibacterial activity [134].

The effects of polyphenol derivatives on the growth of *C. vulgaris* and *N. closterium* were evaluated by an algal assay. Compounds **617**, **619**, **620**, **621**, and **622** revealed superior inhibitory effects with inhibition rates higher than 93% for *C. vulgaris* at 10 days. For *N. closterium*, their inhibition rates were higher than 57% and the anti-algal activity followed the ascending order **622**, **619**, **620**, **617**, and** 621**. Compounds **615**, **616**, and** 618** did not show promising anti-algal activity. From a structural point of view, compounds with chloroacetamide groups, such as **617** and** 621**, revealed better anti-algal activity for both species than compounds with acrylamide, acetamide, and benzamide groups [134]. The inhibition rates of compounds **614**, **619**, **620**, **621**, and** 622** were higher than **613**, **615**, **616**, **617**, and** 618**, respectively. Suggesting the importance of chlorine atoms in algal inhibition activity, EC_50_ values of compounds **619**, **620**, and** 622** were significantly higher than **617** and** 621** [134].

**Scheme 15:**
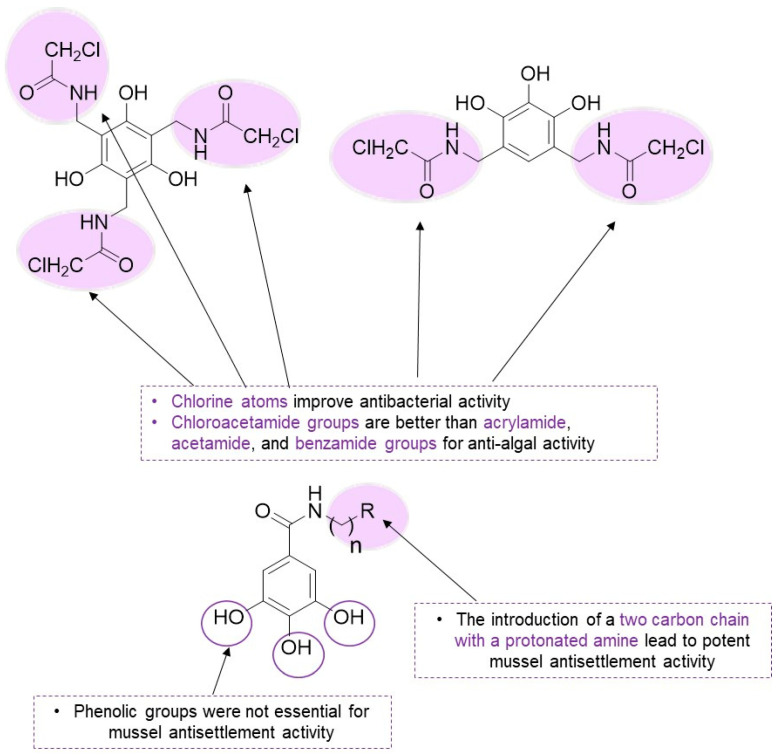
SAR for polyphenol derivatives.

These polyphenol derivatives (**615**–**622**) were added to coatings containing acrylic resin, Cu_2_O, rosin resin, and other auxiliaries and applicated in PVC panels. The test panels were submerged on seawater for six months. After three months, the control panel was covered with macroorganisms, test panels with polyphenol derivatives showed limited biofilm formation and panels with compounds **616**, **617**, and **620** showed a few settled barnacles. After six months, only biofilm formation and a few settled barnacles were observed in test plates. It is important to note that panels with compounds **618** and **621** were clear of macrofouling, instead of control panels, which were covered with macroalgae and barnacles [134]. Overall, some polyphenol derivatives revealed potential for the development of environmentally friendly AF applications and show high antibacterial, anti-algal, and AF activity. The addition of chlorine and amide groups revealed to be favorable to increase the activity of these derivatives [134]. Concerning the mechanism of action, polyphenol derivatives with more hydroxyl groups that show tannic properties can cause protein coagulation, resulting in protein denaturation, protein precipitation, and inactivation of the enzyme system [134]. A series of six gallic acid derivatives (**623**–**629**, Figure 31) containing different amine groups was also synthesized to increase potency and decrease water solubility while maintaining low toxicity of a non-toxic water soluble nature-inspired AF compound, gallic acid persulfate (compound **623**), against *M. galloprovincialis* larvae (EC_50_ = 17.65 µM; LC_50_/EC_50_ = 26.61) [36]. The lead optimization strategy comprised the introduction of amine/amide groups, triazole ring, and halogen substituents, described as molecular features correlated with AF activity. In the presence of compounds **624** and **629**, larvae settlement decreased to <35%, and these compounds revealed promising AF activity against *M. galloprovincialis* larvae related to the lead compound, with EC_50_ values of 2.74 µM and 16.28 µM, respectively [36]. Remarkably, removing the sulfate groups and maintaining the hydroxy groups, as well as the introduction of a carbon chain with a primary amine, led to the most potent derivative, compound **624**, with a seven-fold higher potency than gallic acid persulfate, and did not cause mortality to the target species even at concentration 73-fold higher than the EC_50_ [36]. Scheme 15 summarizes the SAR for polyphenol derivatives.

Regarding the mechanism of action, the activity of enzymes AChE and tyrosinase was not significantly impacted after exposure to the most promising compounds (**624**, and **629**) [36]. In the ecotoxicological evaluation, it was found that compound **624** is non-toxic to *A. salina* (<10% mortality at concentrations of 25 and 50 µM), similarly to gallic acid persulfate. However, in contrast to the lead compound **623**, compound **624** was classified as non-toxic for the marine diatom *P. tricornutum*. Moreover, amide derivative **624** reveals a reduced value of Log octanol-water partition coefficient, which suggests its low potential for bioacummulation in marine organisms [36].

Due to its AF performance, compound **624** (Figure 31) was incorporated as an additive in a PU-based marine paint composed of a base resin and a curing agent to evaluate its viability as an AF agent in commercial marine coatings. Compound **624** demonstrated good compatibility with the PU-based coating, revealing a better behavior than its precursor gallic acid persulfate (**623**, Figure 31). The results show that compound **624** was effective in decreasing the fixation of mussel larvae, presenting a significant difference compared with free PU-based coating after 40 h of exposure. Concerning the leaching of compound **624**, a value lower than 10% of this compound was detected in artificial seawater after 45 days [36].

Moreover, to increase the service life of these coatings, further coating optimization was performed through chemical immobilization using the trimethylolpropane triaziridine propionate (TZA) crosslinker (CL) [136]. In the antibiofilm assay in *P. tunicata*, the biofilm biovolume was significantly lower for the new PU-based marine coating containing 2% (*w*/*w*) **624** and the CL (**624**/PU/CL) surface when compared to coating without CL. Remarkably, **624**/PU/CL provided the best long-term performance. Moreover, in **624**/PU/CL coating, the number of cells only started to increase from day 28 and the number of biofilm cells was lower compared with **624** PU-based coatings, which suggests the compatibility of the compound **624** in this polymer matrix and the service life of the generated matrix were improved due to TZA [136].

### 2.21. Portimine and Derivatives

**Figure 32:**
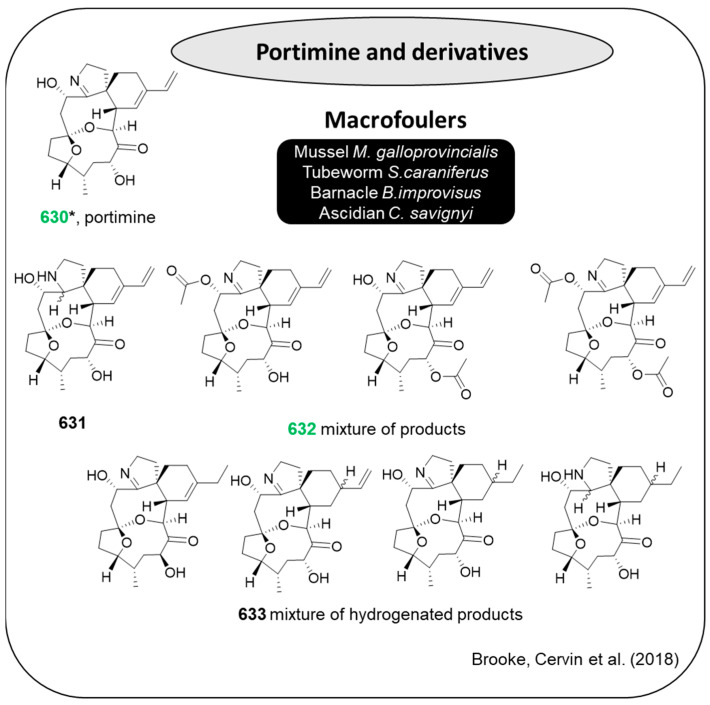
Portimine (**630**) and derivatives **631**–**633** [138]. * Natural compounds; green numbers: most active compounds.

Portimine (**630**, Figure 32), a compound isolated from the marine benthic dinoflagellate *Vulcanodinium rugosum* collected from Northland, New Zealand, is a polycyclic ether toxin containing a cyclic imine moiety, which consists of an unprecedented five-membered ring with a spiro-link to a cyclohexene ring [137]. A study to assess the structural requirements of the AF activity of portimine (**630**), a benthic microalga-derived natural product, included the synthesis of eight semisynthetic analogs (**631**–**633**, Figure 32) [138].

Natural product **630** was tested against the ascidian *Ciona savignyi*, the mussel *M. galloprovincialis*, the tubeworm *S. caraniferus*, and barnacle *A. improvisus*, whereas synthetic compounds **631**–**633** were only tested against the ascidian *C. savignyi* [138]. Compound **630** showed potent (nanomolar scale) and broad-spectrum AF activity. Cyclic amine **631** (EC_50_ = 11.6 ng·mL^−1^) had a ~200-fold lower potency than portimine **630** (EC_50_ = 0.06 ng·mL^−1^) against the settlement of the cyprid *C. savignyi*; however, compound **631** is still more active than some commercial synthetic biocides tested in the same bioassay. For the mixture of hydrogenated products (**633**), a ~100-fold decrease in potency (EC_50_ = 7.1 ng·mL^−1^) was observed compared with compound **630**, which might indicate that the diene moiety is also important for AF activity against the settlement of the ascidian *C. savignyi*. Overall, the cyclic imine and diene moieties increased the nanomolar potency of **630**. Acetylation of portimine was well tolerated, as the mixture of portimine monoacetates and diacetate (**632**, EC_50_ = 1.0 ng·mL^−1^) was as potent as portimine (**630**), suggesting that the α-hydroxy ketone of **630** is not essential to AF activity [138].

### 2.22. Pyridinium Salts

3-Alkylpyridinium (3-AP) compounds occur mainly in marine sponges. More than 70 structurally distinct compounds were isolated from marine sponges belonging to the order Haplosclerida. Most isolated 3-AP compounds are monomeric structures differing in length, saturation, branching, and termination of the alkyl chains. These molecules exhibit valuable potential as novel pharmaceuticals with a large spectrum of applications such as oligonucleotide and/or gene therapy, treatment of several diseases due to various biological activities, and they can be used as active compounds in AF paints [139].

**Figure 33:**
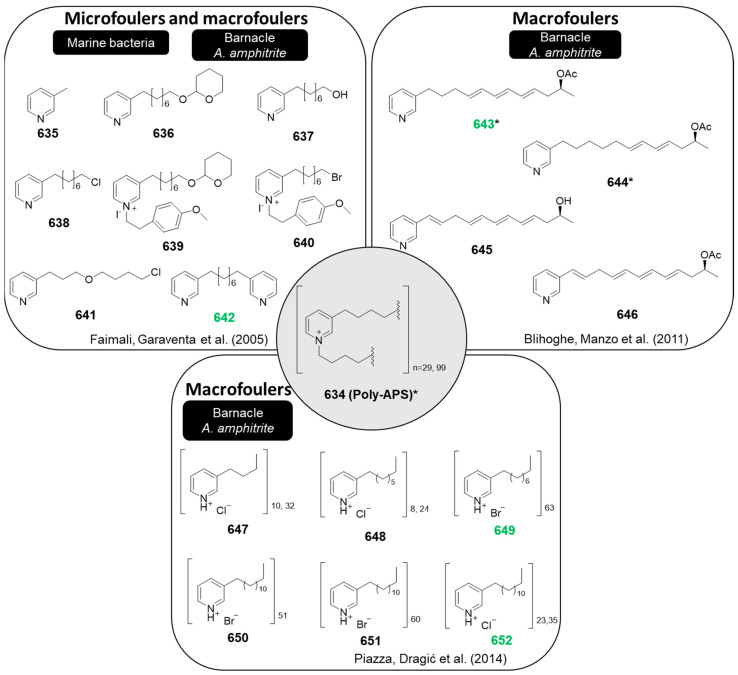
Poly-APS (**634**) and synthetic derivatives **635**–**642** and **645**–**652** [140,141,142]. * Natural products; green numbers: most active compounds.

From 2003 to 2014, 19 synthetic pyridinium salts with AF activity were reported in the literature [140,141,142]. Polymeric 3-alkylpyridinium salts (poly-APS, **634**, Figure 33) are a natural mixture of compounds isolated from the Mediterranean sponge *Reniera sarai* (Haliclonidae) that displayed potent AF activity, both in terms of settlement inhibition of *A. amphitrite* larvae and inhibition of natural marine biofilm formation [143]. The AF efficacy of poly-APS (**634**) was similar to that of CuSO_4_, with the advantage of having a non-toxic mechanism towards the barnacle larvae [143].

Synthetic 3-AP compounds with a defined number of subunits were prepared to understand the relationship between structural features and AF properties. Mono-, di-, and tetrameric 3-octylpyridinium analogs (**635**–**642**, Figure 33) were obtained and the AF activity was tested against the settlement of the larvae of *A. amphitrite* to understand the SAR of the natural poly-APS (**634**) [140].

The absence of AF activity of compound **635** and the corresponding pyridinium salt (not shown), when compared with compounds bearing an octyl chain at position 3 of the pyridine unit (**636**–**640** and **642**), highlighted the importance of the alkyl chain. A significant effect of the length of the alkyl chain was observed by direct comparison of **637** (EC_50_ = 98.26 µM) with a compound with a shorter alkyl chain that did not display any AF activity (structure not shown). The presence of a tetrahydropyranyl group increased the settlement inhibition of compound **636** (EC_50_ = 8.07 µM) when compared with compounds **637** and **638** (EC_50_ = 98.26 and 58.9 µM, respectively). The presence of a positive charge in compound **639** (EC_50_ = 73.40 µM), a compound also bearing a tetrahydropyranyl group, led to a decrease in the settlement inhibition when compared with compound **636** (EC_50_ = 8.07 µM). The potent AF activity observed for compound **642** (EC_50_ = 1.64 µM), higher than the natural compound **634**, confirms the SAR found for compounds **635**–**641**. In contrast to **634**, a therapeutic ratio (LC_50_/EC_50_) of 8.1 was found for compound **642**, which might suggest that cyprid settlement is inhibited by a toxic mechanism [140].

Poly-APS (**634**) and synthetic analogs **635**–**642** were also described to have antibacterial activity against a panel of 24 bacterial strains, including marine isolates associated with immersed surfaces [144]. Compounds **635**–**638** and **641** did not display significant antibacterial activity. In general, the increased antibacterial activity was correlated to the increasing number of pyridinium rings, which is directly related to the molecular weight and the presence of positive charges of the tested compounds. When the bromine of compound **640** was substituted by a tetrahydropyranyl group (compound **639**), a loss in antimicrobial activity was observed [144].

The AF potential of four compounds containing one or more 3-AP moieties (**643**–**646**, Figure 33) was investigated in both anti-settlement activities against cyprids and toxicity against nauplii of the barnacle *A. amphitrite* [141]. Compounds **643** and **644** were isolated from an Antarctic sponge of the genus *Haliclona* sp., while compounds **645** and **646** were obtained by synthesis. All compounds showed promising activity and generally low toxicity. Compound **643** (EC_50_ = 0.28 µg·mL^−1^) was almost as active as the natural Poly-APS **634** (EC_50_ = 0.19 µg·mL^−1^) [141].

Six synthetic polymer analogs of poly-APS (**647**–**652**, Figure 33) were synthesized, and their AF activity was explored against the settlement of the cyprids of *A. amphitrite* [142]. From the synthetic poly-APS **647**–**652**, compound **649** (EC_50_ = 0.026 µM) inhibited the settlement of cyprids most effectively. No significant toxicity towards *A. amphitrite* nauplii was observed, and a therapeutic ratio higher than the natural Poly-APS **634** was found for compound **649** (LC_50_/EC_50_ = 111 and 158, respectively), which suggests an even lower environmental risk. In contrast, compound **652**, the second most potent synthetic polymer (EC_50_ = 0.146 µM), exhibited toxicity towards *A. amphitrite* nauplii. No relation between the AF activity and the length of the alkyl chains, the degree of polymerization, and the nature of their counter ions (chloride or bromide) of synthetic poly-APS was found [142].

In contrast to synthetic polymer analogs of poly-APS (**647**–**652**), synthetic 3-alkylpyridinium compounds (**635**–**646**) possess much simpler structures, and given the potential application of this class of compounds as AF agents, larger amounts of these molecules are expected to be easily obtained. Regarding the mechanism of action, 3-APS show surfactant properties that enables the disruption or solubilization of the fouling organisms’ cell membrane. Moreover, 3-APS were shown to inhibit AChE activity, acting as cholinergic antagonists, blocking neurotransmisson and decreasing the ability of settlement of fouling organisms [46].

### 2.23. (+)-Sclerotiorin and Derivatives

**Figure 34:**
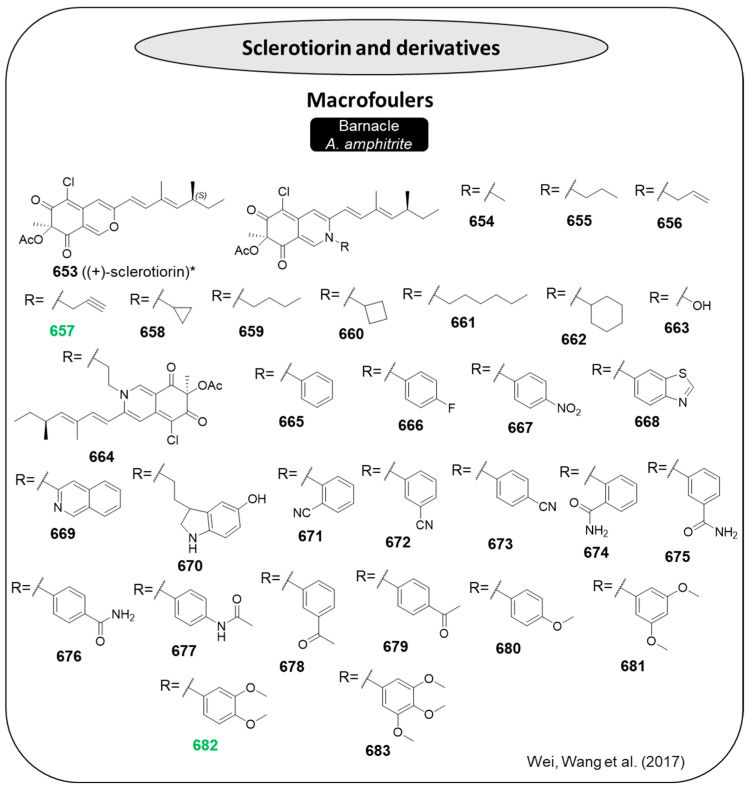
(+)-Sclerotiorin (**653**) and derivatives **654**–**683** [145]. * Natural products; green numbers: most active compounds.

(+)-Sclerotiorin (**653**, Figure 34), a natural product first isolated from *Penicillium sclerotiorum* (from a piece of fresh tissue from the inner part of the gorgonian coral A. ocbracea (GXWZ-33), collected from the Weizhou coral reef in the South China Sea), was part of a program to discover more potent AF compounds against the settlement of *A. amphitrite* (EC_50_ = 5.6 µg·mL^−1^) [145]. To understand the structural requirements for AF activity, a series of 30 new sclerotiorin amine derivatives (**654**–**683**, Figure 34) was obtained and evaluated against the larval settlement of the barnacle *A. amphitrite* [145]. All the tested compounds, except **658**–**660**, **664**, and **680**, could inhibit the larval settlement of the barnacle *A. amphitrite*. In general, amine derivatives showed potent AF activities and were even more potent than the natural product **653**. Interestingly, most of the aromatic amino-derivatives (**665**–**669**, **671**–**673**, **675**, **677**–**679**, and **681**–**683**) showed strong AF activity; however, only two aliphatic amino-derivatives (**657** and **662**) were active. Compounds **657** and **682**, which were the most potent (EC_50_ = 0.94 and 2.6 µg·mL^−1^, respectively), showed high therapeutic ratios (LC_50_/EC_50_ > 53.2 and 19.5, respectively), suggesting that they are low toxicity AF candidates that inhibit the larval settlement of *A. amphitrite* and are a good starting point for the development of new potential AF agents [145].

### 2.24. Sesquiterpenes

**Figure 35:**
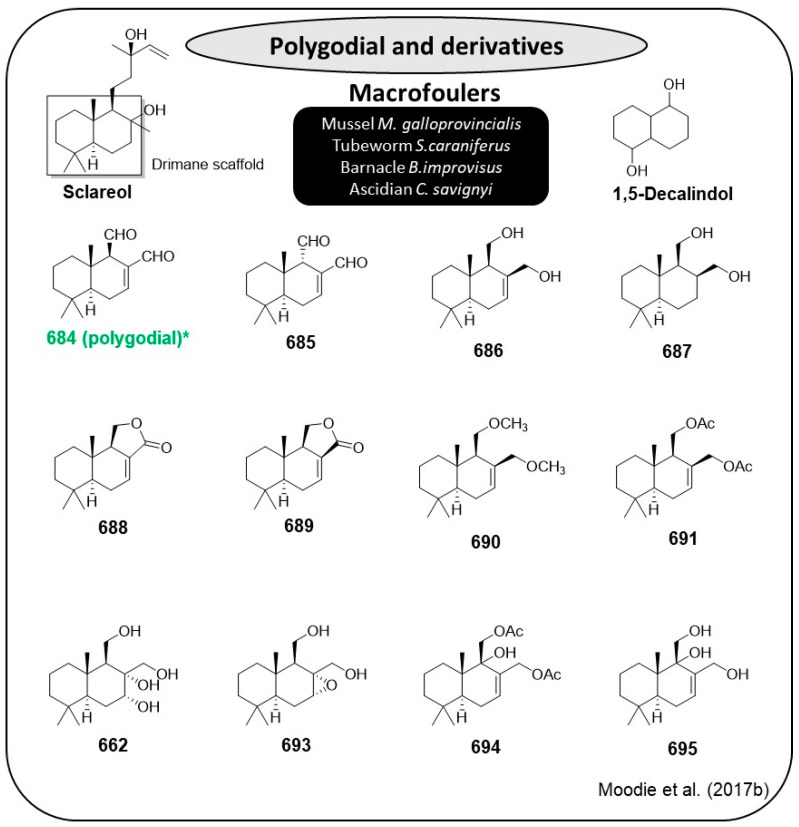
Polygodial (**684**) and derivatives **685**–**695** [147]. * Natural products; green numbers: most active compounds. Note: other sesquiterpenes can be found in Figure 10 and Figure 21.

Polygodial (**684**, Figure 35), isolated from marine sponges and nudribranchs, is a drimane sesquiterpene that acts as a nonionic surfactant to disrupt the cell membrane of microorganisms [146,147]. This compound shows powerful AF properties against the marine macrofoulers *C. savignyi* (EC_50_ = 3.4 ng·mL^−1^), *Spirobranchus caraniferus* (EC_50_ = 5.2 ng·mL^−1^), and *M. galloprovincialis* (EC_50_ = 2.9 ng·mL^−1^) [148]. Furthermore, compound **684** was shown to display low toxicity toward higher organisms [149,150]. A library of 11 polygodial analogs was prepared by semi-synthesis, intending to investigate the SAR of the drimane scaffold (**685**–**695**, Figure 35) [147]. All compounds share the bicyclic drimane, with the differences lying mainly within the degree of substitution and oxidation [147]. This compound class was only weakly active against microfoulers. In contrast, compound **684** and its synthetic analogs (**685**–**695**) showed powerful AF activities against macrofouling species: the ascidian *C. savignyi* (EC_50_ = 0.004–1.0 µg·mL^−1^) and the barnacle *A. improvisus* (EC_50_ = 0.1–1.5 µg·mL^−1^). Nevertheless, none of the synthetic analogs were more potent than the natural compound **684** [147]. The fact that the diterpenoid reference compound sclareol (Figure 35) was also active, while 1,5-decalindol (Figure 35) did not display significant inhibition at the concentrations tested, suggests that the drimane scaffold (Figure 35) may be essential to generate the AF activity [147]. The observed bioactivity profile of the drimane-type compounds suggested that the mode of action is probably dictated by differing mechanisms depending on target species, rather than a general biocidal effect. General surfactant properties appear partly responsible for observed activity against macrofoulers, but there was evidence that specific molecular targets also exist; elucidating these targets should be a priority for future research in this area [147].

Regarding the mechanism of actions, it deserves to be mentioned that it is speculated that polygodial can interfere with heat shock protein 90 (HSP-90), which can lead to the inhibition of larval metamorphosis. In concrete, polygodial showed inhibit larval metamorphosis in ascidian *C. savignyi* larvae with IC_99_ (99% inhibition concentration) of 0.003 µg·mL^−1^ [151].

**Figure 36:**
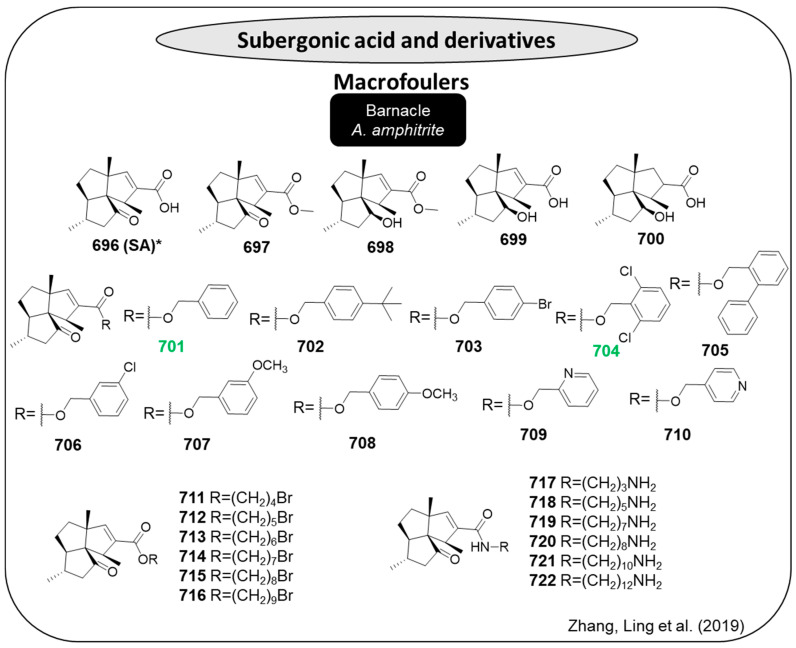
Subergonic acid (**696**) and derivatives **697**–**722** [152]. * Natural products; green numbers: most active compounds.

**Scheme 16:**
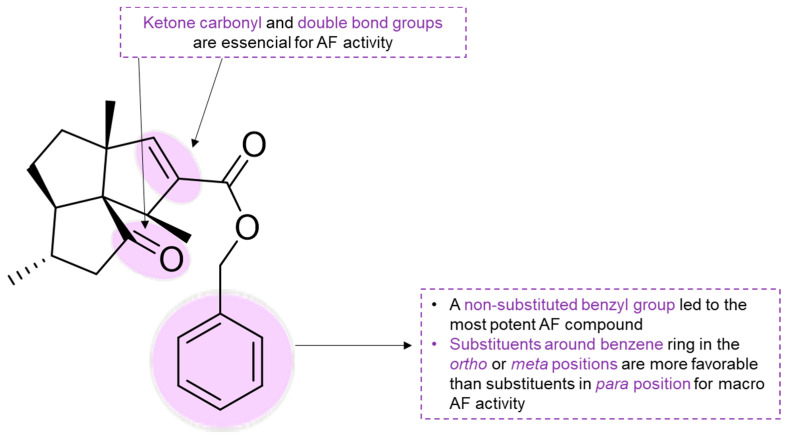
SAR for subergonic acid derivatives.

A SAR study of the tricyclic sesquiterpene subergorgic acid (**696**) was performed firstly by isolating the compound from the gorgonian coral *S. suberosa* and then by the synthesis of diverse derivatives (**697**–**722**, Figure 36) [152]. The good AF activity in addition to the high abundancy in *S. suberosa* makes compound **696** (EC_50_ = 1.25 µg·mL^−1^) a good lead compound to perform SAR studies aiming to identify more potent AF compounds. Compounds **696**–**722** were tested against the settlement of the barnacle *A. amphitrite*. Esterification of subgorgic acid (**696**) led to derivative **697** (EC_50_ = 3.16 µg·mL^−1^), which maintained most of the AF activity, suggesting that the acid group is not essential for the AF activity. Surprisingly, both the ketone carbonyl and the double bond groups were essential for the AF effect, as seen for the loss of AF activity of compounds **699** and **700** (EC_50_ > 25 µg·mL^−1^ for both compounds) [152]. Following this, two series of subgorgic acid (**696**) derivatives, one containing benzyl functional groups (**701**–**710**) and the other involving the methylene chain of various lengths (**711**–**722**), were obtained to develop a more detailed SAR [152]. All benzyl esters exhibited good AF effects against the settlement of *A. amphitrite*. The most potent compound (**701**, EC_50_ = 0.30 µg·mL^−1^) containing a non-substituted benzyl group showed that substituents around the benzene ring decreased the AF activity as well as steric effects [152]. The position of the substituent on the benzene ring might influence the AF effect, with substituents in the ortho or meta position being more favorable than substituents in the *para*’position. Bromoalkane derivatives **711**–**716** of various lengths did not exhibit better or comparable AF activity when compared with subgorgic acid (**696**), and the greater the length, the lower the AF activity. For the series of aminoalkanes derivatives of subgorgic acid with various lengths (**717**–**722**), all the compounds were active, although less active than subgorgic acid (**696**) [152]. Interestingly, the opposite trend of compounds **711**–**716** was found for compounds **717**–**722** and the compound with the longest methylene chain, derivative **722** (EC_50_ = 4.5 µg·mL^−1^), showed the best AF activity. The differences observed were attributed to the fact that the presence of -NH_2_ is better compared to -Br [152]. Scheme 16 summarizes the SAR for subergonic acid and derivatives.

### 2.25. Stilbene Derivatives

**Figure 37:**
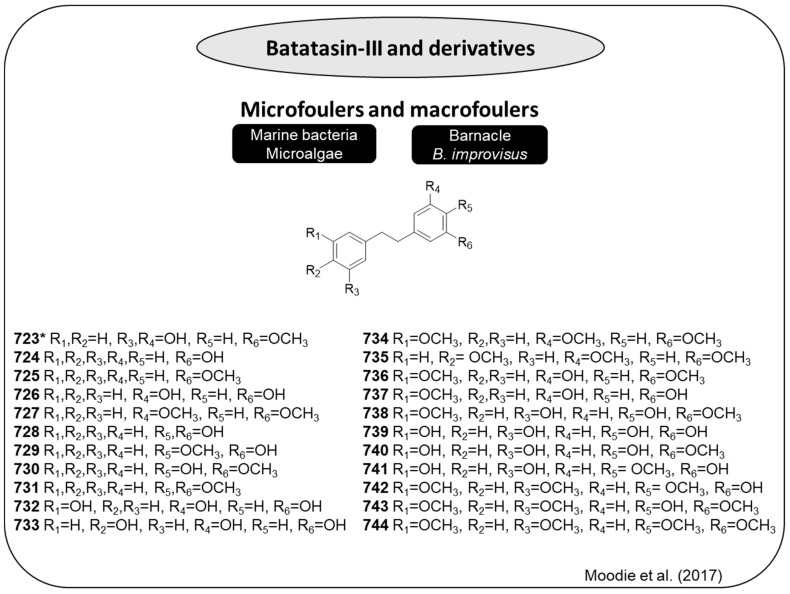
Batatasin-III (**723**) and derivatives **724**–**744** [153]. * Natural products.

Batatasin-III (**723**, Figure 37), a stilbene derivative, is an allelopathic phytochemical which is produced by terrestrial plants to prevent the establishment of other plant species that compete for space or resources [153]. To establish if the allelopathic effect of batatasin-III (**723**) is transferable to a marine setting, Moodie and colleagues prepared a library of synthetic dihydrostilbenes with various substitution patterns (**724**–**744**, Figure 37) [153]. The AF activity of the synthesized compounds was evaluated against ten different marine bacterial strains, four microalgal strains, and the barnacle *A. improvisus*. This library was also designed to investigate the contributions from both hydroxy and methoxy substituents commonly found in naturally occurring stilbenes, as well as to evaluate the impact of the physicochemical properties on the biological activity [153]. According to this study, apart from compounds **728**, **730**, and **742**, all the prepared dihydrostilbenes were active against marine microalgae at low concentrations displaying low MIC values at sub-μg·mL^−1^ concentrations, but, in general, these compounds were not inhibitors of adhesion or growth of marine bacteria. Compounds **725**, **731**, and **724** inhibited the settlement of *A. improvisus* without mortality, indicating a non-toxic mechanism [153]. In contrast, compounds **724**, **729**, and **742** killed all the cyprids, implying a different mechanism of action from the compounds mentioned above. It was also possible to observe that the physicochemical properties influenced the settlement inhibition of the analyzed compounds. The poorly active compounds **733**, **739**, **740**, and **741** are hydrophilic, containing a minimum of three hydroxy groups. So, there is a relationship between increased polarity and hydrogen bond capacity and a decrease in inhibitory effect in barnacles [153]. Ascidians are a challenging macrofouling species to manage in biofouling, and compounds displaying a high inhibitory activity against *A. improvisus* were selected for evaluation against *C. savignyi*. All the six compounds selected (**723**, **727**, **729**, **731**, **742**, and **744)** inhibited *C. savignyi* larval metamorphosis at low concentrations [153]. Some SAR was observed, although the exact mechanism of action is not clear. Nonetheless, dihydrostilbenes were disclosed to be a starting point to develop AF agents.

Another study combined the natural dihydrostilbene scaffold with an oxime moiety [154]. This series of hybrid compounds was tested against the settlement of barnacle *A. improvises* larvae and the adhesion and growth of ten marine bacterial and four microalgal species [154]. Most of the tested compounds were highly efficient in terms of AF performance, although they did not appear to perform better over the previously reported batatasin library (**724**–**744**) [153].

### 2.26. Structure-Diverse Sulfated Derivatives

**Figure 38:**
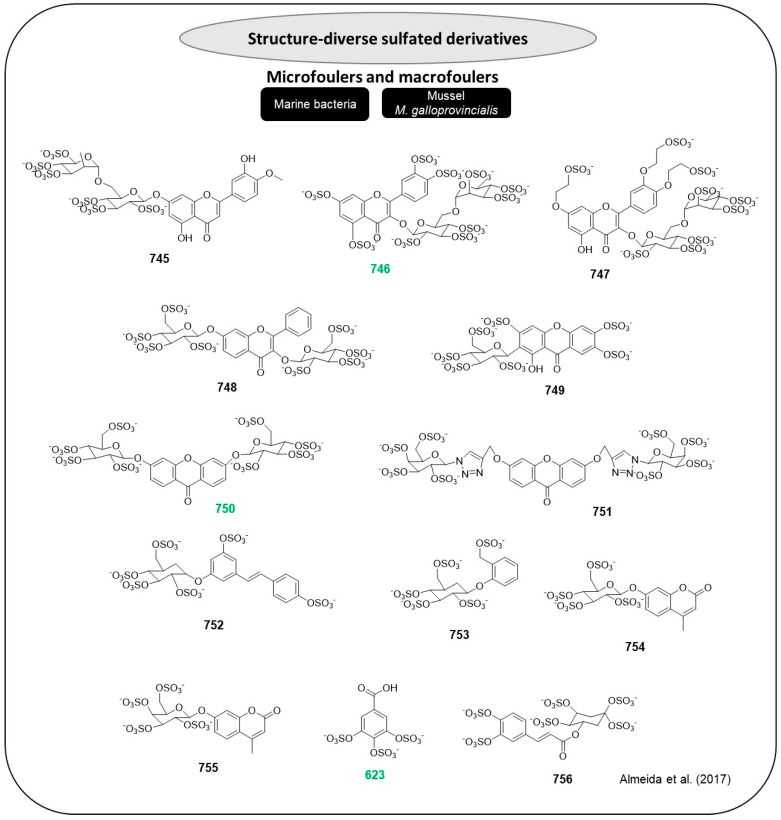
Synthetic sulfates of flavonoids, xanthones, coumarins, and phenolic acids, **745**–**756** [65]. Green numbers: most active compounds.

Sulfation is used in nature as a metabolic strategy to avoid toxicity, and based on this consideration, a library of 13 sulfated NPs derivatives (**745**–**756**, Figure 38) was developed and investigated for AF activities [65]. Compounds **746**, **750**, and **623** showed a significant inhibitory effects against the settlement of *M. galloprovincialis* larvae with EC_50_ values of 22.59, 23.19, and 17.65 µM, respectively, without toxicity against this target organism. To find some clues about their mechanism of action, the activities of AChE and tyrosinase in the presence of compounds **746**, **750**, and **623** were studied, although none of them were able to inhibit these two enzymes implicated in the adhesion process of mussels [65]. No antibacterial activity against the growth of biofilm-forming marine bacteria (*C. marina*, *V. harveyi*, *P. atlantica*, and *H. aquamarina)* was observed for compounds **745**–**756**. Two ecotoxicity assays were performed to gain insights about the potential toxicity of the compounds against non-target organisms, specifically, *A. salina* and *Vibrio fischeri*. None of the compounds showed toxicity in the non-target organisms [65]. It is important to notice that none of the non-sulfated compounds showed AF activity, suggesting that sulfate groups are responsible for the AF activity displayed by these compounds. Some SAR could be observed for this library of sulfated compounds, namely, the nature of the scaffold (compound **750**, which showed some activity compared to compound **751**, a triazole-linked xanthone with no activity). For the flavonoids **745**–**748**, sulfate groups at positions 5, 7, 3′, and 4′ were important to the slight anti-settlement activity displayed by compound **746** (which was only active at the highest concentration tested). Additionally, the degree of acidity of the carboxylic group seems to influence the AF activity, when comparing compound **623** with an arylcarboxy group and compound **756** with an alkylcarboxy group [65]. Compound **623** was selected for further studies concerning its immobilization in polymeric coatings [155]. Additionally, the synthetic method to obtain compound **623** was optimized, increasing its scale-up potential, an important aspect when considering the development of a new AF agent [155]. Compound **623** was directly incorporated and chemically immobilized in two representative PDMS and PU-based marine coatings and two commercial acrylic and room-temperature-vulcanizing polydimethylsiloxane (RTV-PDMS)-based non-marine coatings. Chemical immobilization consisted of the pre-functionalization of compound **623** with TZA [155]. Leaching studies proved that the formulation with pre-functionalized compound **623** released less amounts of compound **623**, compared with the formulation containing compound **623** directly incorporated, in both PDMS and PU-based coatings. PU, acrylic, and RTV-PDMS-based coatings formulated with pre-functionalized compound **623** maintained their AF activity against the settlement of *M. galloprovincialis* larvae [155]. These results indicate that compound **623** has great potential to be developed as an active ingredient in AF paints.

### 2.27. Xanthones

**Figure 39:**
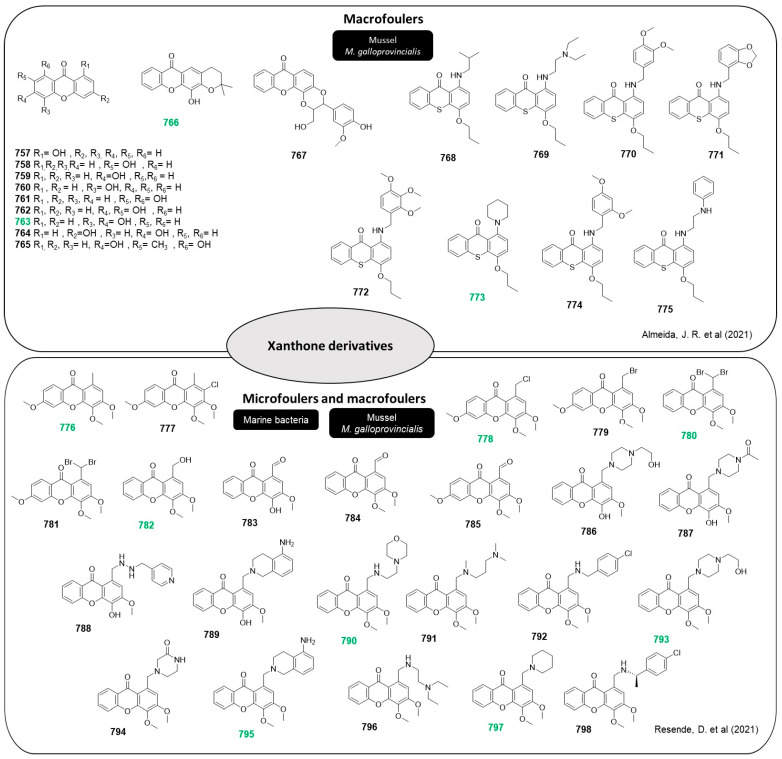
Xanthone derivatives tested for antisettlement activity toward the macrofouling species *M. galloprovincialis* [157,158]. Green number: most active compounds.

Xanthone is a heterocyclic molecule, with a dibenzo-*γ*-pirone scaffold, known as 9*H*-xanthen-9-one. The designation of rings A and B is congruent with the biosynthetic pathways for the compounds from higher plants, A-ring (carbons 1–4) being acetate-derived and the shikimic acid pathway gives B-ring (carbons 5–8); the other carbon atoms are numbered according to IUPAC [156]. The first report of a synthetic xanthone as an AF agent was described in the previous structure-diverse sulfated derivatives section, compound **750** [155]. Following this, a library of 19 synthetic xanthones (**757**–**775**, Figure 39) was investigated for their AF potential, specifically through the anti-settlement activity toward the macrofouling species *M. galloprovincialis*, the study of the potential modulation of AChE and tyrosinase, enzymes with a role in adhesive processes, and by the differential analysis of the proteome of *M. galloprovincialis* plantigrade larvae after exposure [157]. Additionally, QSAR studies were performed to understand the structural requirements to achieve AF activity. General marine ecotoxicity against a non-target organism, *A. salina*, was also studied [157].

Xanthones **760**, **761**, **763**, **766**, **771**, and **773** were able to inhibit the settlement of *M. galloprovincialis* larvae (EC_50_ = 3.53–28.60 µM) without exerting toxicity against this target species (LC_50_ > 500 µM and LC_50_/EC_50_ = 17.48–141.64). The QSAR model developed revealed that the complexity of the molecule, the electronic charge distribution, and inter-atomic distances influence their AF activity [157]. Within this series, compounds **763**, **766**, and **773** were the most potent by inhibiting mussel larvae settlement with EC_50_ values of 11.53 µM, 4.60 µM, and 3.53 µM, respectively. It deserves to be highlighted that while compound **761**, **763**, **766**, **771**, and **773** showed some toxic effects (mortality > 10%), at 50 and 25 µM to the nauplii of *A. salina*, xanthones **763** and **766** demonstrated no toxicity to this non-target species, which makes these two compounds better candidates for their inclusion in AF coatings [157].

Proteomic studies were realized to provide molecular insights on the AF properties of selected compounds. The proteome of competent *M. galloprovincialis* plantigrade larvae was analyzed in response to the three most potent compounds (**763**, **766**, and **773**) [157]. Compound **763** showed an impact on the expression of proteins involved in cytoskeleton formation, cell-redox status, and chaperone-mediated regulation. Moreover, compound **763** may be related to the alteration in the abundance mussel collagen proteins (PreCols), specific to the byssal threads, that play an important role in elasticity, resistance to tension, and shock absorption [157]. Remarkably, the inhibition of larvae adhesion of compound **766** may be explained by the abrupt decrease of two putative proximal thread matrix proteins (TMPs), proteins that are localized in byssal threads to provide them viscoelasticity [157]. Compound **773** caused an alteration in the abundance of five proteins which are involved in major metabolic processes such as glycolysis/gluconeogenesis, gene translation, lysosomal protein degradation, or are constituents of the cytoskeleton [157].

In what concerns SAR, it was later shown that while the presence of hydroxyl groups at positions 3 and 4 was not crucial for activity, the presence of 3 and 4 oxygenated groups were important for the antisettlement activity on mussel larvae [158].

The xanthone derivative (3,4-dihydroxyxanthone) **763** (Figure 39) was immobilized in representative coating components of marine PU-based coatings using a 4,4 diphenyl diisocyanate-monomeric (MDI) crosslinker, allowing its chemical binding with the polymeric matrix of the coating. The water solubility of compound **763** is similar to both booster biocides irgarol 1051^®^ (6.0–7.0 mg·L^−1^) and sea nine 211^®^ (4.7–6.5 mg·L^−1^), demonstrating to be a suitable AF agent to be incorporated in marine coatings. Laboratory tests showed that compound **763** maintained its AF activity against the settlement of *M. galloprovincialis* after being chemically immobilized following this crosslinking strategy, and its release from the formulation was lower than the conventional releasing systems of PU-based marine coatings [158].

**Scheme 17:**
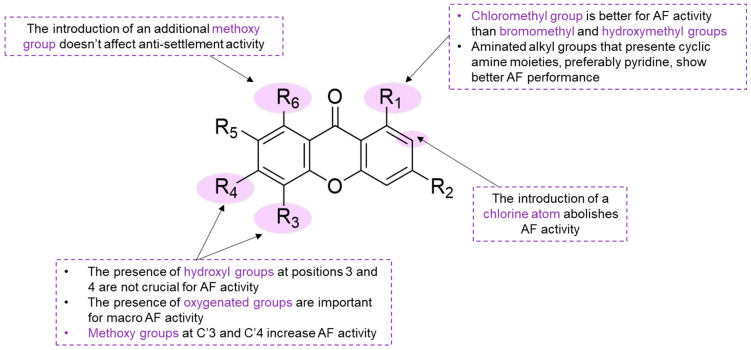
SAR for xanthone derivatives.

In another study, 24 synthetic xanthones with three substitution patterns—1,3,4-trisubstituted xanthones, 1,3,4,6-tetrasubstituted xanthones, and 1,2,3,4,6-pentasubstituted xanthone—were analyzed [159]. To evaluate the AF potential of these compounds, anti-settlement bioassays against the larvae of *M. galloproviancialis* and antibacterial assays against *C. marina*, *V. harveyi*, *P. atlantica*, *H. aquamarina,* and *R. litoralis* were performed. None of tested compounds were considered antibacterial agents against these marine biofilm-forming strains because they did not inhibit significantly (>30%) the growth of bacteria. Moreover, promising AF compounds were submitted to a marine ecotoxicity evaluation using *A. salina* and proteomic studies were performed with the purpose to identify putative AF molecular targets. Particularly, compounds **776**, **778**, **780**, **782 790**, **793**, **795**, and **797** presented highly significant anti-settlement responses. In what concerns SAR of xanthone derivatives, comparing compounds **776**, **781**, and **785** with **780**, and **784**, it appears that the introduction of an additional methoxy group at position **780** does not affect the anti-settlement activity. The introduction of a chlorine atom in compound **777** nullified the pronounced anti-settlement activity of compound **776**. Comparing compounds **784**, **795**, and **793** with compounds **783**, **789**, and **786**, respectively, it is possible to infer that the presence of methoxy groups at positions 3 and 4 increase the anti-settlement effect, while the presence of a hydroxy group at position 4 and a methoxy group at position 3 has not so much significant impact in the *M. galloprovincialis* larvae settlement inhibition [159]. In respect to the variety of substituents at C-1, the chloromethyl group in compound **778** increased the anti-settlement activity if compared with the bromomethyl group in xanthone **779** and the hydroxymethyl group in xanthone **782**. Relative to the aminated alkyl groups in xanthones **790**–**798**, derivatives **790**, **793**, **797**, and **795** that present cyclic amine moieties showed better AF performance [159]. Xanthones **776**, **778**, **780**, **782**, **790**, **793**, **795**, and **797** were the most promising candidates as AF agents, but is important to emphasize that compounds **795** and **797**, both containing pyridine moiety at C-1, were considered the most effective larval settlement inhibitors with EC_50_ levels of 1.26 µg·mL^−1^ and 3.03 µg·mL^−1^, respectively. It is valuable to note that, compound **773** containing a pyridine group was related to the enhancement of anti-settlement activity [159]. According to the SAR analysis, it is possible to infer the importance of a 3,4-dimethoxy substitution in the xanthone core with an additional cyclic amine moiety, preferably a pyridine to hit optimization from 3,4-dihydroxyxanthone (compound **763**). Scheme 17 summarizes the SAR for xanthone derivatives. Preliminary ecotoxicity evaluation in *A. salina* showed that xanthones **778**, **780**, **782**, **790**, **793**, **795**, and **797** may have lower toxicity to the environment than some of the current biocides in use, as in the case of econea^®^ [159]. Regarding the mechanism of action, proteomic studies indicated that xanthones **795** and **797** show similar molecular targets and impact similar molecular processes, which include diverse miosines from pedal refractor muscle. In conclusion, xanthones **795** and **797** emerged as hit AF compounds revealing higher anti-settlement effectiveness against *M. galloprovincialis* larvae compared with **763** [159].

### 2.28. Zosteric Acid and Other Cinnamic Acid Derivatives

**Figure 40:**
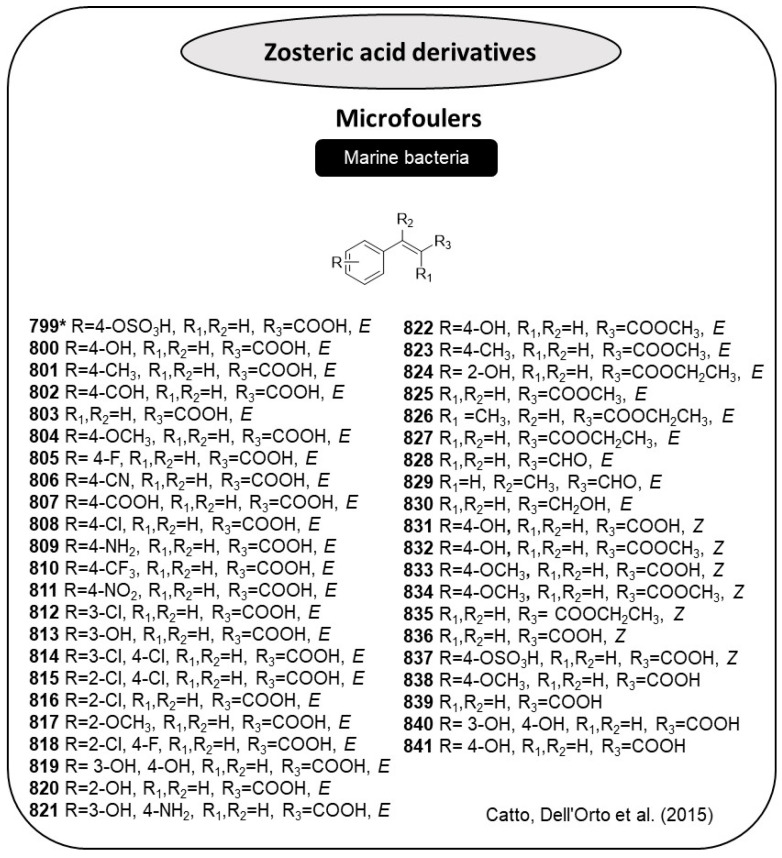
Zosteric acid (**799**) and derivatives (**800**–**841**) [161]. * Natural products.

Zosteric acid or *p*-(sulfoxy) cinnamic acid (ZA, **799**, Figure 40) is a specialized metabolite produced by the seagrass *Zostera marina* capable of preventing biofilm formation [160]. This natural product is one of the most studied natural AF compound [161]. To identify important structural determinants for the ZA antibiofilm activity, a library of ZA analogs with improved antibiofilm activity was developed (**800**–**841**, Figure 40). The *E. coli* maximum specific growth rates in the presence of several concentrations of each compound were calculated and compared to bacterial growth in the absence of compound. Compounds **800**–**805**, **807**–**808**, **812**–**814**, **819**, **820**, **822**, **825**, **827**, and **840** had no biological activity at low concentrations, but showed antibiofilm activity at middle concentrations and induced biocidal effect at the maximum concentration. ZA and compounds **815**, **816**, **821**, and **832**–**835** had a biocidal effect at middle and higher concentrations, while at low concentrations, these compounds promoted biofilm formation [161]. A peculiar trend was observed for compounds **831**, **836**, and **837**: compound **830** showed antibiofilm activity at the lowest concentration, no biological activity at middle concentrations, and biocidal effect at the highest concentration; compound **836** promoted biofilm formation at 0.183, 1.83, and 183 μM, inhibited biofilm formation at 18.3 μM, and induced a biocidal effect at the highest concentration; finally, compound **837** promoted biofilm formation at all tested concentrations except for the 183 μM, where no biological activity effect was observed [161].

**Scheme 18:**
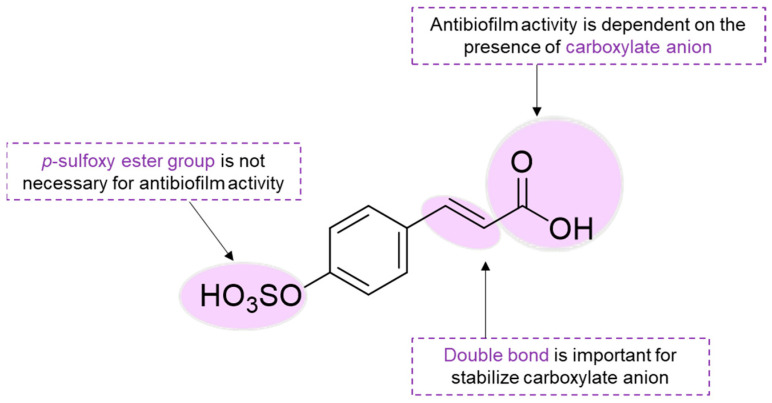
SAR for zosteric acid derivatives.

According to the substitutions performed, the *p*-sulfoxy ester group is not necessary for the antibiofilm activity; however, the cinnamic acid scaffold is responsible for the antibiofilm activity; the antibiofilm activity of ZA derivatives is dependent on the presence of a carboxylic acid and consequently on its hydrogen-donating ability, which is influenced by the type of groups present in the phenyl ring; the conjugated aromatic system is essential for the antibiofilm activity of ZA and its analogs, since the presence of the double bond stabilizes the carboxylate anion [160]. Scheme 18 summarizes the SAR for zosteric acid derivatives. It was shown that 500 mg·L^−1^ of the sodium salt of zosteric acid causes alterations in the whole proteomic signature of *E. coli*, including stress-associated, motility-related, quorum-sensing-associated (LuxS enzyme), and metabolism/biosynthesis-related proteins [162].

Interestingly, it was speculated that the AF effect of zosteric acid on algae *Ulva* sp. could be due to the combination with the adhesive secreted by spores, or the attachment to the surface of the glass substrate. The strong hydrophilicity of the sulfonic acid group prevented or reduced the exclusion of the water between the adhesive and the substratum, making it difficult to form the adhesive-substratum interface [135].

**Figure 41:**
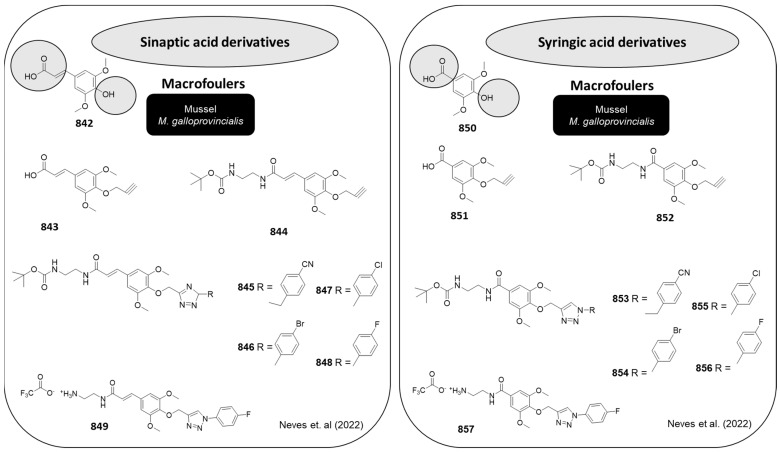
Sinapic acid (**842**) derivatives (**843**–**849**) and syringic acid (**850**) derivatives (**851**–**857**) [36]. Note: only compound **856** was able to reduce the settlement of *M. galloprovincialis* larvae.

A series of sinapic acid (**842**) derivatives was synthesized (**843**–**849**, Figure 41) by click chemistry [36]. For SAR purposes, similar derivatives of syringic acid (**850**) were also obtained (**851**–**857**, Figure 41). However, only the syringic acid derivative **856** was active, decreasing the settlement of *M. galloprovincialis* larvae with an EC_50_ of 12.91 μM, a lower value than the previous obtained lead compound gallic acid persulfate (EC_50_ = 18 μM, **623**, Figure 31, Section 2.20). Regarding the mechanism of action, the compound **856** was tested for the inhibition of AChE and tyrosinase activities, but the compound did not affect these two pathways [36].

## 3. Computational Chemistry: An Approach to Search for Novel Antifoulants

**Figure 42:**
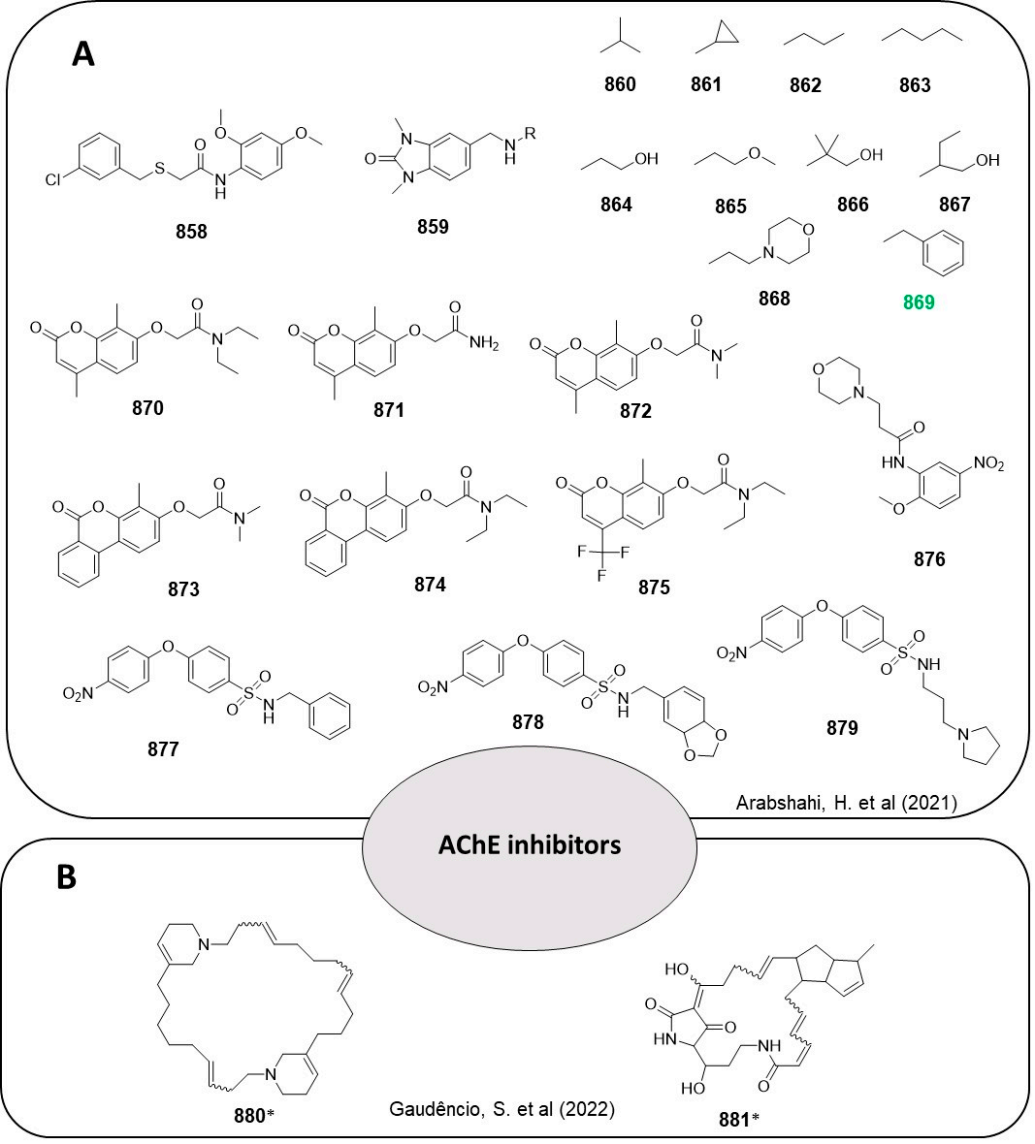
(**A**) Structures of analogues designed and selected in silico for the in vitro screen and for micro- and macrofouling evaluation; (**B**) haliclamid B (**880**) and cylindramide (**881**) [165,166]. * Natural products; green: most active compounds.

Computational chemistry is a valuable tool when it comes to uncovering putative molecular targets and predicting pharmacophores [163]. Throughout this review, we realized its importance in the chapters of: butanolide [103] (Section 2.14), in which the mode of binding was predicted towards cetyl-CoA acetyltransferase 1 of *A. amphitrite* and acyl-CoA dehydrogenase of *B. neritina*; anthraquinone derivatives (Section 2.3), in which a molecular docking was used to study the interaction of these compounds with the LuxP protein involved in quorum-sensing signaling [39], with prediction of anthraquinone-related pharmacophore [40]; chalcone and flavonoids [60,64] (Section 2.8); and xanthone derivatives [157] (Section 2.27), in which QSAR studies allowed for concluding the most important molecular characteristics that confer AF activity to the compounds. In this way, in silico approaches are a crucial part of AF discovery to better design promising AF compounds, comprising the molecular targets to prevent organism settlement. Particularly, AChE is a known AF molecular target, impairing neuronal functions [164]. In this sense, to probe the association between AChE inhibition and AF activity, rational in silico design, virtual screening for binding in an AChE homology model, and evaluation of AChE inhibition of marine larvae in vitro assays were undertaken [165]. The most promising compounds (**858**–**879**; Figure 42) were used to choose commercially available structural analogs with ClogP < 4 (selection criterion) that were rescreened experimentally to determine the binding affinity to AChE. Moreover, the AF activity of selected compounds against both microfouling bacteria, microalgae and tunicate *C. savignyi*, an invasive macrofouler that is problematic in scenarios including ship hull fouling [165], was observed.

Of the remaining compounds, five displayed either weak (IC_50_ > 400 uM, compounds **858** and **859**) or moderate inhibitory activity (IC_50_ 10–100 µM, compounds **870**, **876**, and **877**). Analogs of **859**, **870**, and **877** were commercially available. Several analogs of **859** with structural diversity in the *N*-alkyl chain were included in the second screen. Only analog **869** showed strong cholinesterase inhibition with potent inhibitory activity against electric eel (ee)AChE of 9.44 ± 0.85 µM. Docking predictions demonstrated that compound **869** had a different predicted pose from inactive compounds, predicting a hydrogen bonding interaction with SER124 and Pi stacking interactions with TRP83 and TRP331 [165]. Chromanone analogs of compound **870** revealed positive results in the cholinesterase bioassays, except for terminal amide **871** being inactive. Compounds **872** (49 µM) and **875** (3 µM) are suggested to be selective for (ee)AChE, while **870** and **874** may be less selective inhibitors due to the activity against both (ee)AChE and horseradish buthylcholinesterase. Furthermore, **873** displayed a robust activity against all enzymes, illustrating a high general cholinesterase inhibitory profile [165]. Compound **872** showed that the chromene carbonyl moiety features multiple hydrophobic interactions and it has a predicted hydrogen bonding interaction with GLY119. The ester had a predicted interaction with SER124. Compound **874** showed that predicted hydrogen bonding interaction with SER198 may be responsible for the increased activity in the (ee)AChE assay and new activity in the horseradish buthylcholinesterase assay. Of the two analogs of compound **877** evaluated, only **878** showed to be active, but less active than compound **877**, suggesting indication that the difference in activity could be due to the loss of the nitro group [165]. On the one hand, none of the compounds demonstrated to reduce either the settlement or growth of the five microfouling organisms, which can be attributed to the lack of the nervous system of eucaryotes, and also suggests that these compounds are not generally toxic. On the other hand, **869** was the only cholinesterase inhibitor analog that was shown to be a potent inhibitor for the metamorphosis of larvae of the seasquirt *C. savignyi*, with an IC_50_ of 4.60 ± 0.39 μg·mL^−1^ [165].

Recently, Gaudêncio and colleagues (2022) developed a computer-aided design approach to predict new MNPs AF inhibitors of AChE activity [166]. Starting from 14,492 compounds from Encinar’s website and 14 MNPs that are currently in the clinical pipeline, the QSAR classification approach selected 125 MNPs that were screened by molecular docking against the AChE enzyme. Dockthor was used to perform molecular docking of the two best macrocycle hits (cylindramide and haliclamine B), the best non-macrocycle hit (indole derivative), and the positive controls (synoxazolidinone A, synoxazolidinone C, and donepezil) and negative control (phenolic derivative). It was shown that the most probable lead-like AF compounds AChE inhibitors were haliclamine B (**880**), a macrocyclic alkaloid and cylindramide (**881**), a lactame derivative [166].

## 4. Conclusions

Global warming will disrupt the equilibrium of marine environments mainly due to an increase in temperature and input of freshwater. As a consequence, the predicted impact on biofouling organisms is the dominance of species that show UV resistance and low salinity tolerance. In this way, adding to the toxicity of the biocides currently in use, the design of new AF protection alternatives emerges as a priority for the marine coatings industry AF [167].

Over 880 compounds were compiled in this review, most of them obtained in the last six years by synthesis, which reveals that researchers are making efforts to optimize natural compounds to meet the requirements of an eco-friendly AF agent. MNPs were indubitable the models used in the search and development of new effective AF compounds, namely aptamine (**1**) and isoaaptamine (**2**) isolated from the sea hare *Aplysia punctata*, avarol (**40**), from the marine sponge *Dysidea avara*, Moloka’iamine (**66**) and 3,5-dibromo-4-methoxy-b-phenethylamine (**67**) from sponges and ascidians, Hemibastadin-1 (compound **97**) from marine sponges, bromosphaerol (**204**) from the red alga *Sphaerococcus coronopifolius*, isatin (**229**) from a marine bacterium, sesquiterpene 3-isocyanotheonellin (compound **262**) from a nudibranch species, lactones **364**–**372** from different marine *Streptomyces* sp., sesterterpene *γ*-hydroxybutenolides cavernosolide (**402**) and lintenolide A (**403**) from a New Zealand marine sponge, butenolide **371** and geraniol **425** from marine Streptomyces, polyketide 6-pentyl-2*H*-pyrone-2-one (**434**) from the marine-derived fungus *Trichoderma atroviride*, compounds **470** and **471** from red alga Laurencia sp., synoxazolidinones A (**491**) and C (**492**) from the sub-artic ascidian *Synoicum pulmonaria*, iantheline (**493**), phidianidine A (**494**) from aeolid opisthobranch mollusk *Phidiana militaris*, dolastatin 16 (**504**) from the sea hare *Dolabella auricularia*, phenyl ether derivatives **517**–**522** from the fungus *Aspergillus* sp. XS-20090066, barettin (**534**) isolated from the marine sponge *Geodia barretti*, portimine (**630**) from the marine benthic dinoflagellate *Vulcanodinium rugosum*, compounds **643** and **644** from an Antarctic sponge of the genus Haliclona sp., (+)-sclerotiorin (**653**) from the gorgonian coral A. ocbracea (GXWZ-33), polygodial (**684**) from marine sponges and nudribranchs, subergorgic acid (**696**) from the gorgonian coral *S. suberosa*, and Zosteric acid or *p*-(sulfoxy) cinnamic acid (ZA, **799**) produced by the seagrass *Zostera marina.*

The extraction of MNPs is not viable in most of the cases presented here, being that synthetic derivatives are far easier to obtain and many more times potent than their natural counterparts. Regarding SAR, the relevant molecular features for antibiofilm activity were the presence of aromatic rings, such as benzyl and triazole, containing halogen substituents, particularly chlorine and bromine. The presence of phenolic hydroxyl groups, structural modifications such as esterification and amidation, and the presence of primary amines, free NH moieties, benzene, and pyridinium rings, halogen atoms and tryptophan were demonstrated to increase the antibacterial activity. Aromatic rings and amide groups are considered crucial for anti-algal activity and the presence of phenolic groups, chlorine atoms, and the number of phenyl groups improve anti-algal activity.

In general, the presence of hydroxyl groups in the molecular scaffold are related to AF activity in diverse macrofouler organisms, such as mussels (*M. galloprovencialis*, *D. polymorpha*), barnacle cyprids (*B. albicostatus*), and bryozoans (*B. neritina*). For AF activity against the mussel larvae of *M. galloprovencialis*, the presence of methoxyl, hydroxyl, sulfate, and pyridine groups, chlorine atoms, acetamide and cyclic amine moieties, and the degree of acidity of carboxylic acid groups revealed to correlate with AF activity. Molecular features as the presence of bromine atoms, ester, benzyl, and isocyano groups, 2-furanone rings, the introduction of bulky groups (boc), and the consequent increase in lipophilicity appears to improve AF activity against barnacle cyprids of *A. amphitrite.* For barnacle cyprids of *A. improvisus*, the oxime function, guanidine moieties, bromine atoms, and non-polar substituents electronically similar to bromine, the stereochemistry of double bounds, the length of alkyl side chains, and the 1,2,4-oxadiazole ring are the molecular features that contribute more to the AF activity of compounds. Regarding the ascidian *C. savignyi*, the presence of non-cyclic amines and diene moieties increase AF activity. To inhibit the settlement of the bryozoan *B. neritina*, the presence of aromatic groups is an important molecular feature. Moreover, the presence of bulky halogen atoms ortho to the phenolic hydroxy groups increase the inhibitory activity against the AF target enzyme blue mussel phenoloxidase.

The search for the understanding of AF mechanisms becomes a challenge due to the absence of specific molecular targets. Some synthetic compounds appear to impact cellular Ca^2+^ efflux, which is involved in mobility, attachment, and growth of diatoms. Therefore, anti-algal activity of hemibastadins derivatives [55] and indole derivative **254** [83] was justified by the inhibition of transmembrane transport and trigger algal cellular Ca^2+^ efflux. AF activity of capsaicin derivatives were justified by the activation of TRVP1 (Transient receptor potential vanilloid 1) channel, leading to an increase in intracellular Ca^2+^ in algal species, causing deflagellation and, consequently, cell function disorders that impair the biofouling process [135]. Other synthetic compounds appear to show AF activity due to the ability to perturb the integrity of the fouling organisms cell membrane. 3-APS displayed surfactant properties, which led to the cell membrane disruption of the fouling organisms [46]. Capsaicin derivatives displayed antibacterial activity due to its ability to change the cytomembrane fluidity, which led to the disruption of cell membranes increasing membrane permeability [125,168]. Furthermore, some compounds display AF activity due to physical phenomena. In concrete, zosteric acid displays anti-algal activity due to the strong hydrophilicity of the sulfuric acid group, which prevents or reduces the exclusion of the water between the adhesive secreted by spores and the substratum, making the formation of the adhesive-substratum interface difficult [135].

However, several proteins that influence directly or indirectly the biofouling process were also revealed as putative molecular targets. The inhibition of biofim adhesion in *V. carcharie* by anthraquinone derivatives **21**, **27**, **30**, and **39** was explained by the interruption of the quorum sensing signaling system through the interaction of these compounds with the molecular target LuxP, a quorum-sensing synthase in many bacteria also involved in the transportation of autoinducers [39]. The sodium salt of zosteric acid also seems to interfere with quorum sensing signaling system through the interaction with LuxS enzyme [162].

Some compounds were shown to act as inhibitors of AChE enzyme, which displays an important role in the settlement of macrofouling organisms. By blocking neurotransmission, bile acid derivative **64** [46], 3-alkylpyridiunium oligomers and polymers (3-APS) [142,169], and compound **869** [165] decreased the settlement of macrofouling organisms. Hemibastadin derivatives **98**, and **101**–**108** [51,55,56] and *N*-substituted maleimides and succinamides derivatives **441**, **445**, **448**, **452**, **462**, and **468** [11] were shown to be inhibitors of blue mussel phenoloxidase, an enzyme involved in the generation of the plaques of the mussel threads. Isocyanide derivative **270** bound to mitochondrial proteins in *B. neritina* and *A. amphitrite*, affecting mithocondrial functions, which can be responsible for the inhibition of attachment and metamorphosis of macrofouling organisms [95]. Butanolide (compound **373**) was described to act through the interaction with enzymes involved in primary metabolism, in particular, acetyl-CoA acetyltransferase 1 in the barnacle *A. amphitrite*; acyl-CoA dehydrogenase, actin, and glutathione *S*-transferase in the bryozoan *B. neritina*; and succinyl-CoA synthetase β in the marine bacterium *Vibrio* sp. UST020129-010 [103]. The inhibition of larval metamorphosis in the ascidian *C. savignyi* of a natural drimane sesquiterpene, polygodial (compound **684**), was justified for the putative interference with HSP-90 [151]. Five xanthones (**763**, **766**, **773**, **795**, and **797**) were studied by proteomic. Xanthone **773** caused alteration in the abundance of proteins involved in glycolisis/gluconeogenesis, gene translation, lysossomal protein degradation or constituents of cytoskeleton [157]. Xanthone **763** altered the expression of proteins involved in cytoskeleton formation, cell-redox status, and chaperone-mediated regulation, particularly, caused an alteration in the abundance of mussel collagen proteins (PreCols), which play a fulcral role in elasticity, resistance and shock absorber [157]. Xanthone **766** decreased two putative proximal thread proteins (TMPs), localized in byssal threads and responsible for providing them viscoelasticity [157]. Xanthones **795** and **797** altered diverse miosines from the pedal refractor muscle of *M. galloprovincialis* [159].

The increasing understanding of the mechanisms of action underlying the AF effects will lead to the design of more efficient AF compounds, so this is a priority for future research in this area.

Most of the studies determined LC_50_ values against target organisms as a measure of toxicity and calculated the therapeutic ratio (LC_50_/EC_50_). Almost all the synthetic compounds derived from MNPs showed AF activity via nontoxic mechanisms. However, toxicity against non-target organisms is a problem that arises from the use of commercial biocides, but only few studies included toxicity assays against non-target organisms. More efforts are needed in the study of the toxicity of new AF agents against non-target organisms to better predict the ecofriendliness of such compounds.

Nearly 40 of the most potent compounds were studied regarding coating compatibility, controlled release, and in situ field tests. For more details on coating formulations, a recent review was published [170].

The development and synthesis of novel AF compounds towards green chemistry must be taken in consideration in the future. In general, green chemistry comprises twelve principles that aim to achieve a less hazardous chemical synthesis, design safer chemicals and safer solvents, and reduce derivatives [68,171]. Considering that, it is highly valuable to direct research towards creating synthetic routes congruent with the referred principles in order to offer to the marine coatings industry environmentally friendly and sustainable additives.
